# Comprehensive Mapping of Histone Modifications at DNA Double-Strand Breaks Deciphers Repair Pathway Chromatin Signatures

**DOI:** 10.1016/j.molcel.2018.08.020

**Published:** 2018-10-18

**Authors:** Thomas Clouaire, Vincent Rocher, Anahita Lashgari, Coline Arnould, Marion Aguirrebengoa, Anna Biernacka, Magdalena Skrzypczak, François Aymard, Bernard Fongang, Norbert Dojer, Jason S. Iacovoni, Maga Rowicka, Krzysztof Ginalski, Jacques Côté, Gaëlle Legube

**Affiliations:** 1 LBCMCP, Centre de Biologie Integrative (CBI), CNRS, Université de Toulouse, UT3, Toulouse 31062, France; 2 St-Patrick Research Group in Basic Oncology, Laval University Cancer Research Center, Oncology Axis-CHU de Québec-Université Laval Research Center, Quebec City, QC G1R 3S3, Canada; 3 Laboratory of Bioinformatics and Systems Biology, Centre of New Technologies, University of Warsaw, Zwirki i Wigury Warsaw 93, 02-089, Poland; 4 Department of Biochemistry and Molecular Biology, University of Texas Medical Branch, Galveston, TX 77555-0615, USA; 5 Institute of Informatics, University of Warsaw, Banacha 2, 02-097 Warsaw, Poland; 6 Bioinformatic Plateau I2MC, INSERM and University of Toulouse, Toulouse 31062, France

**Keywords:** DNA double-strand breaks, DSB repair, chromatin, histone modifications, non-homologous end joining, homologous recombination, ChIP-seq, histone H1, 53BP1, γH2AX

## Abstract

Double-strand breaks (DSBs) are extremely detrimental DNA lesions that can lead to cancer-driving mutations and translocations. Non-homologous end joining (NHEJ) and homologous recombination (HR) represent the two main repair pathways operating in the context of chromatin to ensure genome stability. Despite extensive efforts, our knowledge of DSB-induced chromatin still remains fragmented. Here, we describe the distribution of 20 chromatin features at multiple DSBs spread throughout the human genome using ChIP-seq. We provide the most comprehensive picture of the chromatin landscape set up at DSBs and identify NHEJ- and HR-specific chromatin events. This study revealed the existence of a DSB-induced monoubiquitination-to-acetylation switch on histone H2B lysine 120, likely mediated by the SAGA complex, as well as higher-order signaling at HR-repaired DSBs whereby histone H1 is evicted while ubiquitin and 53BP1 accumulate over the entire γH2AX domains.

## Highlights

• DSB-chromatin landscape and HR/NHEJ chromatin signatures uncovered by ChIP-seq
• H2BK120 undergoes a switch from ubiquitination to acetylation at a local scale
• H1 is removed and ubiquitin accumulates on entire γH2AX domains, mainly at HR DSB
• 53BP1 spreads over megabase-sized domains, mostly in G1 at HR-prone DSBs

## Graphical Abstract

**Figure:**
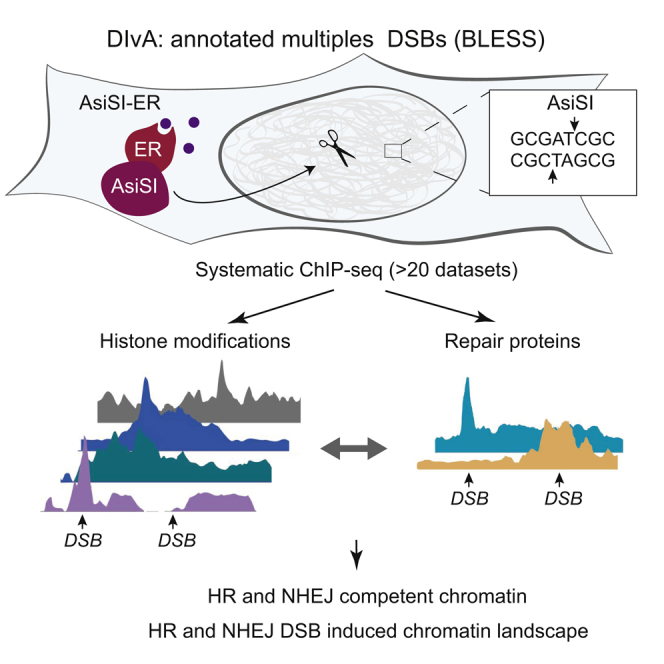

## Teaser

Using ChIP-seq in a cell line where multiple annotated DNA double-strand breaks can be induced on the human genome, Clouaire et al. report a comprehensive view of the chromatin landscape set up at DSBs and decipher the chromatin signature associated with HR and NHEJ repair.

## Introduction

DNA double-strand breaks (DSBs) are extremely detrimental since they can lead to mutations and chromosomal rearrangements. DSBs arise from various environmental stresses and upon developmentally scheduled activation of endonucleases, but they can also arise physiologically during replication and transcription. Anomalies in the DSB repair apparatus are responsible for premature aging and neurodegenerative syndromes and are strongly implicated in cancer onset and progression.

DSBs are mainly repaired by two partially redundant, yet profoundly different, pathways: homologous recombination (HR) and non-homologous end joining (NHEJ) (for review, see Mladenov et al., 2016). HR uses an intact copy of the damaged locus as a template and involves many factors for DSB detection, 5′ end resection, homology search, strand invasion, and resolution. In contrast, NHEJ repair machineries require no or limited resection and can join the two broken ends with no or minimal homology. Inaccuracy, failure, or misuse of each of these pathways can trigger very different consequences on the genome. DSB repair pathway choice can be influenced by cell cycle phase (Hustedt and Durocher, 2016), DNA end complexity (Schipler and Iliakis, 2013), and the type of damaged locus (Clouaire and Legube, 2015, Engel et al., 2018).

In eukaryotes, DSB repair occurs in the context of chromatin. Chromatin is a highly dynamic structure, affected by histone post-translational modifications, DNA methylation, or incorporation of histone variants (for review, see Soshnev et al., 2016, Talbert and Henikoff, 2017). Chromatin modifications can alter the stability of the histone octamer onto DNA but also be specifically recognized by “reader” modules found in chromosomal proteins as well as subunits of DNA transaction machineries. All together, nucleosome modifications regulate DNA accessibility; the stiffness, flexibility, and mobility of chromatin within the nucleus; and the recruitment of molecular machines ensuring transcription, replication, and repair.

Key aspects in the interplay between DSB repair and chromatin environment have already emerged (Table S2). H2AX is rapidly phosphorylated by ATM (and named γH2AX) over several megabases surrounding the break (Caron et al., 2015, Iacovoni et al., 2010, Rogakou et al., 1998, Savic et al., 2009). In parallel, histone acetyltransferases and deacetylases tightly control acetylation levels of several residues of H3, H4, and H2A (Dobbin et al., 2013, Gong et al., 2015, Jacquet et al., 2016, Lee et al., 2010, Miller et al., 2010, Ogiwara et al., 2011, Toiber et al., 2013) to regulate chromatin relaxation near DSBs. Acetylated histones also participate in the recruitment of nucleosome remodeling factors, enhancing DSB accessibility and facilitating resection (Bennett and Peterson, 2015, Lee et al., 2010, Toiber et al., 2013). Similarly, histone methyltransferases and demethylases can regulate recruitment and/or stabilization of repair proteins (CtIP, 53BP1, BRCA1 …) (Table S2). Moreover, ubiquitination and sumoylation pathways contribute to DSB-induced chromatin reorganization (for review, see Schwertman et al., 2016). All together, these specific chromatin modifications generate a chromatin state permissive for repair but also directly contribute to the recruitment of DSB repair machineries, repair pathway choice, and the activation of the DNA damage checkpoint.

Yet the definite map of DSB-induced chromatin modifications and their respective involvement in DSB repair remain largely unknown. Furthermore, NHEJ and HR repair pathways conceivably require very different chromatin settings. Since chromatin structure plays a central role in DNA accessibility and flexibility, an in-depth characterization of the chromatin that assembles at DSBs represents a critical step in understanding how DSB repair machineries operate in the whole nucleus to restore the original DNA sequence and avoid deleterious genome rearrangements.

Here, using high-throughput genomic approaches in a standardized system in which multiple DSBs are induced at defined positions across the human genome, we report the first comprehensive picture of the chromatin landscape induced around DSBs and its relationship with individual repair pathways.

## Results

### High-Resolution Mapping of AsiSI-Induced DSBs

**Figure 1:**
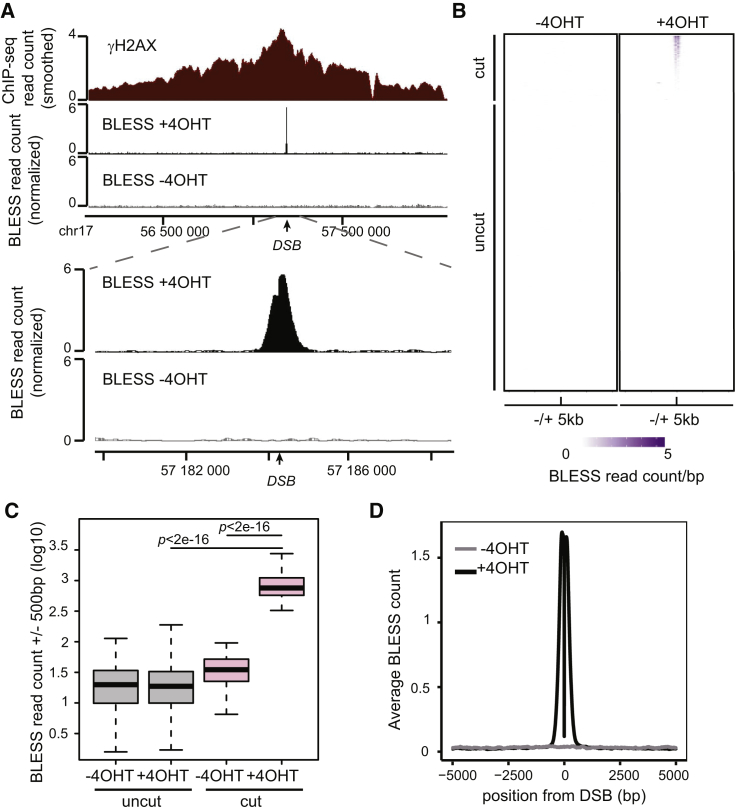
BLESS Mapping of AsiSI-Induced DSB (A) Genome browser screenshot representing γH2AX ChIP-seq and BLESS signal at a single DSB located on human chromosome 17. (B) Heatmap representation of the BLESS profile for the 1,211 predicted AsiSI sites in the human genome in DIvA cells untreated (−4OHT) or treated for 4 hr (+4OHT). DSBs are ordered based on decreasing read count in a 1 kb window. (C) Boxplot representing BLESS signals (1 kb window) for the 80 cut (pink) and the 1,131 uncut (gray) AsiSI sites in untreated (−4OHT) or treated (+4OHT) DIvA cells. p values were calculated using two-sample Wilcoxon tests. (D) Average BLESS count profile for the 80 induced DSBs before (−4OHT, gray line) and after induction (+4OHT, black line).

To characterize the chromatin landscape at DSBs, we used the DIvA (DSB inducible via AsiSI) cell line, which enables the controlled induction (using 4-hydroxytamoxifen, or 4OHT) of multiple well-annotated DSBs scattered throughout the human genome (Aymard et al., 2014, Iacovoni et al., 2010). We previously showed that *in vivo*, AsiSI does not produce a DSB at every target site on the genome and that γH2AX ChIP-seq can serve as a good proxy to identify sites efficiently cut by AsiSI (Aymard et al., 2014, Iacovoni et al., 2010). However, this is an indirect assessment of the genomic lesion, and γH2AX accumulation is strongly affected by genomic and epigenomic features (Caron et al., 2012, Iacovoni et al., 2010). To unambiguously identify the position of AsiSI-induced DSBs and obtain a quantitative measurement of the DSB generation rate, we used the recently developed BLESS method (Crosetto et al., 2013, Mitra et al., 2015). We detected sharp BLESS peaks located precisely at predicted AsiSI sites following a 4 hr treatment of DIvA cells with 4OHT and minimal signal in untreated conditions (Figures 1A and S1A). Further analysis revealed that out of the 1,211 predicted AsiSI sites in the human genome, 174 showed a signal significantly higher than background (Figures S1B and 1B). This is in relatively good agreement with our previous estimates of the number of induced DSBs inferred from γH2AX accumulation and with BLISS data in DIvA cells (Aymard et al., 2014, Iannelli et al., 2017). However, we could detect clear differences between γH2AX and BLESS signals, confirming that γH2AX does not strictly reflect DSB induction rate (Figure S1A, compare left and right panel; Figure S1C). To remove any site that may only be partially cleaved, we focused on a robust set of 80 DSBs defined by BLESS analysis that are significantly induced by 4OHT treatment (Figures 1B–1D, Table S1). These DSBs are characterized by low levels in heterochromatin-associated features such as DNA methylation and H3K9me3 (Figure S1D), and about half are located near active promoters (Figure S1E). Yet analyzing ChIP-seq enrichment for the elongating form of RNA polymerase II (S2P) revealed that not all sites necessarily reside within actively transcribed regions (Figure S1D). We retained this validated set of DSBs, representing both active and inactive euchromatic regions, for further analysis.

### Mapping of Histone Modifications at AsiSI-Induced DSBs

To obtain a comprehensive picture of the chromatin landscape at DSBs, we generated 20 ChIP-seq profiles, in both damaged (4OHT-treated) and undamaged cells. These included linker and core histones, histone variants, and post-translational modifications that were proposed to play a role in DSB repair (Table S2). We also generated a genome-wide map for chromatin-associated ubiquitin using the FK2 antibody that recognizes a wide variety of ubiquitin conjugates. ChIP-qPCR revealed that each antibody efficiently immunoprecipitated chromatin (Figure S2A). More importantly, we could recapitulate previously reported profiles for each feature over transcription units, categorized by transcriptional activity (Figure S2B). Our H4S1P mapping, which was lacking in mammalian cells, is in agreement with its proposed function in transcriptional regulation and accumulation over gene bodies in yeast (Utley et al., 2005).

**Figure 2:**
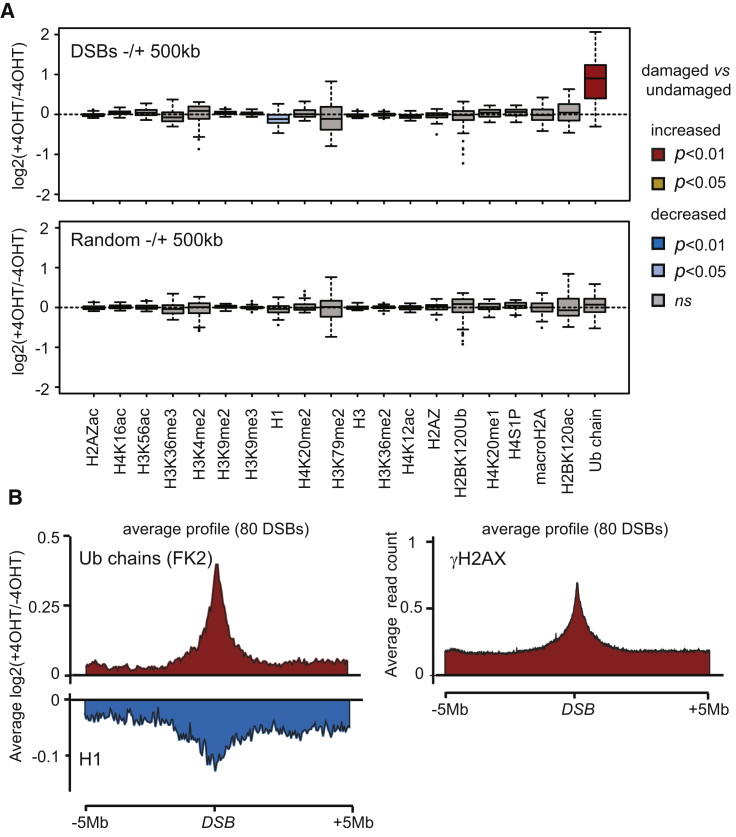
Large-Scale DSB-Induced Chromatin Changes (A) Boxplot representing the ChIP-seq enrichment ratio between 4OHT-treated and untreated DIvA cells (expressed as a log_2_ ratio) for 80 DSBs (upper panel) or 80 randomly picked genomic regions (lower panel) over a 1 Mb window. Boxes are colored according to p values (two-sample Wilcoxon tests) and the nature of the change. (B) Left: Average profile of the enrichment between 4OHT-treated and untreated DIvA cells for ubiquitin and H1 over 80 DSBs in a 10 Mb window. Values are expressed as log_2_ ratios. Right: Average profile of γH2AX in 4OHT-treated DIvA cells.

Given that γH2AX accumulation can spread up to two megabases (Mb) from the actual breakpoint (Iacovoni et al., 2010), we first examined DSB-induced chromatin modifications over a 1 Mb window. At this scale, ubiquitination is largely induced (p < 0.01, two-sample Wilcoxon test) and histone H1 occupancy significantly reduced (p < 0.05, two-sample Wilcoxon test) compared to randomly selected genomic regions (Figure 2A). We could not detect significant DSB-induced changes for any other histone modifications within these megabase-sized genomic windows surrounding breaks. Average profiles revealed that ubiquitin conjugates accumulated over approximately 2 Mb, similarly to γH2AX spreading (Figures 2B and S2C). DSB-induced H1 depletion is maximal over a similar region but remains detectable over a slightly more extended area (Figure 2B, bottom panel, and Figure S2C). All together, this shows that DSBs induce few but large-scale (megabase-sized) chromatin reshufflings, such as phosphorylation of H2AX, accumulation of ubiquitin, and depletion of the linker histone H1.

**Figure 3:**
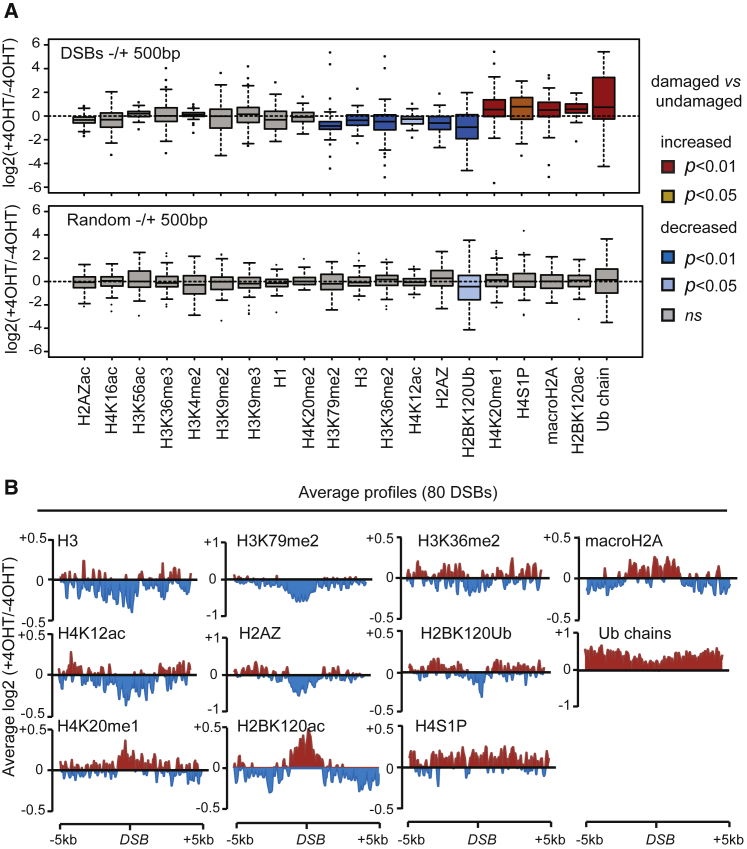
Narrow-Scale DSB-Induced Chromatin Changes (A) Boxplot representing the ChIP-seq enrichment ratio between treated and untreated DIvA cells (expressed as a log_2_ ratio) for 80 DSBs (upper panel) or 80 randomly picked genomic regions (lower panel) over a 1 kb window. Boxes are colored according to p values (two-sample Wilcoxon tests) and the nature of the change. (B) Average profile of the enrichment between treated and untreated DIvA cells for 12 modifications, showing significant differences between each condition over 80 DSBs in a 10 kb window. Values are expressed as log_2_ ratios.

We next considered that DSB could cause chromatin modifications much closer to the break and examined 1 kb windows surrounding AsiSI-induced DSBs. Interestingly, we found that among the 20 chromatin modifications analyzed, 6 were significantly decreased (H3K79me2, H3, H3K36me2, H4K12ac, H2AZ, and H2BK120ub) and 5 significantly increased (H4S1P, H4K20me1, macroH2A, H2BK120ac, and ubiquitin) near DSBs (Figure 3A, top panel) when compared to random sites (Figure 3A, bottom panel). Average profiles showed that these proximal DSB-induced chromatin alterations spread over different scales, ranging from 1 to 10 kb (Figure 3B). Decreases in H3, H2AZ, H4K12ac, H2BK120ub, and H3K79me2 and the increases in H4K20me1 and H2BK120ac took place over 2–4 kb windows. MacroH2A and H4S1P showed a DSB induction spanning 5–6 kb and approximately 10 kb, respectively. In most cases, normalizing to histone H3 did not alter the observed changes, with the exception of H4K12ac and H3K36me2, which may thus only reflect changes in nucleosome occupancy (Figure S2D). All together, our systematic ChIP-seq mapping indicates that DSBs trigger many alterations to chromatin structure occurring over many different scales, ranging from 1 kb up to several megabases.

More specifically, our data suggest that in response to DSBs, H2BK120 undergoes a switch from ubiquitination to acetylation (Figure 3A). Time-course experiments confirmed the progressive loss of H2BK120ub upon DSB induction (Figure S3A). Interestingly, SAGA, a prominent chromatin-modifying complex, acetylates histones H3 and H2B and also displays deubiquitinase activity toward H2BK120 (Helmlinger and Tora, 2017). SAGA has also been previously implicated in class switch recombination and response to ionizing radiation (Ramachandran et al., 2016). We show that *in vitro*, the native human SAGA complex (affinity-purified through its specific SUPT7L subunit; Figure S3B) displays both H2BK120 deubiquitinase and acetyltransferase activities, making it a primary candidate for this switch (Figure S3C). Furthermore, depletion of SUPT7L as well as PCAF, one HAT paralog present in the SAGA complex (Figures S3D and S3E), triggered decreases in both HR at an endogenous locus (*LMNA*) following CRISPR/Cas9 breakage (Pauty et al., 2017) and NHEJ in a cell reporter system (Jacquet et al., 2016) (Figures S3F and S3G), indicating that SAGA indeed contributes to DSB repair in human cells.

### Damage in Active Chromatin Undergoes Preferential Repair via HR

**Figure 4:**
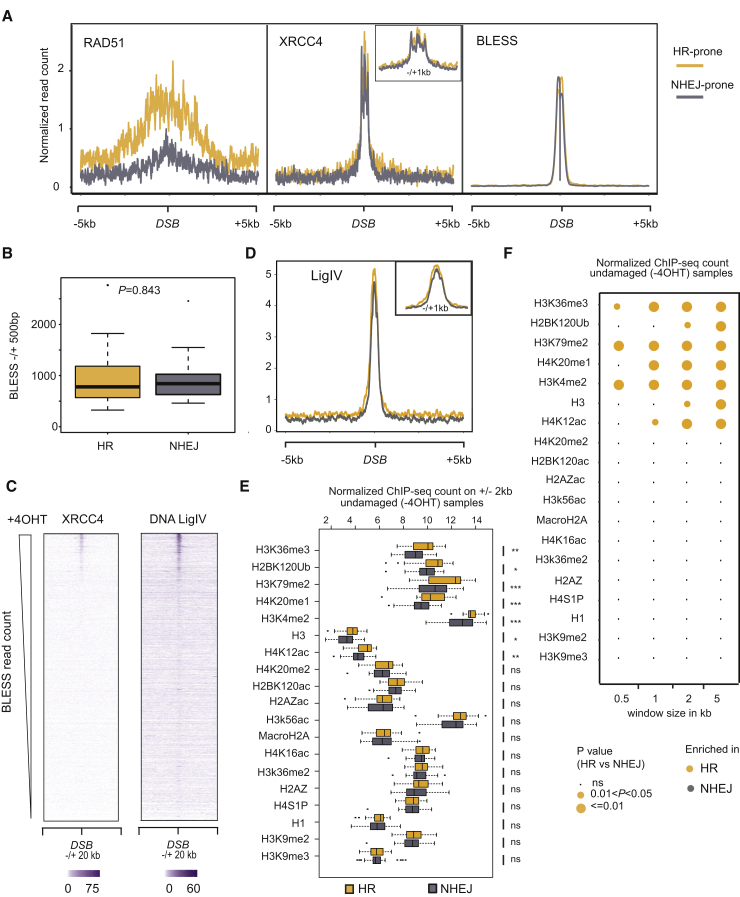
Chromatin Features Associated with HR-Prone DSBs (A) Average profile for RAD51 ChIP-seq, XRCC4 ChIP-seq, and BLESS for 30 HR (yellow) or 30 NHEJ (gray) DSBs. HR and NHEJ DSBs were defined based upon the RAD51/XRCC4 binding ratio (see STAR Methods). (B) Boxplot representing BLESS read count (1 kb window) around 30 HR or 30 NHEJ DSBs. p value was calculated using two-sample Wilcoxon test. (C) Heatmap representing the XRCC4 and DNA Ligase IV ChIP-seq signals on a 40 kb window centered around all AsiSI sites, ordered based on the BLESS level. (D) Same as (A) for DNA Ligase IV ChIP-seq. (E) Boxplot representing the ChIP-seq read count (4 kb window) for each histone modification in untreated cells for 30 HR (yellow) and 30 NHEJ (gray) DSBs. p values were calculated using two-sample Wilcoxon test. ^∗^p < 0.05, ^∗∗^p < 0.01; p > 0.05 is not significant (ns). (F) Circle plot representing p values (from two-sample Wilcoxon test) when comparing ChIP-seq signal for HR and NHEJ DSB using increasing window size.

It is now well established that the local chromatin structure can influence how a given DSB is handled and subsequently repaired (for review, see Clouaire and Legube, 2015). We thus set out to understand the contribution of chromatin to repair pathway choice by defining chromatin states favorable to HR and NHEJ. We defined subsets of BLESS-validated DSBs that are preferentially repaired by HR or NHEJ (30 in each category) by sorting them according to the binding ratio for RAD51 and XRCC4 using ChIP-seq data obtained in 4OHT-treated DIvA cells (Aymard et al., 2014). Average profiles confirmed the prominent accumulation and spreading of RAD51 over 5 kb at DSBs preferentially repaired by HR (Figure 4A). On the other hand, XRCC4 recruitment appeared similar for both sets of DSBs, with a multimodal distribution over 500 bp (Figures 4A and S4A), likely related to XRCC4/XLF filaments at DSBs (Ropars et al., 2011). Importantly, BLESS intensity was comparable between HR and NHEJ DSBs (Figures 4A and 4B), suggesting that differences in RAD51 binding do not solely depend on the DSB induction rate. Since NHEJ is a relatively fast process compared to HR, it remained possible that some sites could be cleaved, repaired, and mutated with a very fast kinetics, therefore not appearing in our set of NHEJ-prone DSBs. To address this, we performed XRCC4 ChIP-seq at various times of 4OHT treatment (1 hr, 4 hr, and 24 hr) and found that XRCC4 binding was indeed very similar at DSB sites at each time point (Figures S4C–S4E). More importantly, we could not detect any site showing XRCC4 enrichment at 1 hr or 24 hr and not at 4 hr, which strongly validated our HR and NHEJ categories (Figures S4C–S4E). To confirm that XRCC4 binding was indeed indicative of NHEJ, we also performed ChIP-seq using an antibody directed against DNA ligase IV, another key component in this pathway. As expected, DNA ligase IV showed a very similar enrichment when compared to XRCC4 at all AsiSI sites (Figures 4C, 4D, and S5A–S5C). All together, these analyses allowed us to define a robust set of DSBs either prone to engage HR (HR-prone) or not (NHEJ-prone).

Next, we compared basal enrichment (before DSB induction) for each modification at the vicinity of DSBs (2 kb) preferentially repaired by HR or NHEJ. In agreement with our previous finding (Aymard et al., 2014), H3K36me3 was significantly more abundant proximal to DSBs repaired by HR (Figure 4E). HR-competent chromatin also contained elevated levels of H3K79me2, H4K20me1, H2BK120ub, H3K4me2, H4K12ac, and core histone H3 (Figure 4E). This result was confirmed when comparing chromatin signatures over increasing window sizes (Figure 4F). Consistently, we found a similar trend when using DNA ligase IV instead of XRCC4 to determine HR and NHEJ categories (Figure S5D). On the other hand, we found no evidence of a specific signature that may actively favor repair by NHEJ (Figures 4E, 4F, and S5D). All together, these data identified a specific chromatin structure that is competent for HR repair. The HR-specific signature primarily consists of features linked to active transcription (Figure S2B). In agreement, HR-prone DSBs were found to be significantly enriched in the nuclear compartment A1, which was defined by its specific long-range interaction pattern by Hi-C analysis and shown to be enriched in transcriptionally active regions (Rao et al., 2014) (Figure S4B). Hence, our data strongly favor the hypothesis that DSBs induced in active genes are biased toward HR.

### Chromatin Modifications upon HR and NHEJ Repair: Deciphering the Repair Pathway Chromatin Signature

**Figure 5:**
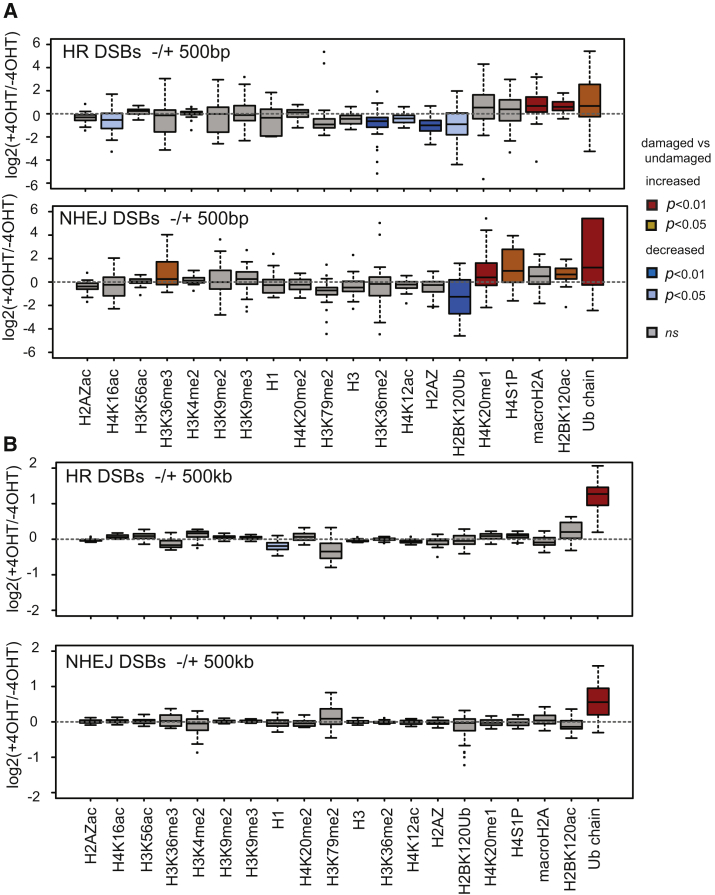
HR and NHEJ-Induced Chromatin Changes (A) Boxplot representing the ChIP-seq enrichment ratio between treated and untreated DIvA cells (expressed as a log_2_ ratio) for 30 HR (upper panel) or 30 NHEJ (lower panel) over a 1 kb window. Boxes are colored according to p values (two-sample Wilcoxon tests) and the nature of the change. (B) Same as (A) for a 1 Mb window.

DSB repair by HR and NHEJ depends on two separate mechanisms and operates in distinct chromatin contexts (see above). Hence, it is possible that each pathway involves different remodeling events compatible with its particular repair mechanism. We therefore considered the possibility that DSB-induced alterations in chromatin could be specific for HR- and NHEJ-prone DSBs. Notably, focusing on events occurring proximal to the break point (1 kb windows), we found that while some chromatin marks significantly changed after break induction only at DSBs repaired by HR or by NHEJ, others occurred irrespective of the repair mechanism (Figure 5A). We also examined DSB-induced chromatin changes at HR- and NHEJ-prone sites at various distances from the DSBs (Figure S5E). MacroH2A deposition and H4S1 phosphorylation occurred upon repair by both HR and NHEJ (Figures 5A and S5E). Similarly, the switch from H2BK120 ubiquitination to acetylation also occurred at all DSBs (Figures 5A and S5E), in agreement with our above finding that depletion of the SAGA complex affects both HR and NHEJ (Figures S3F and S3G). In contrast, reduction in H2AZ and H3 occupancy and decrease in H3K36/79 dimethylation and in H4K12/K16 acetylation following DSB induction were significant only at sites prone to HR (Figures 5A and S5E). Conversely, DSB induction at sites repaired by NHEJ was accompanied by a significant increase in H3K36me3 and H4K20me1, modifications not found at HR-repaired DSBs (Figures 5A and S5E). Hence, we were able to define a local histone modification signature, which occurs proximal to DSBs, associated with the two main repair pathways.

**Figure 6:**
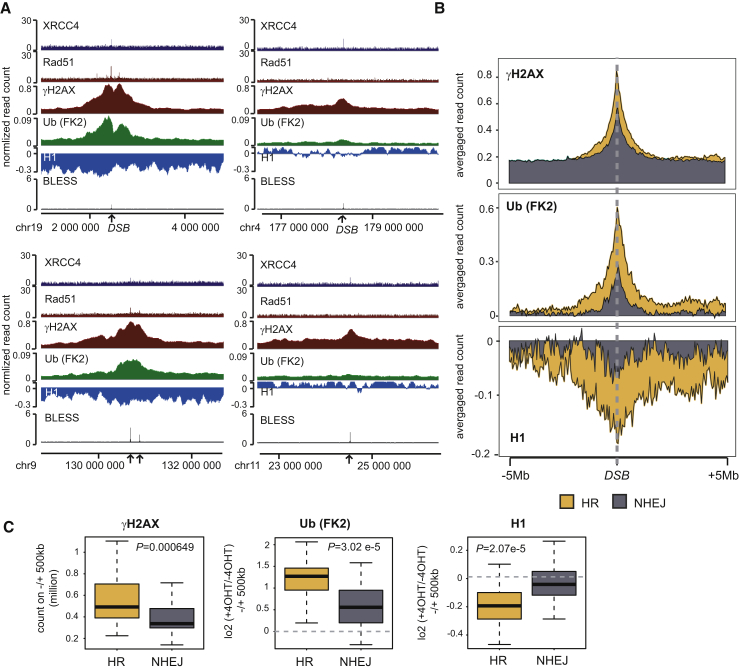
Acute Signaling at HR-Prone DSBs (A) Genome Browser screenshots representing ChIP-seq signals for XRCC4, RAD51, γH2AX, ubiquitin, H1, and BLESS for two HR-DSBs (left) and two NHEJ DSBs (right). Data are expressed as read count (from 4OHT-treated samples) for XRCC4, RAD51, and γH2AX ChIP-seq and BLESS. Data for ubiquitin (FK2) and H1 are expressed as a log_2_ ratio between treated and untreated cells. (B) Average profile for γH2AX ChIP-seq (read count in OHT treated samples), ubiquitin, and H1 (as a log_2_ ratio between treated and untreated cells) for 30 HR (yellow) and 30 NHEJ (gray) DSBs in a 10 Mb window. (C) Boxplot representing ChIP-seq read count from treated cells (γH2AX) or log_2_ ratio between treated and untreated cells (ubiquitin [FK2] and H1) for 30 HR (yellow) or 30 NHEJ (gray) DSBs in a 1 Mb window. p values were calculated using two-sample Wilcoxon tests.

We also interrogated our data for pathway-specific events occurring within 1 Mb from the break. Linker histone H1 depletion and ubiquitin accumulation were significantly more pronounced within 1 Mb of HR-repaired sites compared to NHEJ-prone DSBs (Figure 5B). Examination of individual (Figure 6A) or averaged (Figure 6B) profiles confirmed that DSB-induced changes in H1 and ubiquitin were accentuated at HR sites, with H1 depletion being barely detectable at NHEJ sites. We also detected increased megabase-wide γH2AX levels for HR sites compared to NHEJ sites (Figures 6A and 6B). Accumulation of ubiquitin conjugate, H2AX phosphorylation, and H1 depletion were indeed significantly reinforced surrounding DSBs repaired by HR compared to those repaired by NHEJ (Figure 6C). This finding suggests that damage occurring in active chromatin and repaired by HR can trigger acute and broad-scale remodeling within high-order chromatin structure that is likely to drastically alter its properties.

### Acute High-Order Chromatin Modifications Correlate with Enhanced 53BP1 Recruitment *In Vivo*

**Figure 7:**
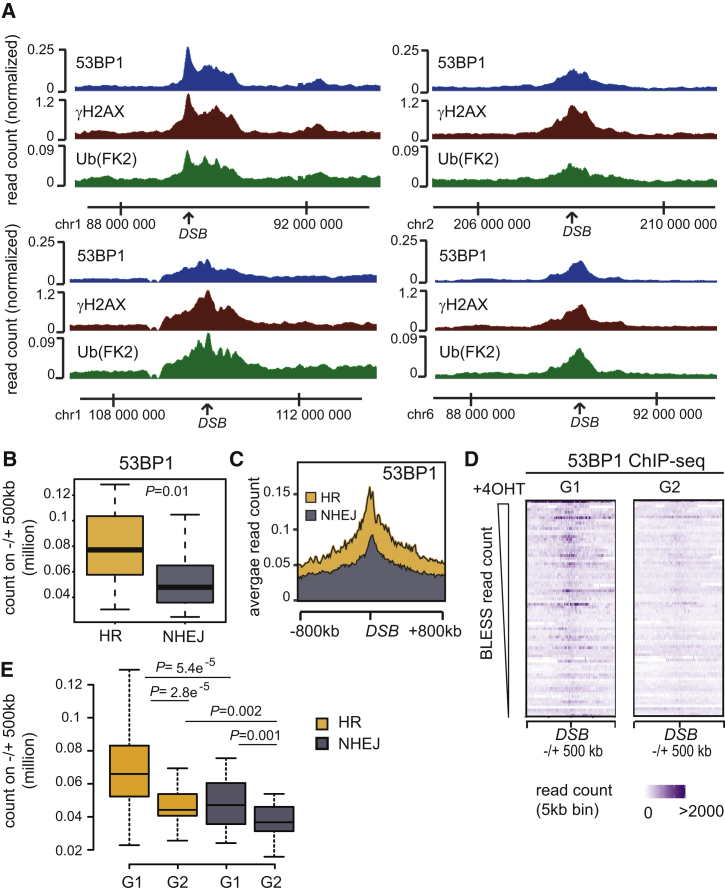
53BP1 Preferentially Accumulates at HR-Prone DSBs Specifically in G1 (A) Genome Browser screenshots representing ChIP-seq signals (from 4OHT-treated samples) for 53BP1, γH2AX, and ubiquitin. (B) Boxplot representing 53BP1 enrichment in a 1 Mb window for 30 HR (yellow) and 30 NHEJ (gray) DSBs. p value was calculated using two-sample Wilcoxon test. (C) Average profile for 53BP1 ChIP-seq (read count in 4OHT-treated cells) for 30 HR (yellow) and 30 NHEJ (gray) DSBs in a 1.6 Mb window (upper panel). (D) Heatmap representing 53BP1 ChIP-seq count obtained in G1- and G2-synchronized cells as indicated, on a 1 Mb window surrounding 80 DSBs induced by AsiSI. DSBs are sorted by BLESS read counts. (E) Boxplot representing 53BP1 enrichment in a 1 Mb window for 30 HR (yellow) and 30 NHEJ (gray) DSBs in G1- and G2-synchronized cells as indicated. p values were calculated using two-sample Wilcoxon test.

An obvious role for chromatin-mediated DSB signaling would be to directly attract effectors of the DDR. 53BP1 plays key roles in DSB repair by inhibiting end resection, favoring distal end synapsis, and promoting damaged chromatin mobility (for review, see Panier and Boulton, 2014). 53BP1 interaction with damaged chromatin involves multivalent engagement of several histone modifications such as H2A (or H2AX) lysine 15 ubiquitination and methylation of lysine 20 on histone H4 (Fradet-Turcotte et al., 2013, Wilson et al., 2016). Furthermore, stable 53BP1 association with DSBs may involve a direct interaction with γH2AX (Kleiner et al., 2015, Ward et al., 2003) and can be regulated by acetylation on H4 and H2A (Jacquet et al., 2016, Tang et al., 2013). We generated a genome-wide ChIP-seq map of 53BP1 binding to damaged chromatin *in vivo*. Inspection of 53BP1 distribution around individual DSBs (Figure 7A) or averaged over the 80 DSBs (Figure S6A) revealed a striking ability for 53BP1 to spread within megabase-wide domains. Furthermore, 53BP1 binding profiles appear almost indistinguishable from those of γH2AX and ubiquitin (Figures 7A and S6A), and we observed a very strong correlation between the accumulation of γH2AX, ubiquitin, and 53BP1 within 1 Mb domains surrounding DSBs (Figure S6B). We also observed that 53BP1 is significantly more enriched within a megabase from sites preferentially repaired by HR compared with sites repaired by NHEJ (Figures 7B, 7C, and S6C). Thus, our data suggest that DSBs occurring in active chromatin, which preferentially undergo HR repair, can trigger an acute high-order chromatin signaling that may favor subsequent recruitment of 53BP1 over megabase-sized regions encompassing the break site.

*In vivo*, 53BP1 was shown to inhibit 5′ end resection in various contexts, including class switch recombination and dysfunctional telomeres fusion (for review, see Panier and Boulton, 2014). Yet our results suggest that 53BP1 binding is stronger at sites that are more prone to be repaired by HR, a process that requires end resection. To clarify this apparent discrepancy, we generated 53BP1 ChIP-seq data in damaged G1- and G2-synchronized cells. Overall, we found that 53BP1 accumulation was much stronger during G1 compared to G2, (Figures 7D and S6D–S6F). Because γH2AX appeared rather similar between both phases of the cell cycle (Figure S6F), this suggests that the striking difference in 53BP1 spreading over megabase-wide domains observed when comparing G1 and G2 cells is unlikely to be due to differences in DSB induction rates. Finally, we confirmed that 53BP1 accumulation is more prominent at DSBs repaired by HR in G1 compared to G2, while signal for NHEJ DSB remained consistently lower regardless of the cell cycle phase (Figure 7E). Thus, our data revealed that 53BP1 binding is favored at HR-prone DSBs, but mostly in G1, when HR usage is restricted. This suggests that 53BP1 exerts its anti-resection function in G1 only at sites prone to undergo HR, while it may be less critical at DSBs usually repaired by NHEJ.

## Discussion

### A Standardized Approach to Investigate DSB-Induced Chromatin Changes

Here, we provide the most comprehensive view to date of the chromatin landscape assembled at DSBs in human cells. By analyzing 20 chromatin features that were previously shown to be involved in DSB repair, we were able to substantiate several earlier findings such as H3, H2AZ, and H1 removal, macroH2A incorporation, H4S1 phosphorylation, H4K16 deacetylation, H4K20 monomethylation, and the accumulation of ubiquitin conjugates at sites of damage (Table S2). Interestingly, DSB-induced phosphorylation of histone H4 has only been previously reported in budding yeast and was proposed to promote NHEJ (Cheung et al., 2005, Utley et al., 2005). Break-induced H4 phosphorylation is therefore conserved in higher eukaryotes and spreads over large regions spanning up to 10 kb from the DSB, independently of the repair pathway. This ability to propagate up to 50 nucleosomes away from the break suggests that H4 phosphorylation functions in DDR signaling, potentially as a recruitment platform for DDR proteins.

Here we failed to detect any change reported by others (Table S2) in H3K9me2, H3K9me3, H4K20me2, or H3K56ac. Conversely, we observed a clear loss of H3K79me2 and H2BK120ub, two histone modifications that were found, respectively, unchanged and increased at sites of breaks (Huyen et al., 2004, Moyal et al., 2011, Nakamura et al., 2011). Such discrepancies could arise from the use of different DSB induction methods that can produce chemically different DNA ends, concomitantly trigger other forms of DNA damage, or create DSBs at a given cell cycle stage. More importantly, DSB-inducing agents do not necessarily damage the same regions across the genome, which will strongly influence the chromatin outcome. Indeed, X- and γ-rays produce DSBs randomly throughout the genome, mainly in untranscribed and potentially heterochromatic regions, while AsiSI mostly introduces DSBs within or near genes, either active or inactive, leaving heterochromatin undamaged. This could explain why neocarzinostatin and ionizing radiation, unlike AsiSI-induced DSBs, trigger an increase in H2BK120ub levels (Moyal et al., 2011, Nakamura et al., 2011). Kinetic aspects should also be considered. We performed our ChIP 4 hr after AsiSI activation, a time point representing an “equilibrium” in which events ranging from early cleavage to late repair cohabit within the cell population. However, this may preclude the identification of very transient and/or very late events. Finally, in order to identify the most prominent DSB-induced chromatin features, we purposely limited our large-scale analysis to an averaged snapshot on a large population of asynchronous cells, which may hinder the identification of events occurring at specific cell cycle stages. Yet despite a few discrepancies with the published literature, our study allowed us to highlight major chromatin changes during DSB repair and to associate these changes with a preferred repair pathway. Additional studies in repair-deficient cells should now be conducted in order to establish whether these histone modification changes do indeed depend on HR or NHEJ repair or only on the initial chromatin status. Moreover, systematic mapping of chromatin-modifying enzymes following DNA damage will be required to better understand the establishment of this multiscale chromatin landscape. Investigating if, when, and how this DSB-induced chromatin landscape is reverted following repair in order to maintain epigenome stability also represent exciting follow-up studies.

### Histone Crosstalks Involved in Transcription Regulation Are Mobilized in the DSB Response

Importantly, our data fully recapitulate several crosstalks previously identified in the context of chromatin organization and transcriptional regulation. Indeed, our study reveals that upon DSB induction, macroH2A is incorporated (Khurana et al., 2014), and H2BK120 undergoes a switch from ubiquitination to acetylation. MacroH2A can regulate gene expression by stimulating CBP-dependent H2BK120 acetylation (Chen et al., 2014), which precludes H2BK120 ubiquitination. Our findings suggest that this macroH2A-H2BK120ac crosstalk goes beyond transcriptional regulation and may also function following DNA damage and repair. Our work revealed that the transition from ubiquitination to acetylation on H2BK120 may be mediated by the SAGA complex, a well-known complex involved in transcriptional regulation, and that depletion of DUB and HAT subunits of SAGA leads to defective HR and NHEJ, in agreement with a previous study (Ramachandran et al., 2016). Notably, these macroH2A-H2BK120 changes occur independently of the DSB considered (i.e., prone to be repaired by HR or NHEJ), suggesting that they take place before repair reaction is engaged. Given the function of H2BK120ub in nucleosome stabilization (Batta et al., 2011, Fleming et al., 2008), deubiquitinating H2B may loosen nucleosome stability onto DNA, thereby favoring subsequent remodeling and downstream repair events.

We also identified seven additional chromatin changes after DSB induction, including decreases in H3K79me2, H2AZ, and H4K12/K16ac, which specifically occurred at DSBs prone to HR (Figure S7). We also recently demonstrated that H3K4me3 decreases at AsiSI-induced DSBs (Gong et al., 2017). Notably, all these modifications were already found to be coordinated and linked to H2B ubiquitination during transcriptional regulation. For example, H2BK120ub is required for H3K79 methylation (McGinty et al., 2008, Sun and Allis, 2002) and counteracts INO80-dependent H2AZ removal (Segala et al., 2016). Finally, H2B ubiquitination stimulates H3K4 trimethylation (Sun and Allis, 2002, Zhu et al., 2005). Our data indicate that this well-established crosstalk likely also functions at DSBs and that the switch from H2BK120 ubiquitination to acetylation at DSBs may subsequently induce H3K79 and H3K4me3 demethylation, H4 deacetylation, and H2AZ removal by INO80, which itself associates with DSB (Downs et al., 2004, Morrison et al., 2004, van Attikum et al., 2004). Since H2AZ occupancy was found to regulate end resection (Alatwi and Downs, 2015, Gursoy-Yuzugullu et al., 2015, Xu et al., 2012), it is possible that these coordinated modifications of H2BK120ub-H3K79me2-H2AZ-H4 at HR-prone DSBs contribute to the establishment of a chromatin state competent for resection and/or Rad51 nucleofilament assembly.

### Megabase-Scale Chromatin Signaling: A Central Unit in the DSB Response

Chromatin flanking DSBs also undergoes extensive large-scale remodeling, such as γH2AX spreading over megabase-wide domains (Iacovoni et al., 2010, Rogakou et al., 1998, Savic et al., 2009). Here we show that this large-scale remodeling is also accompanied by the accumulation of ubiquitin conjugates, 53BP1 accrual, and the removal of histone H1 from the entire γH2AX domain. 53BP1 is known to be recruited to damaged chromatin by engaging multiple interactions with H2AK15ub and H4K20me2 but also with γH2AX (for review, see Panier and Boulton, 2014). Our data support this proposed mode of recruitment given that: (1) 53BP1 strongly parallels the pattern observed with γH2AX and the FK2 antibody (although the broad specificity of this antibody toward many ubiquitinated substrates precludes a definitive conclusion regarding the nature of the modified protein) and (2) 53BP1 association with DSB is minimal in G2, in agreement with the proposed dilution of H4K20me2 on post-replicative chromatin (Pellegrino et al., 2017). H1, on the other hand, was recently shown to be displaced from sites of DNA damage (Sellou et al., 2016, Strickfaden et al., 2016). Interestingly, RNF8-mediated H1 ubiquitination loosens its interaction with chromatin (Thorslund et al., 2015), providing potential mechanisms for H1 eviction from megabase-wide γH2AX domains. These megabase-wide chromatin modules display well-defined boundaries that may correlate with topologically associated domains (TADs) (Caron et al., 2012, Marnef and Legube, 2017), a basic unit of chromosome folding in 3D. The establishment of these large chromatin changes might therefore be determined by the initial chromosome structure (for review, see Aymard and Legube, 2016, Marnef and Legube, 2017). Such profound changes in entire TADs could regulate the chromatin fiber physical properties such as the mobility of the damaged DNA within the nucleus. In agreement, linker histones strongly affect the global chromatin structure (Fan et al., 2005), and 53BP1 favors long-range motions of DNA ends (Dimitrova et al., 2008).

### The Complex Fate of DSBs in Active Transcription Units

DSB repair varies across the genome. Damage in heterochromatin is largely repaired by HR in G2 by a specific pathway requiring 53BP1-dependent chromatin relaxation, followed by its relocation at the periphery of IR-induced foci (Kakarougkas et al., 2013, Noon et al., 2010). In euchromatin, only transcriptionally active genes, when damaged, are prone to HR repair in G2 (Aymard et al., 2014; this study). Importantly, damaged active genes are refractory to repair in G1, where they instead persist and cluster together within foci (Aymard et al., 2017). Here we found that megabase-size chromatin modifications are more prominent at sites prone to be repaired by HR, e.g., DSBs occurring within active chromatin domains. Such an acute signaling could be indicative of a specific DDR pathway mobilized upon damage in active regions of the genome. Additionally, we found that these DSBs also display enhanced binding of 53BP1, specifically during G1. We did not observe specific 53BP1 accumulation or redistribution at HR-prone DSBs during G2 in our study, suggesting that 53BP1 is likely dispensable to promote HR in the euchromatic, relaxed fraction of the genome. 53BP1 may interact with DSBs to inhibit extensive end processing in G1 and avoid the use of deleterious pathways such as alt-NHEJ during times when canonical HR is not available (Biehs et al., 2017).

In summary, we provide here the first comprehensive depiction of the set of histone modifications induced upon DSB associating with HR and NHEJ repair and report a thorough description of HR-competent chromatin, which is strongly linked with transcriptional activity. Active genes emerge as particularly fragile loci that frequently experience DNA double-strand breakage (for review, see Marnef et al., 2017) and represent translocation hotspots. Characterizing the local and large-scale chromatin structures that assemble at these specific damaged loci represents the first step to understanding how chromatin mobility may change following damage to promote DSB clustering and homology search and, in some instance, lead to translocations.

## STAR★Methods

### Key Resources Table

**Table:** 

REAGENT or RESOURCE	SOURCE	IDENTIFIER
**Antibodies**		

Anti-H4	Abcam	Cat# ab7311, RRID:AB_305837
Anti-H2B	Abcam	Cat# ab1790, RRID:AB_302612
Anti-H3	Abcam	Cat# ab1791, RRID:AB_302613
Anti-H2AZ	Abcam	Cat# ab4174, RRID:AB_304345
Anti-H1	Abcam	Cat# ab17677, RRID:AB_2117984
Anti-macroH2A1	Millipore	Cat# 07-219, RRID:AB_310439
Anti-H2AZac	Abcam	Cat# ab18262, RRID:AB_873820
Anti-H3K79me2	Active Motif	Cat# 39143, RRID:AB_2561018
Anti-H4K20me1	Active Motif	Cat# 39727, RRID:AB_2615074
Anti-H3K9me2	Abcam	Cat# ab1220, RRID:AB_449854
Anti-H3K9me3	Abcam	Cat# ab8898, RRID:AB_306848
Anti-H3K4me2	Millipore	Cat# 07-030, RRID:AB_10099880
Anti-H3K36me2	Abcam	Cat# ab9049, RRID:AB_1280939
Anti-H3K36me3	Abcam	Cat# ab9050, RRID:AB_306966
Anti-H4K12ac	Abcam	Cat# ab46983, RRID:AB_873859
Anti-H4K16ac	Millipore	Cat# 07-329, RRID:AB_310525
Anti-H3K56ac	Abcam	Cat# ab76307, RRID:AB_1523762
Anti-H4S1P	Novus	Cat# NB21-2000, RRID:AB_11019163
Anti-H4K20me2	Abcam	Cat# ab9052, RRID:AB_1951942
Anti-H2BK120Ub	Cell Signaling	Cat# 5546, RRID:AB_106934
Anti-H2BK120ac	Millipore	Cat# 07-564, RRID:AB_11213734
Anti-Ubiquitinylated proteins	Millipore	Cat# 04-263, RRID:AB_612093
Anti-γH2AX	Abcam	Cat# ab81299, RRID:AB_1640564
Anti-XRCC4	Abcam	Cat# ab145, RRID:AB_301278
Anti-RAD51	Santa Cruz	Cat# H-92, RRID:AB_2253533
Anti-53BP1	Novus	Cat# NB100-305, RRID:AB_10001695
Anti-DNA Ligase IV	Genetex	Cat# GTX55592

**Chemicals, Peptides, and Recombinant Proteins**		

(Z)-4-Hydroxytamoxifen	Sigma	Cat# H7904
Thymidine	Sigma	Cat# T1895

**Critical Commercial Assays**		

Quick-RNA MicroPrep kit	Zymo Research	Cat# R1050
qScript cDNA synthesis kit	Quanta Bio	Cat# 95047-100
Lipofectamine RNAiMAX	Invitrogen	Cat# 13778075
SE. Cell Line 4D-Nucleofector X Kit	Lonza	Cat# V4XC1012

**Deposited Data**		

Raw data (ChIP-seq and BLESS)	This paper	ArrayExpress: E-MTAB-5817
RAD51 and XRCC4 ChIP-seq	Aymard et al., 2014	ArrayExpress E-MTAB-1241
RNA polymerase II S2P ChIP-seq	Cohen et al., 2018	ArrayExpress E-MTAB-6318
MethylCap-seq	Deplus et al., 2014	GEO GSE26810

**Experimental Models: Cell Lines**		

DIvA cell	Iacovoni et al., 2010	N/A
U20S-ISceI GFP-RFP (NHEJ)	Jacquet et al., 2016	N/A
3xFlag-Twin-strep-tagged SUPT7L K562	Dalvai et al., 2015	N/A
U2OS	N/A	ATCC HTB-96, RRID:CVCL_0042

**Oligonucleotides**		

HR-DSB1 for ChIP-qPCR FW GATTGGCTATGGGTGTGGAC REV CATCCTTGCAAACCAGTCCT	Aymard et al., 2014	N/A
HR-DSB2 for ChIP-qPCR FW CCGCCAGAAAGTTTCCTAGA REV CTCACCCTTGCAGCACTTG	Aymard et al., 2014	N/A
NHEJ-DSB for ChIP-qPCR FW TGCCGGTCTCCTAGAAGTTG REV GCGCTTGATTTCCCTGAGT	Aymard et al., 2014	N/A
ACTB for ChIP-qPCR FW AGCCGGGCTCTTGCCAAT REV AGTTAGCGCCCAAAGGACCA	This paper	N/A
TAF12 for ChIP-qPCR FW GCTGAGACGAACGCTTCACT REV CCTTCGAACACTGACCCACT	This paper	N/A
siRNA siSUPT7L-46 CUACUAGACCCAACAGAAA [dT] [dT] UUUCUGUUGGGUCUAGUAG[dT] [dT]	This paper	N/A
siRNA siSUPT7L-47 CUAUCACAGUUACAUGC UA[dT] [dT] UAGCAUGUAACUGUGAUAG[dT] [dT]	This paper	N/A
siRNA siKAT2B (PCAF) CUCUAAUCCUCACUCA UUU[dT] [dT] AAAUGAGUGAGGAUUAGAG[dT] [dT]	This paper	N/A
siRNA siKAT2A (GCN5) GCUACUACGUGACCC GGAA[dT] [dT] UUCCGGGUCACGUAGUAGC[dT] [dT]	This paper	N/A

**Recombinant DNA**		

pX330-LMNAgRNA1	Pauty et al., 2017	N/A
pCR2.1-CloverLMNAdonor	Pinder et al., 2015	N/A
piRFP670-N1	Pinder et al., 2015	N/A
AVS1_Puro_PGK1_3xFLAG_Twin_Strep	Dalvai et al., 2015	Addgene #68375

**Software and Algorithms**		

FastQC	N/A	https://www.bioinformatics.babraham.ac.uk/projects/fastqc
Bwa	N/A	http://bio-bwa.sourceforge.net
Samtools	N/A	http://www.htslib.org/
deepTools	N/A	http://deeptools.readthedocs.io
R	N/A	https://www.r-project.org

### Contact for Reagent and Resource Sharing

Further information and requests for resources and reagents should be directed to and will be fulfilled by the Lead Contact, Gaëlle Legube (gaelle.legube@univ-tlse3.fr). DIvA cells are subjected to an MTA, to be signed with the CNRS.

### Experimental Model and Subject Details

#### Chemicals

4-hydroxytamoxifen (4OHT) was purchased form Sigma (Sigma; H7904). 4OHT is reconstituted in DMSO at a final concentration of 10 mM, and stored at −20°C. 4OHT was further used at a final concentration of 300 nM to induce DSB in DIvA cells. Thymidine is purchased from Sigma (T1895), reconstituted in PBS at 100 mM and filter sterilized immediately before use.

#### Cell Culture and Cell Lines

DIvA (AsiSI-ER-U20S) cells were cultured in Dulbecco’s modified Eagle’s medium (DMEM) supplemented with antibiotics, 10% FCS (Invitrogen) with 1 μg/mL puromycin at 37°C under a humidified atmosphere with 5% CO2. K562 cells stably expressing near physiological levels of 3xFLAG-Twin-Strep-tagged SUPT7L (Dalvai et al., 2015, Doyon and Côté, 2016) were cultured in RPMI medium supplemented with 0.5 μg/mL puromycin.

### Method Details

#### Cell treatments

For AsiSI-dependent DSB induction, cells were treated with 300 nM 4OHT for 4 hr. For synchronization in G1 and G2, cells were incubated with 2 mM thymidine for 18 hr, released for 12 hr and subjected to the second thymidine treatment for 18 hr. G1 and G2 cells were treated with 4OHT, respectively, at 11 and 6 hr following thymidine release and harvested 4 hr later.

#### Establishment of cell line expressing SUPT7L from the AAVS1 safe harbor and TAP Purification of native human SAGA HAT/DUB complex

K562 cells stably expressing near physiological levels of 3xFLAG-Twin-Strep-tagged SUPT7L was established as described previously (Dalvai et al., 2015, Doyon and Côté, 2016). Briefly, SUPT7L cDNA was cloned into AAVS1_Puro_PGK1_3xFLAG_Twin_Strep (Addgene #68375). The cassette is integrated at the AAVS1 locus after DSB induction and recombination targeted by co-transfection with ZFN expression plasmid. Two hundred thousand cells were transfected with 400 ng of ZFN expression vector and 4 μg of donor constructs. Selection and cloning were performed in RPMI medium supplemented with 0.5 μg/mL puromycin starting at 2 to 3 days post transfection. Clones were obtained by limiting dilution and expanded before harvest for western blot analysis.

Native SAGA complex was purified from 3L of the 3xFlag-Twin-strep-tagged SUPT7L K562 cells as described in (Doyon and Côté, 2016). Nuclear extracts were prepared following standard procedures and pre-cleared with CL6B Sepharose beads. FLAG immunoprecipitations with anti-FLAG agarose affinity gel (Sigma M2) were performed followed by elution with 3xFLAG peptide (200 μg/mL from Sigma in the following buffer: 20 mM HEPES pH 7.5, 150 mM KCl, 0.1 mM EDTA, 10% glycerol, 0.1% Tween20, 1 mM DTT and supplemented with proteases, deacetylases, and phosphatase inhibitors), followed by Strep immunoprecipitation with Strep-Tactin Sepharose beads (IBA) and elution with 5 mM D-biotin in the same buffer used for Flag elution. Mass spec analysis confirmed the co-purification of all bona fide subunits of the human SAGA complex.

#### *In vitro* histone acetylation assay

Histone acetyltransferase assays were performed as described previously with minor modifications using the purified complex (Jacquet et al., 2016). Briefly, 100 ng of the recombinant H2B was incubated in a 15 μL reaction containing 50 mM Tris-HCl pH 8.0, 10% glycerol, 1 mM EDTA, 1 mM DTT, 1 mM PMSF, 10 mM sodium butyrate 0.15 mM unlabeled Acetyl-CoA (Sigma) with or without the purified complex for 30 min at 30°C. Samples were analyzed by western blot with indicated antibodies.

#### *In vitro* histone de-ubiquitination assay

DUB enzymatic activity was assayed by pre-incubating native nucleosomes purified from HeLa cells in buffer containing 50 mM Tris-HCl pH 7.5, 125 mM NaCl, 1 mM DTT, 1 mM MgCl_2_, 1 mM EDTA, and protease inhibitor cocktail at 30°C for 20 min before adding purified SAGA complex. Reaction were incubated at 30°C throughout the time course and terminated by adding SDS-PAGE sample loading buffer following by western blot analysis using indicated antibodies.

#### RT-qPCR

For measuring siRNA-mediated depletion total RNA was extracted using the Quick-RNA MicroPrep kit (Zymo Research). One microgram of RNA was reverse transcribed using qScript cDNA synthesis kit (Quanta biosciences) according to the manufacturers’ protocols. Samples were then subjected to quantitative PCR (qPCR) using Lightcycler (Roche). The relative abundance of target mRNA was calculated according to the ΔΔ cycle threshold method (ΔΔCt). mRNA expression levels of the housekeeping gene 36B4 gene (also called ribosomal phosphoprotein P0, RPLP0) were used as an internal control to normalize each qPCR reaction. The relative expression levels were calculated as fold enrichment of treated cells over the control cells. Experiments were performed as independent biological triplicates and data are presented as mean ± SD.

#### Cas9/mClover-LMNA1 homologous recombination assay

The pCR2.1-CloverLMNAdonor, pX330-LMNAgRNA1 and piRFP670-N1 plasmids were previously described (Pinder et al., 2015). The pX330-LMNAgRNA1 plasmid used in this study was modified from the original plasmid to remove the 3XFlag tag from the Cas9 endonuclease previously described (Pauty et al., 2017). U2OS cells were seeded and transfected with indicated siRNA using Lipofectamine RNAiMAX (Invitrogen). Twenty-four hours post-transfection, 1.5 million cells were transfected on the 4D-Nucleofector X-unit (program DG-130), using complete nucleofector solution (SE. Cell Line 4D-Nucleofector X Kit, Lonza) containing 1 μg of pCR2.1-CloverLMNAdonor, 1 μg pX330-LMNAgRNA1, 0.1 μg of piRFP670-N1 (used as transfection control) and 200pmol of siRNA and, immediately resuspended in culture media and transferred to a 10 cm dish. After 48 hr, cells were trypsinized and 0.25 million cells plated into glass coverslips and the rest analyzed on BD Accuri C6 Plus Flow Cytometer. Cloverexpression was assayed by fluorescence microscopy the next day that is 72 hr post-nucleofection. Data represent the mean percentages (±SD) of Clover-positive cells (structured nuclear GFP signal) over the iRFP670-positive population from independent experiments performed in triplicates (n > 800 cells per condition).

#### NHEJ assay

The U2OS cell line with I-Sce1 reporter GFP-RFP cassette to measure NHEJ has been described (Jacquet et al., 2016). The cells were transfected with 200 nmol of the indicated siRNA using Lipofectamine RNAimax (Invitrogen) for 36 hr and infected with I-Sce1 adenovirus for 1 hr. Cells are harvested 48 hr after DSB induction and analyzed by FACS for GFP and RFP expression on a BD Accuri C6 Plus Flow Cytometer. Control cells produce 10% of RFP positive/GFP negative cells in the NHEJ reporter. The data presented are from biological triplicate experiments.

#### Cell cycle analysis

For cell cycle studies, DIvA cells were harvested by trypsinization, fixed with cold 70% ethanol, treated with ribonuclease A and propidium iodide. FACS analysis was performed using a BD Accuri C6 Plus Flow Cytometer.

#### ChIP

ChIP assays were carried out according to the protocol described in Aymard et al., 2014 and Iacovoni et al., 2010. The amount of chromatin and antibodies used are detailed in Table S2. For quantitative PCR analysis (Figure S2), both input and IP samples were analyzed using the primers FW GATTGGCTATGGGTGTGGAC and REV CATCCTTGCAAACCAGTCCT (HR DSB1). For Figure S4, the following primers were also used: FW CCGCCAGAAAGTTTCCTAGA and REV CTCACCCTTGCAGCACTTG (HR-DSB2), FW TGCCGGTCTCCTAGAAGTTG and REV GCGCTTGATTTCCCTGAGT (NHEJ-DSB), FW AGCCGGGCTCTTGCCAAT and REV AGTTAGCGCCCAAAGGACCA (*ACTB*), FW GCTGAGACGAACGCTTCACT and REV CCTTCGAACACTGACCCACT (*TAF12*). ChIP efficiencies were calculated as the percent of input DNA immunoprecipitated. Prior to next-generation sequencing library preparation, samples from multiple ChIP experiments were pooled and sonicated for 5 cycles (30 s on, 30 s off, high setting) with a Bioruptor (Diagenode) then concentrated with a vacuum concentrator (Eppendorf). Sequencing libraries were prepared by using 10 ng of purified DNA (averaged size 250-300 bp), and subjected to high throughput sequencing.

#### BLESS

BLESS was performed as described in Crosetto et al., 2013. Briefly, cells were fixed with 2% formaldehyde to stabilize chromatin and prevent artificial DSBs, plasma membranes were lysed, and intact nuclei were recovered by centrifugation. Nuclei were deproteinated using Proteinase K. DSBs were then blunted and 5′phosphorylated using the Quick Blunting Kit (NEB) and ligated to a biotinylated linker (proximal) using T4 ligase (NEB). Total DNA was extracted by precipitation with isopropanol and fragmented by sonication using a Covaris S220 to create ∼400 bp fragments. Labeled fragments were captured by streptavidin beads (Invitrogen) and once again blunted and phosphorylated using the Quick Blunting Kit (NEB), and ligated to a second linker (distal). The resulting circular DNA was linearized by I-SceI (NEB) digestion and amplified by PCR. Sequencing libraries were prepared using TruSeq Nano DNA LT Library Preparation Kit (Illumina). Quality and quantity of libraries were assessed on 2100 Bioanalyzer (Agilent) using High Sensitivity DNA Kit (Agilent), and on Qubit 2.0 using Qubit dsDNA HS Assay Kit (ThermoFisher).

### Quantification and Statistical Analysis

#### ChIP-seq data processing

H3K4me2, H4K20me1, H3K36me3, H3K79me2, H2Bub, H3K56ac, H4K16ac, H3K9me2, H4S1P, H3K36me2, and γH2AX samples were sequenced using Illumina HiSeq 2500 (single-end, 50 bp reads) at BGI (Beijing Genomics Institute, Hong Kong). FK2 samples were sequenced using Illumina HiSeq 2500 (single-end, 50 bp reads) at GATC biotech (Konstanz, Germany). XRCC4 at 1, 4, and 24 hr post 4OHT treatment, DNA ligase IV, H2BK120ac, H2AZ, H1, MacroH2A, H3K9me3, H4K20me2, 53BP1, and γH2AX G1 and G2 samples were sequenced using Illumina NextSeq 500 (single-end, 75 bp reads) at EMBL Genomics core facilities (Heidelberg, Germany). H4K12ac, H2AZac, and H3 were sequenced using Illumina HiSeq 2500 (single-end, 50 bp reads) at EMBL Genomics core facilities (Heidelberg, Germany). Previously published data include RAD51 and XRCC4 ChIP-seq in OHT-treated DIvA cells (Aymard et al., 2014), RNA Polymerase II (S2P) in untreated DIvA cells (Cohen et al., 2018) and MethylCap-Seq data in U2OS cells (Deplus et al., 2014)

The quality of each raw sequencing file (fastq) was verified with FastQC (https://www.bioinformatics.babraham.ac.uk/projects/fastqc/). All files were aligned to the reference human genome (hg19) and processed using a classical ChIP-seq pipeline: bwa (http://bio-bwa.sourceforge.net/) for mapping and samtools (http://www.htslib.org/) for duplicate removal (rmdup), sorting (sort) and indexing (index). Coverage for each aligned ChIP-seq dataset (.bam) were computed with the rtracklayer R package and normalized using total read count for each sample. Coverage data were exported as bigwig (file format) for further processing

#### BLESS data processing

Samples were sequenced with HiSeq 2500 (Illumina) according to our custom protocol for low-diversity samples (Mitra et al., 2015), generating 61-bp paired-end reads containing BLESS barcodes (distal and proximal). Both barcodes were removed using cutadapt, and BLESS data were aligned on hg19 using bwa in paired-end mode (bwa aln and bwa sampe). In order to prevent reads that represent bona fide DSB signal, but start exactly at cleaved AsiSI sites from being marked as duplicates and improperly discarded, fragments were reconstituted from paired reads using Rsamtools and GenomicAlignments (R packages). Fragments with lengths > 500 bp were dropped as aberrant and the remaining fragments were de-duplicated (fragments with exact same start and end position on genome were considered as duplicates and only kept once). To determine which sites among the 1211 AsiSI sites were indeed cleaved, we used the total count for BLESS signal in a 1 kb windows around all AsiSI sites positions on the genome. Outliers (values > third quartile + 1.5 x interquartile range) were considered to be cleaved. Of the 174 sites defined as outliers, we only focused on 80 DSBs (showing the highest BLESS signal) that were significantly induced (Figures 1B–1D, Table S1).

#### Descriptive statistics using box-plot representation

Each box-plot representation was generated with R-base. The center line represents the median, box ends represent respectively the first and third quartiles, and whiskers represent the minimum and maximum values without outliers. Outliers were defined as first quartile − (1.5 × interquartile range) and above third quartile + (1.5 × interquartile range).

#### Statistical analysis and representations

Statistical hypothesis testing was performed using nonparametric unpaired Mann-Whitney-Wilcoxon (wilcoxon.test() function in R) to tests distribution differences between two populations. For damaged versus undamaged chromatin features comparisons, boxplots represent the ChIP-seq enrichment ratio between 4OHT treated and untreated DIvA cells (expressed as a log2 ratio) for DSBs or control regions. Significant differences were determined using two-sample Wilcoxon tests. Significant increases are colored in red or orange (for p value < 0.01 or p value < 0.05, respectively), significant decreases in dark or light blue (for p value < 0.01 or p value < 0.05, respectively) and non-significant differences (ns, p value > 0.05) in gray.

In the circle plot representation (Figures 4F and S5E), each circle radius represents the p value of a nonparametric two-sided Mann-Whitney-Wilcoxon test comparing ChIP-seq counts in a given window size. In Figures 4E and S5D, total ChIP-seq count for each dataset (untreated condition) was compared for HR and NHEJ DSBs. Individual modifications were determined as enriched in HR or NHEJ using one-sided Mann-Whitney-Wilcoxon tests (if p value < 0.05). In Figure S5E, total ChIP-seq count for each dataset was compared in treated and untreated conditions, separately for HR or NHEJ DSBs. Individual modifications were determined as increased or decreased following DSB induction using one-sided Mann-Whitney-Wilcoxon tests (p value < 0.05). In Figure S6B, Spearman correlation coefficients were determined using the cor() function in R and the correlation matrix was generated using the corrplot package. For Figure S4B, to check if DSBs repaired by HR or NHEJ exhibit a non-random distribution among the main 3D genomic compartments (A1, A2, B1, B2, B3, B4 and NA (non-assigned)) (Rao et al., 2014)), we compared the distribution of all genomic loops among these compartments with the distributions for loops containing either HR-repaired or NHEJ-repaired DSBs (no loop contained both HR and NHEJ sites). The p values were calculated using hypergeometric probability distribution, with the null hypothesis that HR and NHEJ loops are distributed among the 3D compartments in the same manner as all genomic loops. Conservative Bonferroni correction for multiple hypothesis testing was applied to correct p values to reflect testing all 3D compartments for both possible enrichments and depletions.

#### Averaged ChIP-seq profiles

Averaged ChIP-seq profiles were generated using the R package ggplot2. The x axis represents genomic position relative to DSB and the y axis represents the mean coverage at each bp, except for larger windows (1 Mb scale) where data were smoothed using a 50 kb span. Log2 ratio was computed using the bamCompare tool from deepTools (http://deeptools.readthedocs.io) with two bam files (before and after damage) as inputs. Positive and negative values for log2 ratio are respectively represented in red and blue. For γH2AX, 53BP1, RAD51, and XRCC4, ChIP-seq data are only available for damaged cells. In this case, the y axis only represents the mean ChIP-seq coverage in the damaged condition.

#### Generation of random positions

In order to generate a control set of non-DSB regions, we first computed a thousand random positions on the entire genome (excluding chromosome Y) using R. These random sites were filtered for being at least 1 Mb away from the top 150 cleaved positions with IRanges::findOverlaps() function. We also excluded regions with a null ChIP-seq count (in any of our datasets) in a 1 kb window in order to avoid regions that are systematically underrepresented in ChIP-seq experiments. Finally, 80 sites were randomly picked from the remaining list.

#### Determination of HR-prone and NHEJ-prone DSBs

We computed a ChIP-seq coverage ratio between RAD51 (4 kb window) and XRCC4 (1 kb window) for each of the 80 induced DSBs. Sites with the highest ratio were designated as HR-prone and sites with the lowest ratio as NHEJ-prone (30 DSBs in each category). For the analysis performed Figure S5D, HR-prone and NHEJ–prone DSBs were obtained by computing the ratio between RAD51 (4 kb window) and Ligase IV (1 kb window) for each of the 80 induced DSBs and selecting sites with highest and lowest ratios (30 DSBs in each category).

### Data and Software Availability

The accession number for the high-throughput sequencing data reported in this paper is ArrayExpress: E-MTAB-5817.

Source code for generating boxplots, heatmaps, and average profiles is available on the GitHub repository page: https://github.com/LegubeDNAREPAIR/HistoneMapping.

## Supplemental Information

Document S1. Figures S1–S7 and Tables S1 and S2

Document S2. Article plus Supplemental Information
