# Supplementary material for: Comprehensive Mapping of Histone Modifications at DNA Double-Strand Breaks Deciphers Repair Pathway Chromatin Signatures
Source: Mol Cell. 2018 Oct 18;72(2):250–262.e6. doi: 10.1016/j.molcel.2018.08.020 (PMC6202423; doi:10.1016/j.molcel.2018.08.020)
Supplement: Document S1. Figures S1–S7 and Tables S1 and S2 [file mmc1.pdf]

Molecular Cell, Volume *72*

Supplemental Information

Comprehensive Mapping of Histone Modifications at DNA Double-Strand Breaks Deciphers Repair Pathway Chromatin Signatures

Thomas Clouaire, Vincent Rocher, Anahita Lashgari, Coline Arnould, Marion Aguirrebengoa, Anna Biernacka, Magdalena Skrzypczak, François Aymard, Bernard Fongang, Norbert Dojer, Jason S. Iacovoni, Maga Rowicka, Krzysztof Ginalski, Jacques Côté, and Gaëlle Legube

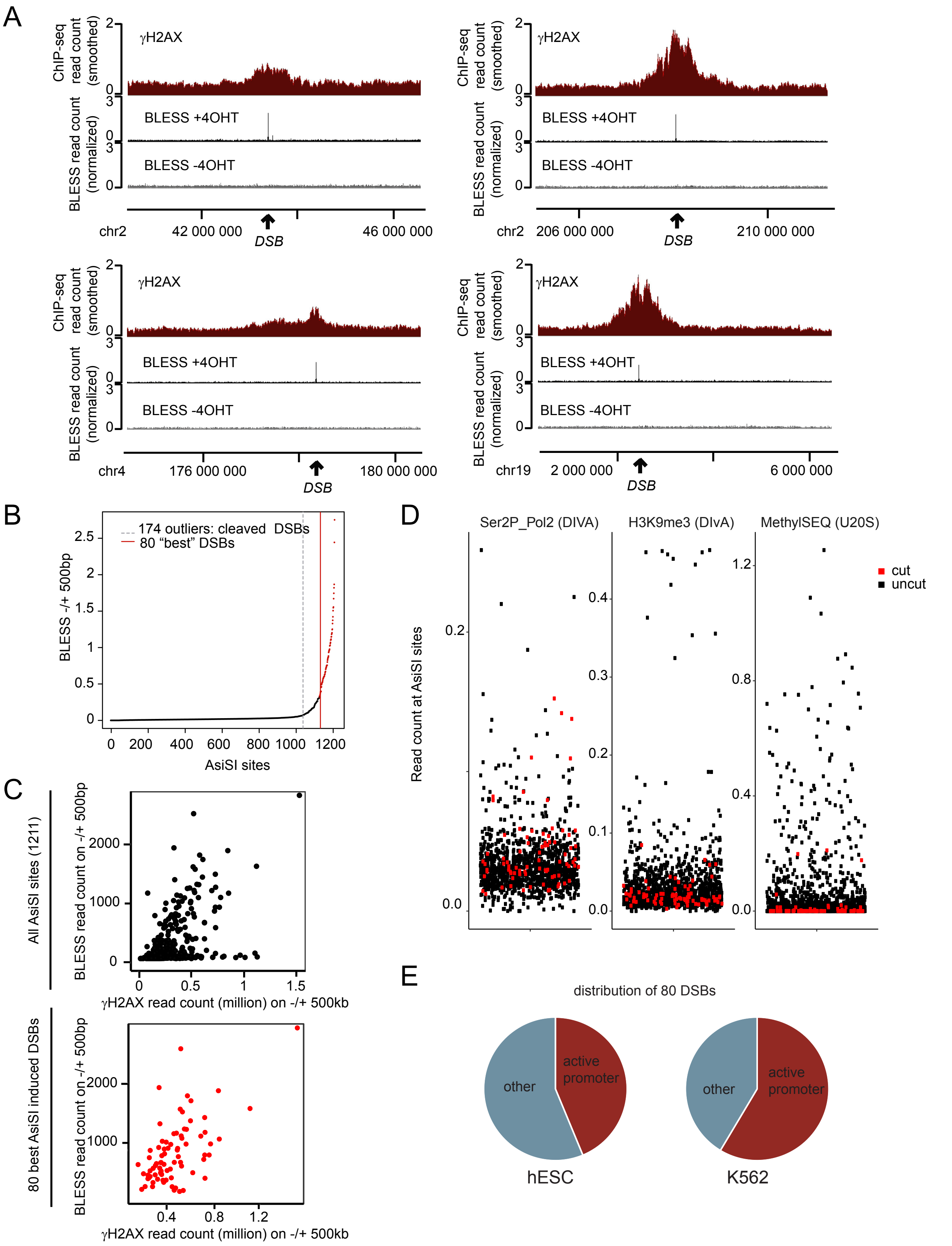
A
ChIP-seq read count (smoothed)
γH2AX
BLESS read count (normalized)
BLESS +4OHT
BLESS -4OHT
chr2
42 000 000
46 000 000
DSB
chr2
206 000 000
210 000 000
DSB
chr4
176 000 000
180 000 000
DSB
chr19
2 000 000
6 000 000
DSB
B
174 outliers: cleaved DSBs
80 "best" DSBs
BLESS -/+ 500bp
AsiSI sites
C
All AsiSI sites (1211)
BLESS read count on -/+ 500bp
γH2AX read count (million) on -/+ 500kb
80 best AsiSI induced DSBs
BLESS read count on -/+ 500bp
γH2AX read count (million) on -/+ 500kb
D
Ser2P_Pol2 (DIVA)
H3K9me3 (DIvA)
MethylSEQ (U20S)
Read count at AsiSI sites
cut
uncut
E
distribution of 80 DSBs
other
active promoter
hESC
other
active promoter
K562

Figure S1. related to Figure 1: AsiSI-induced DSB mapping by BLESS

(**A**) Genome browser screenshots representing γH2AX ChIP-seq (4OHT treated cells) and BLESS (4OHT untreated or treated cells) signal at DSBs located on chromosome 2, 4 and 19 as indicated.
(**B**) Dotplot representing BLESS read count in a 1 kb window for the 1211 predicted AsiSI sites in the human genome. Sites are sorted by increasing signal.
(**C**) Scatterplot representing read count (from 4OHT treated cells) for γH2AX ChIP-seq (in a 1 Mb window) and BLESS (in a 1 kb window) for the 1211 predicted AsiSI sites in the human genome (upper panel) and the 80 most cleaved AsiSI sites (lower panel).
(**D**) Dotplot representing MethylCap-seq read count obtained in U20S (Deplus et al., 2014), on a 200bp window (right panel), H3K9me3 ChIP-seq read count on a 10kb window (middle panel) for and RNA PolII-S2P ChIP-seq (Cohen et al., 2018) read count in a 10 kb window (left panel) for each of the 1211 predicted AsiSI sites in the human genome. Cut sites are indicated in red.
(**E**) The 80 DSBs were compared with the chromatin state segmentation track from hESC and K562 cells (Broad ChromHMM, http://rohsdb.cmb.usc.edu/GBshape/cgi-bin/hgTrackUi?db=hg19&g=wgEncodeBroadHmm). The proportion of DSBs lying within active promoters (dark red) or other loci (grey) are shown.

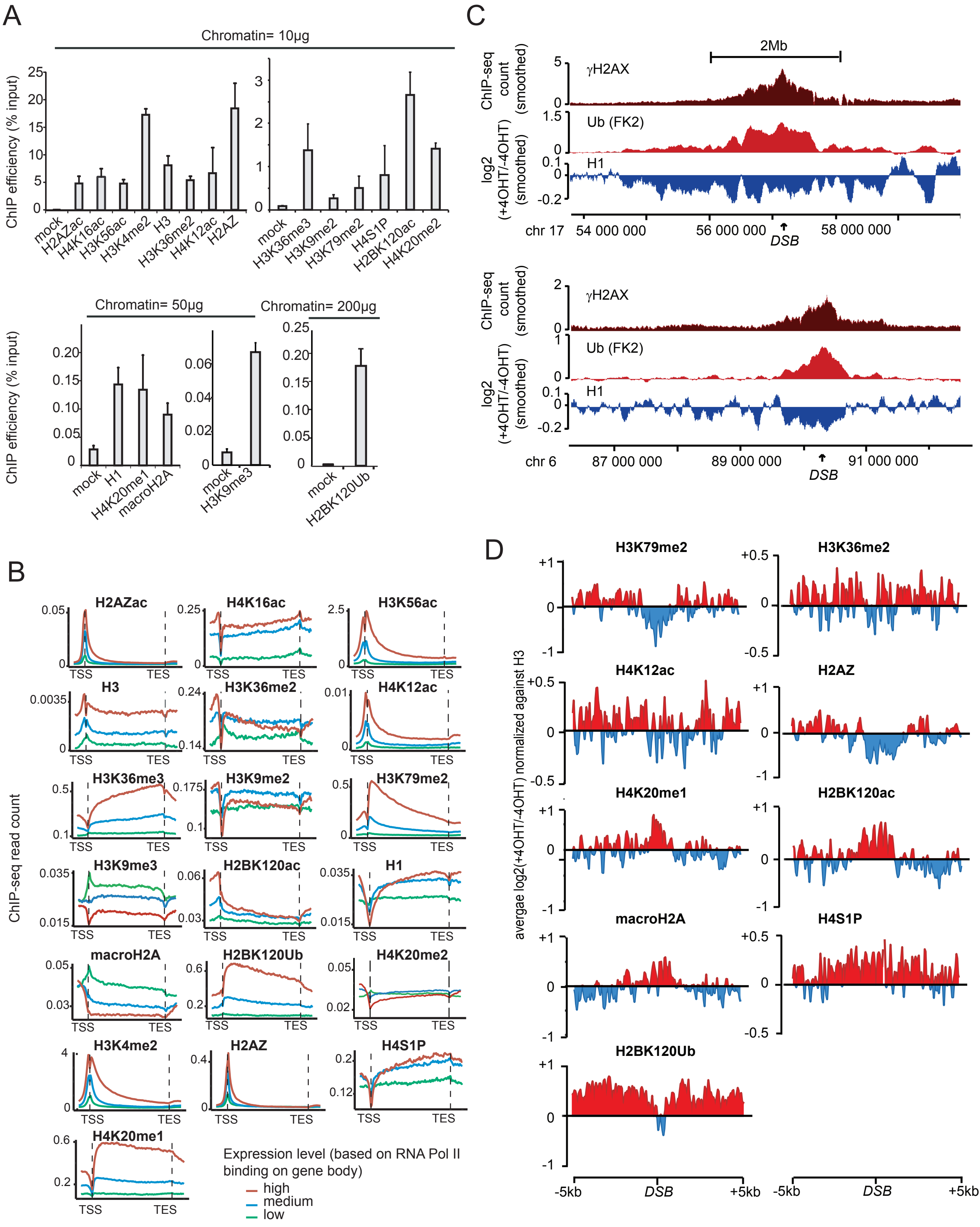
A
Chromatin= 10µg
ChIP efficiency (% input)
mock
H2AZac
H4K16ac
H3K56ac
H3K4me2
H3
H3K36me2
H4K12ac
H2AZ
H3K36me3
H3K9me2
H3K79me2
H4S1P
H2BK120ac
H4K20me2
Chromatin= 50µg
Chromatin= 200µg
H1
H4K20me1
macroH2A
H3K9me3
H2BK120Ub
B
ChIP-seq read count
TSS
TES
Expression level (based on RNA Pol II binding on gene body)
high
medium
low
C
2Mb
ChIP-seq count (smoothed)
log2 (+4OHT/-4OHT) (smoothed)
γH2AX
Ub (FK2)
H1
chr 17
54 000 000
56 000 000
58 000 000
DSB
chr 6
87 000 000
89 000 000
91 000 000
D
avergae log2(+4OHT/-4OHT) normalized against H3
H3K79me2
H3K36me2
H4K12ac
H2AZ
H4K20me1
H2BK120ac
macroH2A
H4S1P
H2BK120Ub
-5kb
DSB
+5kb

Figure S2, related to Figure 2 and 3: ChIP-seq validation and histone modification changes following AsiSI induction in DIvA cells

(**A**) ChIP were performed in untreated DIvA cells with all indicated antibodies and qPCR was performed to assess enrichment at a specific genomic location (chr1: 89458701, hg19), an AsiSI cut site. Average and SEM of at least 3 independent experiments are shown.
(**B**) Average profile for each histone modification obtained in untreated samples over human genes sorted by expression level (high in red, medium in blue, low in red). This recapitulate previous findings (Barski et al., 2007; Chen et al., 2012; Gamble et al., 2010; Gatta et al., 2011; Jung et al., 2012; Krishnakumar et al., 2008; Kuo et al., 2011; Lo et al., 2011; Millan-Arino et al., 2014; Nelson et al., 2016; Tolstorukov et al., 2012; Vakoc et al., 2006; Valdes-Mora et al., 2012; Wang et al., 2013; Wang et al., 2008). See also Table S2 for additional references.
(**C**) Genome Browser screenshots representing ChIP-seq signals for γH2AX, ubiquitin, and H1 at 2 DSBs located on chromosome 17 and 6 respectively. Data are expressed as read count (from 4OHT treated samples) for γH2AXand as a log2 ratio between 4OHT treated and untreated DIvA cells for ubiquitin (FK2) and H1, smoothed using a 100kb span.
(**D**) Average profile on a 10 kb window of the H3 normalized enrichment between 4OHT treated and untreated DIvA cells for the nine histone modifications that exhibited significant changes over 80 DSBs (Figure 3). Values are expressed as log2 ratios. Positive and negative values for log2 ratio are respectively represented in red and blue.

Clouaire, Figure S3

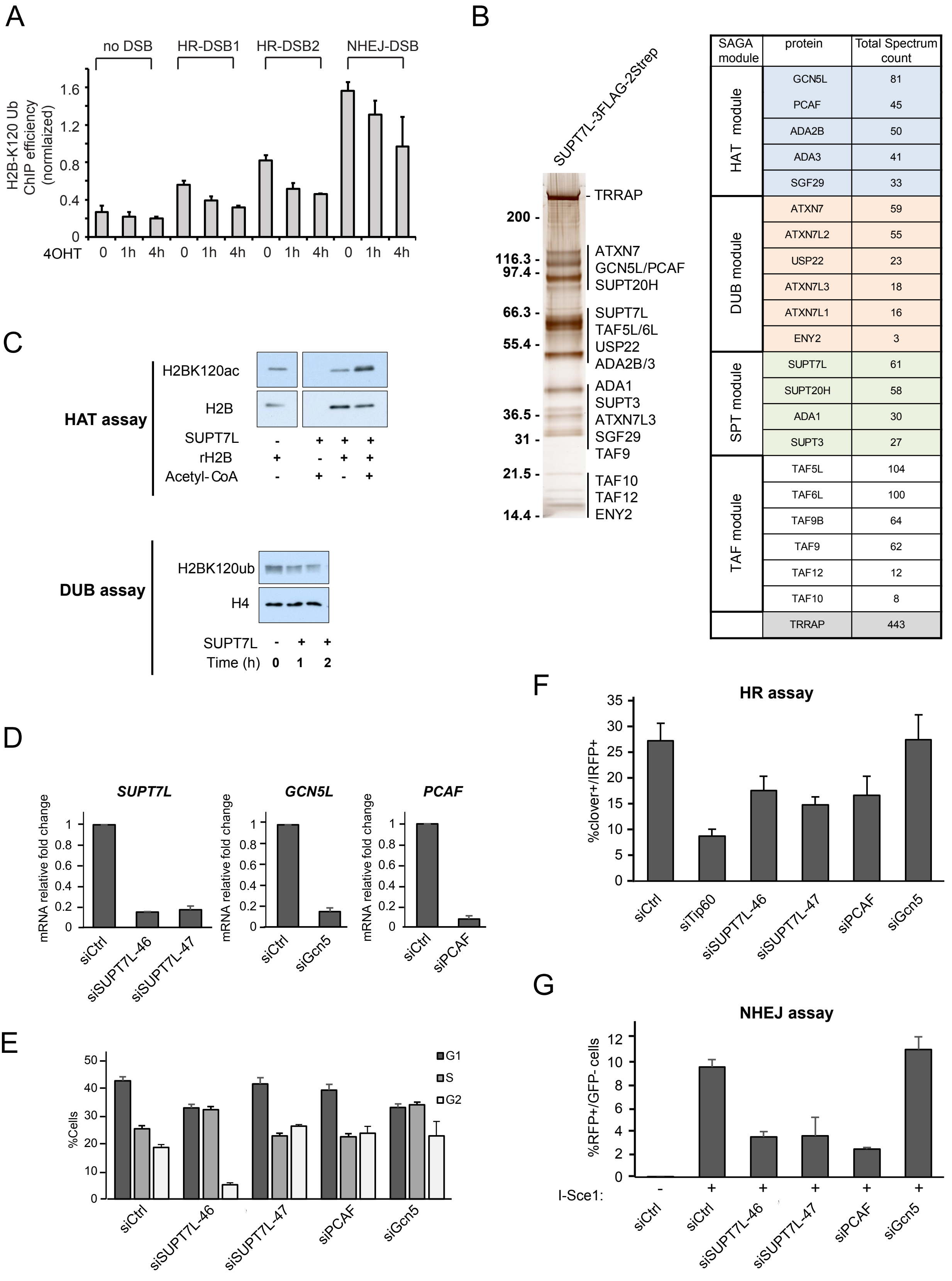

SAGA module	protein	Total Spectrum count
HAT module	GCN5L	81
	PCAF	45
	ADA2B	50
	ADA3	41
	SGF29	33
DUB module	ATXN7	59
	ATXN7L2	55
	USP22	23
	ATXN7L3	18
	ATXN7L1	16
	ENY2	3
SPT module	SUPT7L	61
	SUPT20H	58
	ADA1	30
	SUPT3	27
TAF module	TAF5L	104
	TAF6L	100
	TAF9B	64
	TAF9	62
	TAF12	12
	TAF10	8
	TRRAP	443

Figure S3, related to Figure 3: hSAGA can catalyze *in vitro* H2BK120 acetylation and deubiquitination, and contributes to DSB repair

(**A**) ChIP against H2BK120 Ubiquitination was performed in DIvA cells, either left untreated or treated with 4OHT for 1h or 4h as indicated. Enrichment was measured at a control locus for normalization (*TAF12*) and at an uncut genomic location (*ACTB*) as well as 3 AsiSI-induced DSBs (average ± SEM, n=2).

(**B**) Purification of native human SAGA complex from K562 cells. Cells expressing SUPT7L-3Flag-2Strep from the AAVS1 safe harbor were engineered and used for tandem affinity purification from nuclear extracts. The purified fraction was analyzed on gel (silver stained) and by tandem mass spectrometry to confirm purity and the copurification of all known SAGA subunits (and paralogs). Total spectral counts obtained for each subunit are presented in the table and are grouped by functional modules within the complex.

(**C**) (Top panel) In vitro histone acetyltransferase assay with purified SAGA complex with histone H2B. H2BK120 acetylation is measured by western blot analysis using total H2B signal as control. (Bottom panel) In vitro deubiquitinase assay with purified SAGA complex and human native nucleosomes. H2BK120 deubiquitination is measured by western blot using total H4 signal as a control.

(**D**) RT-qPCR showing the mRNA levels of SUPT7L, GCN5L and PCAF in cells before and after knockdown using indicated siRNAs. The mean of 3 independent experiments ± SD is shown.

(**E**) Cell cycle distributions of U2OS cells following transfection with the indicated siRNA for 72h. The mean of 2 independent experiments ± SEM is shown.

(**F**) Effect of SUPT7L, PCAF and GCN5L knockdown on DSB repair by HR using a Cas9/mClover-LMNA1 homologous recombination assay. GFP+(mClover)/iRFP+ cells were measured by FACS and the structured nuclear GFP signal linked to LMNA1 was confirmed by microscopy. Results represent the percentage of GFP+/iRFP+ cells from 3 independent experiments (average ± SD).

(**G**) Effect of SUPT7L, PCAF and GCN5L knockdown on DSB repair by NHEJ. Measurement of I-Sce1 DSB repair by non-homologous end joining in U2OS cells using an integrated PC222/GFP-RFP reporter. Cells were transfected with the indicated siRNAs for 36h, infected with I-Sce1 adenovirus to induce DSB and then assessed 48h later by FACS analysis for RFP and GFP expression. Results represent the percentage of cells that are RFP positive but GFP negative, from 3 independent experiments (average ± SD).

Clouaire, Figure S4

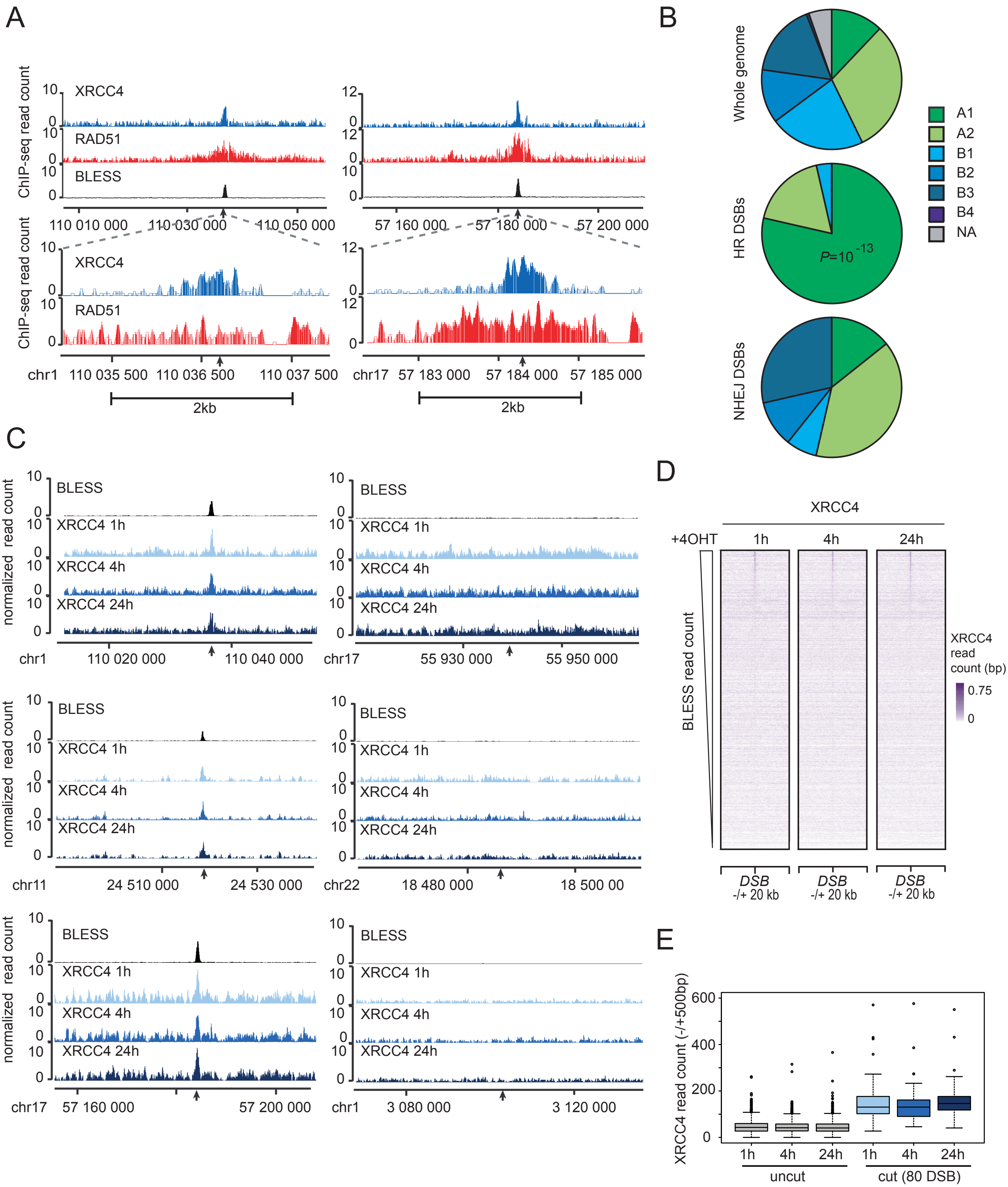

Figure S4, related to Figure 4: Identification HR and NHEJ-prone DSBs

(**A**) Genome Browser screenshots representing read counts in 4OHT treated cells for XRCC4, RAD51 and BLESS signal at two DSBs located on chromosome 1 (left) and 17 (right). Close ups are presented on bottom panels.
(**B**) Pie chart representing the distribution of loops within the different nuclear compartments (A1, A2, B1, B2, B3, B4 and NA (Rao et al., 2014)). Left panel shows distribution for all loops identified across the genome (Rao et al., 2014) , middle panel for loops containing HR-prone DSBs, and right panel for loops containing NHEJ-prone DSBs. HR-prone DSBs are very significantly enriched ($P=10^{-13}$, hypergeometric test) in the nuclear A1 compartment.
(**C**) Genome Browser screenshots representing XRCC4 ChIP-seq read counts after 1h, 4h or 24h following 4OHT addition at three DSBs exhibiting BLESS signal (cut, left panels) or no BLESS signal (uncut, right panel)
(**D**) Heatmaps representing the XRCC4 signals on a 40kb window centered around all AsiSI sites, ordered based on the BLESS level, at 1h, 4h and 24h following 4OHT treatment.
(**E**) Box plot showing XRCC4 signal on a 1kb window surrounding uncut or cut DSBs, following 1h, 4h and 24h 4OHT treatment as indicated.

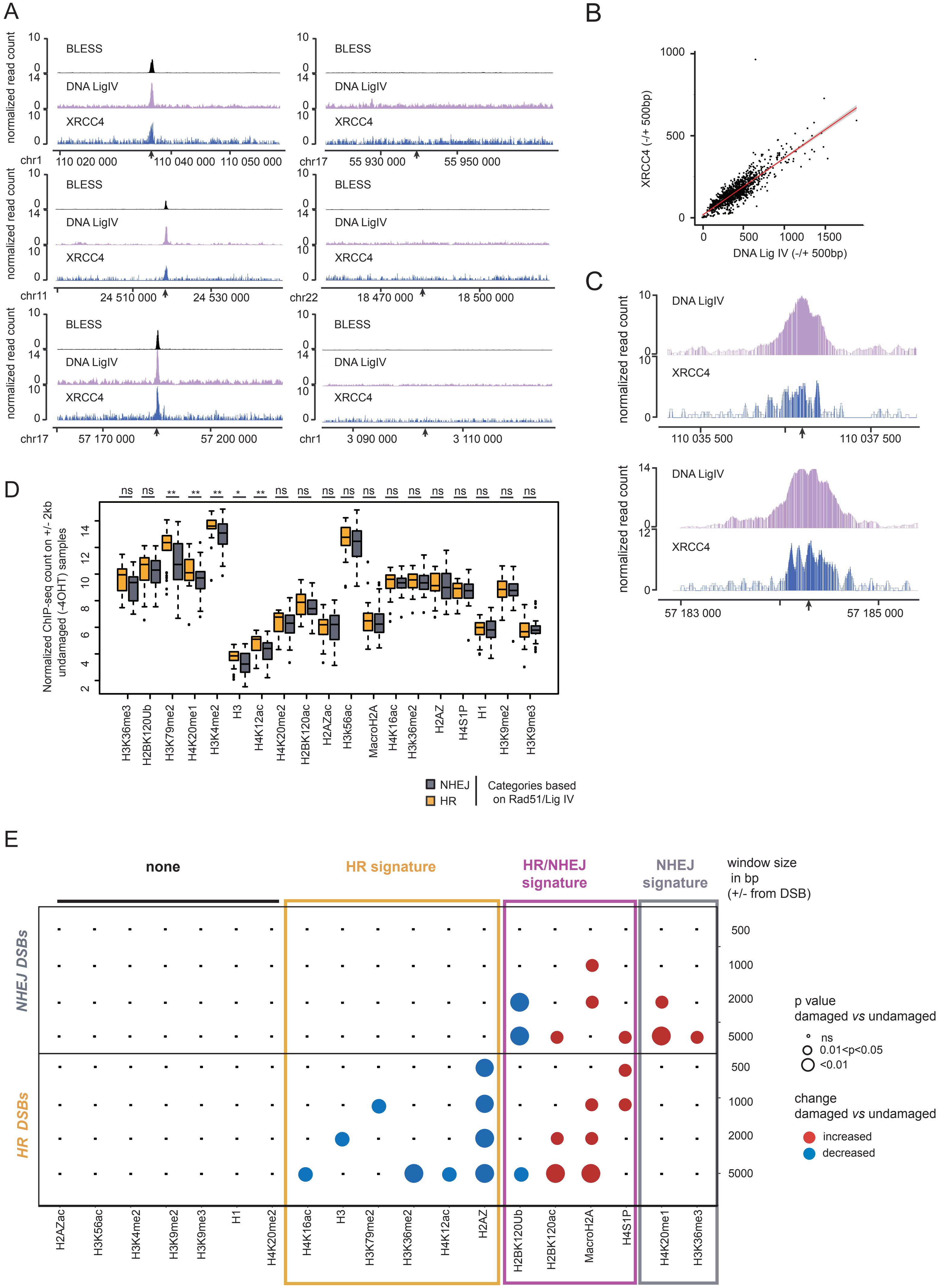
A
B
C
D
E
BLESS
DNA LigIV
XRCC4
normalized read count
chr1
110 020 000
110 040 000
110 050 000
chr17
55 930 000
55 950 000
chr11
24 510 000
24 530 000
chr22
18 470 000
18 500 000
57 170 000
57 200 000
3 090 000
3 110 000
XRCC4 (-/+ 500bp)
DNA Lig IV (-/+ 500bp)
110 035 500
110 037 500
57 183 000
57 185 000
Normalized ChIP-seq count on +/- 2kb undamaged (-4OHT) samples
H3K36me3
H2BK120Ub
H3K79me2
H4K20me1
H3K4me2
H3
H4K12ac
H4K20me2
H2BK120ac
H2AZac
H3K56ac
MacroH2A
H4K16ac
H3K36me2
H2AZ
H4S1P
H1
H3K9me2
H3K9me3
NHEJ
HR
Categories based on Rad51/Lig IV
none
HR signature
HR/NHEJ signature
NHEJ signature
window size in bp (+/- from DSB)
NHEJ DSBs
HR DSBs
p value damaged vs undamaged
ns
0.01<p<0.05
<0.01
change damaged vs undamaged
increased
decreased

Figure S5, related to Figure 4 and 5: Use of DNA Lig IV to confirm HR and NHEJ-prone DSBs and characterization of HR and NHEJ histone signature

(**A**) Genome Browser screenshots representing XRCC4 and DNA ligase IV ChIP-seq read counts after 4h following 4OHT addition at three DSBs exhibiting BLESS signal (cut, left panels) or no BLESS signal (uncut, right panel). Regions are the same as in Figure S4C.
(**B**) Scatterplot showing the level of DNA ligase IV (x axis) and XRCC4 (y axis) on a 1kb window around each annotated AsiSI sites
(**C**) Genome Browser screenshots (close up) representing read counts in 4OHT treated cells for XRCC4 and DNA Ligase IV at two DSBs induced by AsiSI
(**D**) Boxplot representing the ChIP-seq read count in a 4 kb window for each histone modification in untreated cells for 30 HR (yellow) and 30 NHEJ (grey) DSBs determined using Rad51/DNA ligase IV ratio instead of Rad51/XRCC4. P-values were calculated using two-sample Wilcoxon test. $^*P < 0.05$, $^{**}P < 0.01$; $P > 0.05$ is not significant (ns).
(**E**) Circle plot analysis showing significant changes observed between 4OHT treated and untreated DIvA cells at HR-prone (bottom) and NHEJ prone (top) DSBs, using increasing window size. Radius size represents P-values (from two-sample Wilcoxon test) when comparing ChIP-seq signal for treated and untreated samples. Significant increases (+4OHT>-4OHT) are colored in red, while significant decreases (+4OHT<-4OHT) are colored in blue. Histone modifications that undergo significant changes only at HR-DSBs are squared in yellow, those that change at both HR and NHEJ in purple and those specific for NHEJ in grey.

Clouaire, Figure S6

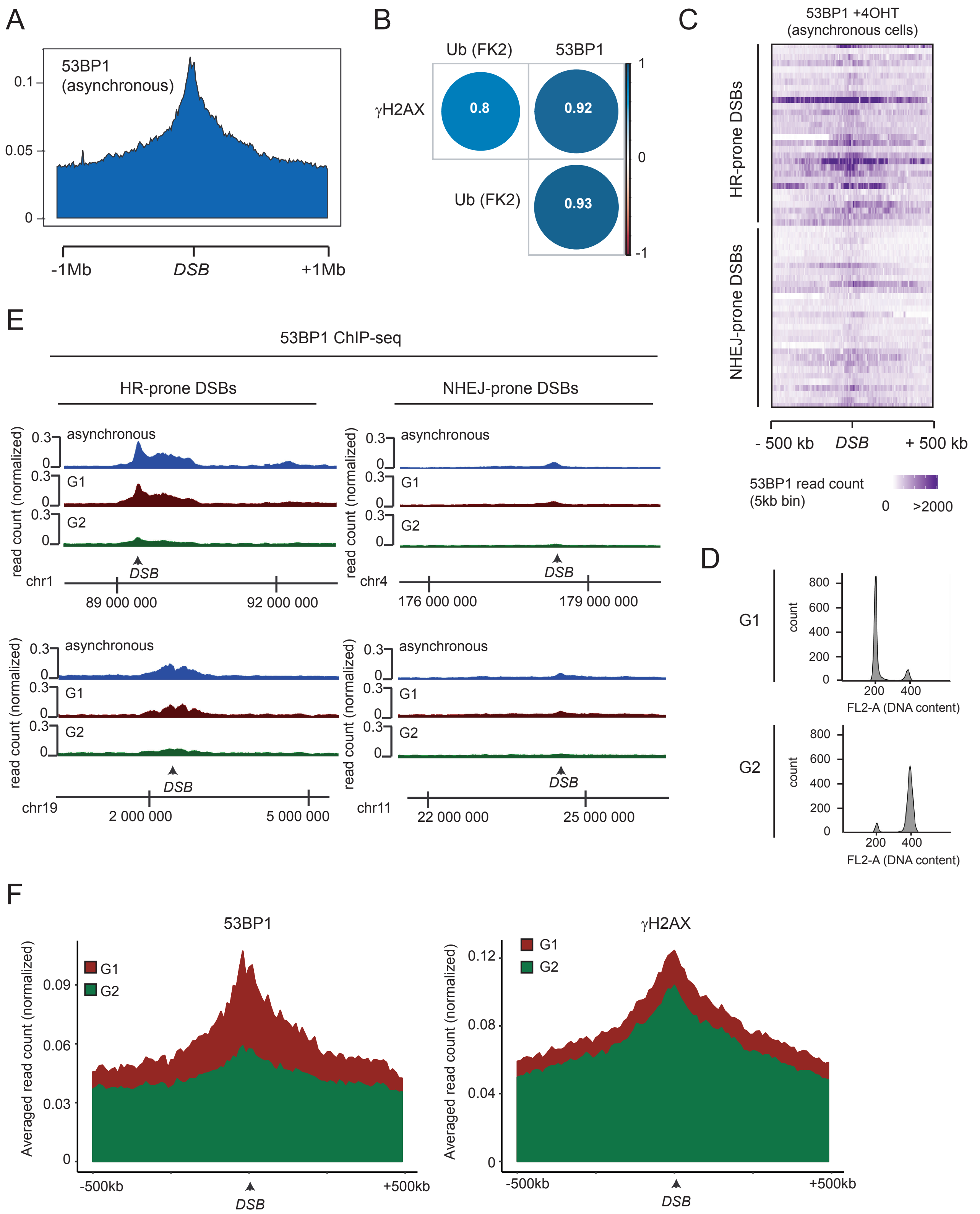

Figure S6, related to Figure 7: 53BP1 distribution analyzed by ChIP-seq

(**A**) Average profile for 53BP1 ChIP-seq (read count from 4OHT treated cells) for 80 DSBs in a 2 Mb window.
(**B**) Spearman correlation matrix of ChIP-seq read count (from 4OHT treated cells) for γH2AX, ubiquitin (FK2) and 53BP1 for 80 DSBs in a 1 Mb window.
(**C**) Heatmap representing the 53BP1 signal on a 1Mb window centered around 30 HR (top part) and 30 NHEJ sites (bottom part).
(**D**) FACS profiles indicating the cell cycle distribution for G1- and G2- 53BP1 and γH2AX ChIP-seq.
(**E**) Genome Browser screenshots representing 53BP1 ChIP-seq signals (from 4OHT treated samples) in asynchronous, G1 or G2 synchronized DIvA cells for 2HR and 2 NHEJ-prone DSBs.
(**F**) Average profiles for 53BP1 (left) and γH2AX (right) ChIP-seq in G1 (red) and G2 (green) synchronized cells (read count from 4OHT treated cells) for 80 DSBs in a 1 Mb window.

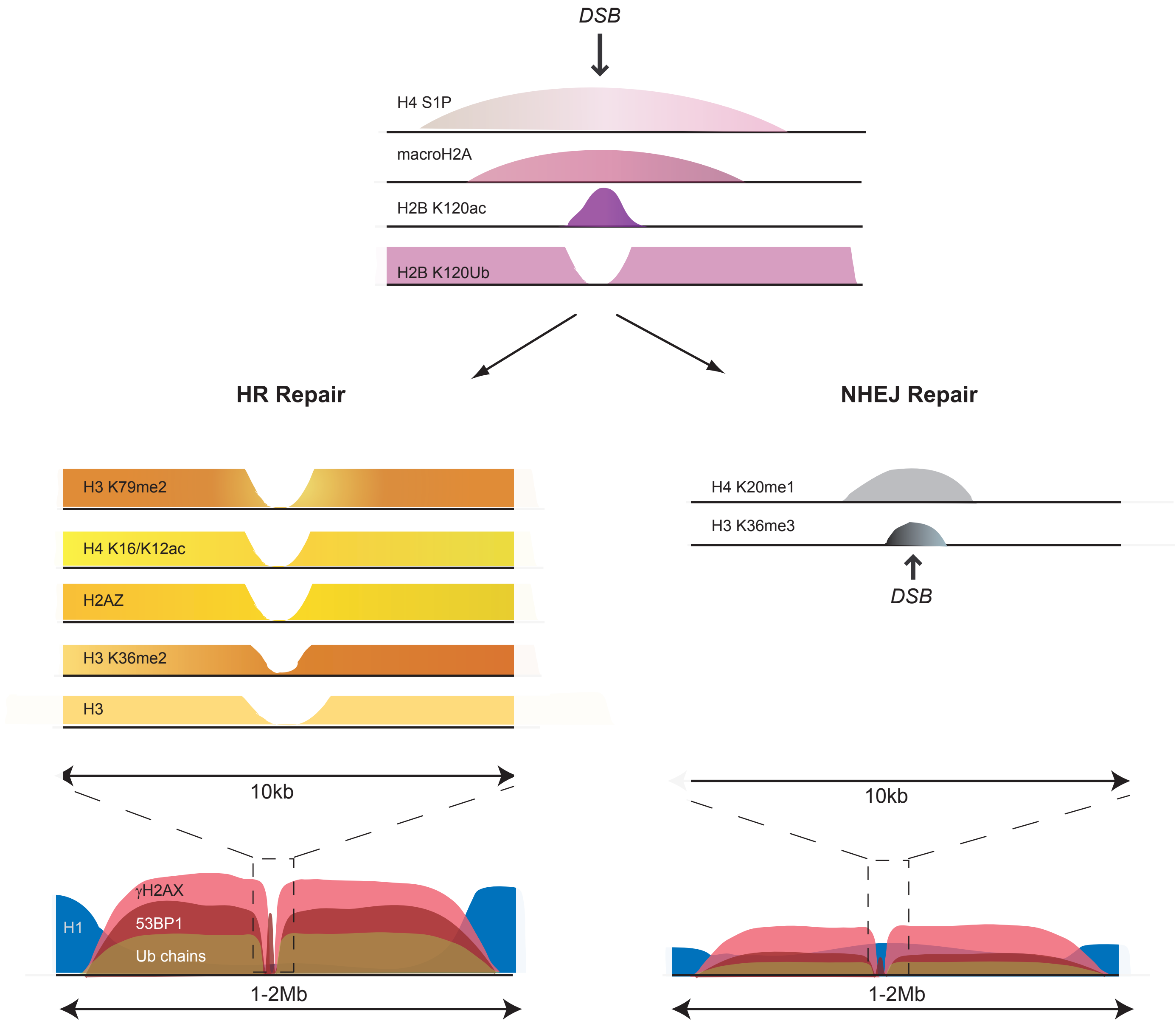
DSB
H4 S1P
macroH2A
H2B K120ac
H2B K120Ub
HR Repair
NHEJ Repair
H3 K79me2
H4 K16/K12ac
H2AZ
H3 K36me2
H3
10kb
γH2AX
H1
53BP1
Ub chains
1-2Mb
H4 K20me1
H3 K36me3
DSB
10kb
1-2Mb

Figure S7, related to Figure 2-7: **Summary for DSB-induced chromatin changes**

Following DSB induction, macroH2A is deposited, H4 is phosphorylated on Serine 1 and H2B undergoes a switch from ubiquitination to acetylation on lysine 120. At DSBs repaired by NHEJ, this is accompanied by an increase of H4 monomethylation on lysine 20 and H3 trimethylation on lysine 36. At DSB repaired by HR, which mainly reside in transcriptionally active chromatin, these chromatin changes are also associated with the demethylation of H3K79me2, deacetylation of H4 and H2AZ removal, all previously known to crosstalk with H2BK120 monoubiquitination. HR-prone DSBs also experience an acute, large-scale chromatin signaling with accumulation of γH2AX and ubiquitin conjugates, depletion of histone H1, and 53BP1 binding. While γH2AX signaling occurs at all cell cycle phase, 53BP1 mainly accumulates at HR-prone DSBs during G1. Such modifications on the megabase scale likely alters chromatin fiber properties to be translated into changes in chromatin mobility within the nucleus. This could potentially favor homology search and/or clustering, features of HR-prone DSBs.

chromosome	start	end
chr1	9649446	9649452
chr1	40974644	40974650
chr1	89458597	89458603
chr1	110036700	110036706
chr1	204380453	204380459
chr1	224032648	224032654
chr2	43358339	43358345
chr2	55509101	55509107
chr2	68384749	68384755
chr2	74734762	74734768
chr2	85822594	85822600
chr2	120124566	120124572
chr2	208030728	208030734
chr3	52232163	52232169
chr3	98618165	98618171
chr3	99536965	99536971
chr4	83934287	83934293
chr4	178363576	178363582
chr5	68462851	68462857
chr5	79784140	79784146
chr5	142785050	142785056
chr6	27145367	27145373
chr6	31105428	31105434
chr6	37321812	37321818
chr6	49917583	49917589
chr6	67704021	67704027
chr6	90348187	90348193
chr6	135819348	135819354
chr6	144607569	144607575
chr6	149888106	149888112
chr7	75807507	75807513
chr7	92861491	92861497
chr7	99679508	99679514
chr8	66546348	66546354
chr8	116680632	116680638
chr8	124781210	124781216
chr9	29212800	29212806
chr9	36258514	36258520
chr9	127532106	127532112
chr9	130693171	130693177
chr9	130889408	130889414

chromosome	start	end
chr10	3110978	3110984
chr10	94051015	94051021
chr11	24518476	24518482
chr11	75525761	75525767
chr11	85375655	85375661
chr12	13154718	13154724
chr12	22093989	22093995
chr12	121975058	121975064
chr12	130091881	130091887
chr13	105238552	105238558
chr13	114894659	114894665
chr14	54955826	54955832
chr17	5390221	5390227
chr17	38137473	38137479
chr17	57184297	57184303
chr17	61850856	61850862
chr17	80250841	80250847
chr18	7566713	7566719
chr18	19320805	19320811
chr19	2456094	2456100
chr19	30019488	30019494
chr19	41903743	41903749
chr19	42497856	42497862
chr19	45932080	45932086
chr19	46768784	46768790
chr20	1207616	1207622
chr20	20032925	20032931
chr20	30946313	30946319
chr20	32032087	32032093
chr20	37360269	37360275
chr20	42087118	42087124
chr21	33245519	33245525
chr21	46221790	46221796
chr22	20850308	20850314
chr22	38864102	38864108
chrX	1510672	1510678
chrX	45366394	45366400
chrX	53111427	53111433
chrX	72783103	72783109

Table S1, related to Figure 1: Genomic coordinates (hg19) of the top 80 AsiSI induced DSBs identified by BLESS

	Chromatin features	Antibody used in this study	Amount of chromatin for ChIP	Status at DSBs	Proposed function in DSB repair	References	Previous genome-wide profiling
**Histone**	H3	Abcam ab1791	10µg	Decreased	Removal proposed to Increase DNA accessibility or be a consequence of resection. Unclear given the ability of nucleosome to also form on ssDNA and given that it also occur at DSB repaired by NHEJ	[1-7]	ChIP-seq in mouse cells [8] Same antibody
	H2AZ	Abcam ab4174	10µg	Transiently deposited and quickly removed	Proposed to be incorporated to promote NHEJ. Prevents unlimited resection and the use of Alt-NHEJ and SSA. Needs to be removed by INO80 and ANP32E to promote Rad51 foci formation and HR. In yeast, promotes the anchoring of persistent DSBs to nuclear periphery	[9-15]	ChIP-seq in human cells [16] Same antibody
	H1	Abcam ab17677	50µg	Decreased	Polyubiquitinated at DSB by RNF8, phosphorylated by DNA-PK and Parylated. All modifications may loosen its interaction with DNA. Removal proposed to stimulates repair by C-NHEJ and HR and retention increases Alt-NHEJ	[17-22]	ChIP-chip in human cells [23, 24] Different antibodies
	macroH2A	Millipore 07-219	50µg	Increased	Promote homologous recombination and BRCA1 recruitment/Establishment of a "heterochromatin like" (nuclease resistant, condensed) state in DSB flanking chromatin	[25-28]	ChIP-chip and ChIP-seq in human cells [29-31] Same antibody
**Modified Histones**	H2AZac	Abcam ab18262	10µg	Not assessed			ChIP-seq in human cells [31] Same antibody
	H3K79me2	Active Motif 39143	10µg	Unchanged	In vitro binds to Tudor domain of Crb2 (53BP1 orthologue) in yeast. Low affinity for 53BP1 in mammals. Promote 53BP1 foci assembly. During transcription crosstalk with H2BUb, proposed to promote relaxation	[32-37]	ChIP seq in mouse cells [8, 38] Same antibody
	H4K20me1	Active Motif 39727	10µg	Conflicting. Found as increased or unchanged	In vitro binds to Tudor domain of 53BP1 or Crb2. 53BP1 Promote 53BP1 foci assembly and NHEJ. Inhibits HR	[32, 39-45]	ChIP-seq in human cells [16] Different antibody
	H3K9me2	Abcam ab1220	10µg	Conflicting. Independently found as increased, decreased and unchanged	Promote homologous recombination/ Establishment of a "heterochromatin like" (nuclease resistant, condensed) state in DSB flanking chromatin.	[25, 46-48]	ChIP-seq in human cells [16] Same antibody
	H3K9me3	Abcam ab8898	50µg	Conflicting. Independently found as increased and unchanged	Proposed to be required for ATM activation at DSB	[46, 49].	ChIP-seq in human cells [16] Same antibody
	H3K4me2	Millipore 07-030	10µg	Conflicting. Independently found as increased and decreased	Proposed to promote relaxation	[50, 51]	ChIP-chip in human cells [52] Same antibody
	H3K36me2	Abcam ab9049	10µg	Increased	Promotes Ku70, NBS1 and MRE11 recruitment at DSB. Proposed to promote NHEJ	[46, 53, 54]	ChIP-seq in mouse cells [55, 56] Same and different antibody
	H3K36me3	Abcam ab9050	10µg	Unchanged	Interacts with LEDGF. Recruits CtIP and promotes HR	[57-59]	ChIP-seq in human cells [16] Same antibody
	H4K12ac	Abcam ab46983	10µg	transient increase	Acetylated by NuA4/Tip60. Proposed to contribute to relaxation	[60-62]	ChIP-seq in mouse cells [63] Same antibody
	H4K16ac	Millipore 07-329	200µg	Found as increased, unchanged and decreased	Required for BRCA1 recruitment and antagonizes 53BP1 binding to methylated H4K20. Proposed to facilitate resection. First decreased and then increased	[62, 64-67] 1144-51.	ChIP seq in mouse cells [68] Same antibody

	H3K56ac	Abcam ab7-307	10µg	Conflicting. Independently found as increased and decreased	Deacteylation contribute to RPA, BRCA1 and 53BP1 recruitment/ Deacetylation proposed to promote NHEJ/ Reacetylation proposed to be required for checkpoint recovery after repair	[2, 65, 69-73]	ChIP-seq in human cells [31, 74] Same antibody
	H4S1P	Novus NB21-2000	10µg	Increase at DSB (in yeast)	promotes NHEJ	[75, 76]	Not reported previously
	H4K20me2	Abcam ab9052	10µg	Described increased at DSB	Favors 53BP1 recruitment at DSB sites	[32, 39, 43, 45, 77, 78]	Not reported previously
	H2BK120Ub	Cell Signalling D11 5546P	200µg	Increased at DSB	Counteracts 53BP1 loading, stimulates range resection, promotes BRCA1, Rad51 loading and HR. Stimulates H3K4 methylation and K79me2. Proposed to promote relaxation	[50, 51, 79-81]	ChIP-seq in human cells [82] Same antibody
	H2BK120ac	Millipore 07-564	10µg	Not assessed	Antagonize H2BK120Ub. Promoted by macroH2A and PARP activity during transcription	[29]	ChIP-seq in human cells [83] Same antibody
	Ubiquitin conjugates	Millipore 04-263	200µg	Increased at DSB	Form foci upon DSB.	[84, 85]	Not reported previously
	γH2AX	Abcam ab81299	200µg	Increased at DSB	Form foci upon DSB.	[86]	ChIP-chip in human cells [87] Same antibody
**Repair proteins**	XRCC4	Abcam ab145	200µg		involved in NHEJ		
	RAD51	Santa Cruz H-92	200µg		involved in HR		
	53BP1	Novus NB100-305	200µg		Counteracts resection and BRCA1. Binds to H4K20 methylated (mainly mono and di). Binds to H2A ubiquitinated on K15. May directly interact with H2AX		
	DNA Lig IV	Genetex GTX55592	200µg		involved in NHEJ		

Table S2, Related to Figure 2, Figure 3 and STAR Methods: Histone modification summary table

Proposed functions and status at DSB (from previous reports) for each histone modifications analyzed in this study. The antibodies, the amount of chromatin used for ChIP-seq and previous reports of genome-wide mapping are also provided

References

1. Adkins, N.L., et al., *Nucleosome-like, Single-stranded DNA (ssDNA)-Histone Octamer Complexes and the Implication for DNA Double Strand Break Repair*. J Biol Chem, 2017. **292**(13): p. 5271-5281.
2. Chen, C.C., et al., *Acetylated lysine 56 on histone H3 drives chromatin assembly after repair and signals for the completion of repair*. Cell, 2008. **134**(2): p. 231-43.
3. Goldstein, M., et al., *Nucleolin mediates nucleosome disruption critical for DNA double-strand break repair*. Proc Natl Acad Sci U S A, 2013. **110**(42): p. 16874-9.
4. Li, X. and J.K. Tyler, *Nucleosome disassembly during human non-homologous end joining followed by concerted HIRA- and CAF-1-dependent reassembly*. Elife, 2016. **5**.
5. Shim, E.Y., et al., *RSC mobilizes nucleosomes to improve accessibility of repair machinery to the damaged chromatin*. Mol Cell Biol, 2007. **27**(5): p. 1602-13.

6. Tsabar, M., et al., *Re-establishment of nucleosome occupancy during double-strand break repair in budding yeast.* DNA Repair (Amst), 2016. **47**: p. 21-29.
7. Tsukuda, T., et al., *Chromatin remodelling at a DNA double-strand break site in Saccharomyces cerevisiae.* Nature, 2005. **438**(7066): p. 379-83.
8. Chronis, C., et al., *Cooperative Binding of Transcription Factors Orchestrates Reprogramming.* Cell, 2017. **168**(3): p. 442-459 e20.
9. Alatwi, H.E. and J.A. Downs, *Removal of H2A.Z by INO80 promotes homologous recombination.* EMBO Rep, 2015. **16**(8): p. 986-94.
10. Gursoy-Yuzugullu, O., M.K. Ayrapetov, and B.D. Price, *Histone chaperone Anp32e removes H2A.Z from DNA double-strand breaks and promotes nucleosome reorganization and DNA repair.* Proc Natl Acad Sci U S A, 2015. **112**(24): p. 7507-12.
11. Horigome, C., et al., *SWR1 and INO80 chromatin remodelers contribute to DNA double-strand break perinuclear anchorage site choice.* Mol Cell, 2014. **55**(4): p. 626-39.
12. Kalocsay, M., N.J. Hiller, and S. Jentsch, *Chromosome-wide Rad51 spreading and SUMO-H2A.Z-dependent chromosome fixation in response to a persistent DNA double-strand break.* Mol Cell, 2009. **33**(3): p. 335-43.
13. Taty-Taty, G.C., et al., *H2A.Z depletion impairs proliferation and viability but not DNA double-strand breaks repair in human immortalized and tumoral cell lines.* Cell Cycle, 2014. **13**(3): p. 399-407.
14. van Attikum, H., O. Fritsch, and S.M. Gasser, *Distinct roles for SWR1 and INO80 chromatin remodeling complexes at chromosomal double-strand breaks.* EMBO J, 2007. **26**(18): p. 4113-25.
15. Xu, Y., et al., *Histone H2A.Z controls a critical chromatin remodeling step required for DNA double-strand break repair.* Mol Cell, 2012. **48**(5): p. 723-33.
16. Barski, A., et al., *High-resolution profiling of histone methylations in the human genome.* Cell, 2007. **129**(4): p. 823-37.
17. Kysela, B., M. Chovanec, and P.A. Jeggo, *Phosphorylation of linker histones by DNA-dependent protein kinase is required for DNA ligase IV-dependent ligation in the presence of histone H1.* Proc Natl Acad Sci U S A, 2005. **102**(6): p. 1877-82.
18. Machida, S., et al., *Nap1 stimulates homologous recombination by RAD51 and RAD54 in higher-ordered chromatin containing histone H1.* Sci Rep, 2014. **4**: p. 4863.
19. Rosidi, B., et al., *Histone H1 functions as a stimulatory factor in backup pathways of NHEJ.* Nucleic Acids Res, 2008. **36**(5): p. 1610-23.
20. Sellou, H., et al., *The poly(ADP-ribose)-dependent chromatin remodeler Alc1 induces local chromatin relaxation upon DNA damage.* Mol Biol Cell, 2016. **27**(24): p. 3791-3799.
21. Strickfaden, H., et al., *Poly(ADP-ribosyl)ation-dependent Transient Chromatin Decondensation and Histone Displacement following Laser Microirradiation.* J Biol Chem, 2016. **291**(4): p. 1789-802.
22. Thorslund, T., et al., *Histone H1 couples initiation and amplification of ubiquitin signalling after DNA damage.* Nature, 2015. **527**(7578): p. 389-93.
23. Krishnakumar, R., et al., *Reciprocal binding of PARP-1 and histone H1 at promoters specifies transcriptional outcomes.* Science, 2008. **319**(5864): p. 819-21.
24. Millan-Arino, L., et al., *Mapping of six somatic linker histone H1 variants in human breast cancer cells uncovers specific features of H1.2.* Nucleic Acids Res, 2014. **42**(7): p. 4474-93.

25. Khurana, S., et al., *A macrohistone variant links dynamic chromatin compaction to BRCA1-dependent genome maintenance.* Cell Rep, 2014. **8**(4): p. 1049-62.
26. Leung, J.W., et al., *ZMYM3 regulates BRCA1 localization at damaged chromatin to promote DNA repair.* Genes Dev, 2017. **31**(3): p. 260-274.
27. Timinszky, G., et al., *A macrodomain-containing histone rearranges chromatin upon sensing PARP1 activation.* Nat Struct Mol Biol, 2009. **16**(9): p. 923-9.
28. Xu, C., et al., *The histone variant macroH2A1.1 is recruited to DSBs through a mechanism involving PARP1.* FEBS Lett, 2012. **586**(21): p. 3920-5.
29. Chen, H., et al., *MacroH2A1.1 and PARP-1 cooperate to regulate transcription by promoting CBP-mediated H2B acetylation.* Nat Struct Mol Biol, 2014. **21**(11): p. 981-9.
30. Gamble, M.J., et al., *The histone variant macroH2A1 marks repressed autosomal chromatin, but protects a subset of its target genes from silencing.* Genes Dev, 2010. **24**(1): p. 21-32.
31. Tropberger, P., et al., *Regulation of transcription through acetylation of H3K122 on the lateral surface of the histone octamer.* Cell, 2013. **152**(4): p. 859-72.
32. Botuyan, M.V., et al., *Structural basis for the methylation state-specific recognition of histone H4-K20 by 53BP1 and Crb2 in DNA repair.* Cell, 2006. **127**(7): p. 1361-73.
33. Giannattasio, M., et al., *The DNA damage checkpoint response requires histone H2B ubiquitination by Rad6-Bre1 and H3 methylation by Dot1.* J Biol Chem, 2005. **280**(11): p. 9879-86.
34. Huyen, Y., et al., *Methylated lysine 79 of histone H3 targets 53BP1 to DNA double-strand breaks.* Nature, 2004. **432**(7015): p. 406-11.
35. Vlaming, H., et al., *Direct screening for chromatin status on DNA barcodes in yeast delineates the regulome of H3K79 methylation by Dot1.* Elife, 2016. **5**.
36. Wakeman, T.P., et al., *Bat3 facilitates H3K79 dimethylation by DOT1L and promotes DNA damage-induced 53BP1 foci at G1/G2 cell-cycle phases.* EMBO J, 2012. **31**(9): p. 2169-81.
37. Wysocki, R., et al., *Role of Dot1-dependent histone H3 methylation in G1 and S phase DNA damage checkpoint functions of Rad9.* Mol Cell Biol, 2005. **25**(19): p. 8430-43.
38. Strikoudis, A., et al., *Regulation of transcriptional elongation in pluripotency and cell differentiation by the PHD-finger protein Phf5a.* Nat Cell Biol, 2016. **18**(11): p. 1127-1138.
39. Dulev, S., et al., *SET8 methyltransferase activity during the DNA double-strand break response is required for recruitment of 53BP1.* EMBO Rep, 2014. **15**(11): p. 1163-74.
40. Hartlerode, A.J., et al., *Impact of histone H4 lysine 20 methylation on 53BP1 responses to chromosomal double strand breaks.* PLoS One, 2012. **7**(11): p. e49211.
41. Hsiao, K.Y. and C.A. Mizzen, *Histone H4 deacetylation facilitates 53BP1 DNA damage signaling and double-strand break repair.* J Mol Cell Biol, 2013. **5**(3): p. 157-65.
42. Oda, H., et al., *Regulation of the histone H4 monomethylase PR-Set7 by CRL4(Cdt2)-mediated PCNA-dependent degradation during DNA damage.* Mol Cell, 2010. **40**(3): p. 364-76.
43. Pei, H., et al., *MMSET regulates histone H4K20 methylation and 53BP1 accumulation at DNA damage sites.* Nature, 2011. **470**(7332): p. 124-8.
44. Sanders, S.L., et al., *Methylation of histone H4 lysine 20 controls recruitment of Crb2 to sites of DNA damage.* Cell, 2004. **119**(5): p. 603-14.

45. Tuzon, C.T., et al., *Concerted activities of distinct H4K20 methyltransferases at DNA double-strand breaks regulate 53BP1 nucleation and NHEJ-directed repair.* Cell Rep, 2014. **8**(2): p. 430-8.
46. Jiang, Y., et al., *Local generation of fumarate promotes DNA repair through inhibition of histone H3 demethylation.* Nat Cell Biol, 2015. **17**(9): p. 1158-68.
47. Wu, W., et al., *Interaction of BARD1 and HP1 Is Required for BRCA1 Retention at Sites of DNA Damage.* Cancer Res, 2015. **75**(7): p. 1311-21.
48. Young, L.C., D.W. McDonald, and M.J. Hendzel, *Kdm4b histone demethylase is a DNA damage response protein and confers a survival advantage following gamma-irradiation.* J Biol Chem, 2013. **288**(29): p. 21376-88.
49. Ayrapetov, M.K., et al., *DNA double-strand breaks promote methylation of histone H3 on lysine 9 and transient formation of repressive chromatin.* Proc Natl Acad Sci U S A, 2014. **111**(25): p. 9169-74.
50. Moyal, L., et al., *Requirement of ATM-dependent monoubiquitylation of histone H2B for timely repair of DNA double-strand breaks.* Mol Cell, 2011. **41**(5): p. 529-42.
51. Nakamura, K., et al., *Regulation of homologous recombination by RNF20-dependent H2B ubiquitination.* Mol Cell, 2011. **41**(5): p. 515-28.
52. Heintzman, N.D., et al., *Distinct and predictive chromatin signatures of transcriptional promoters and enhancers in the human genome.* Nat Genet, 2007. **39**(3): p. 311-8.
53. Cao, L.L., et al., *ATM-mediated KDM2A phosphorylation is required for the DNA damage repair.* Oncogene, 2016. **35**(3): p. 301-13.
54. Fnu, S., et al., *Methylation of histone H3 lysine 36 enhances DNA repair by nonhomologous end-joining.* Proc Natl Acad Sci U S A, 2011. **108**(2): p. 540-5.
55. Kuo, A.J., et al., *NSD2 links dimethylation of histone H3 at lysine 36 to oncogenic programming.* Mol Cell, 2011. **44**(4): p. 609-20.
56. Streubel, G., et al., *The H3K36me2 Methyltransferase Nsd1 Demarcates PRC2-Mediated H3K27me2 and H3K27me3 Domains in Embryonic Stem Cells.* Mol Cell, 2018. **70**(2): p. 371-379 e5.
57. Aymard, F., et al., *Transcriptionally active chromatin recruits homologous recombination at DNA double-strand breaks.* Nat Struct Mol Biol, 2014. **21**(4): p. 366-74.
58. Carvalho, S., et al., *SETD2 is required for DNA double-strand break repair and activation of the p53-mediated checkpoint.* Elife, 2014. **3**: p. e02482.
59. Pfister, S.X., et al., *SETD2-dependent histone H3K36 trimethylation is required for homologous recombination repair and genome stability.* Cell Rep, 2014. **7**(6): p. 2006-18.
60. Bird, A.W., et al., *Acetylation of histone H4 by Esa1 is required for DNA double-strand break repair.* Nature, 2002. **419**(6905): p. 411-5.
61. Murr, R., et al., *Histone acetylation by Trrap-Tip60 modulates loading of repair proteins and repair of DNA double-strand breaks.* Nat Cell Biol, 2006. **8**(1): p. 91-9.
62. Tamburini, B.A. and J.K. Tyler, *Localized histone acetylation and deacetylation triggered by the homologous recombination pathway of double-strand DNA repair.* Mol Cell Biol, 2005. **25**(12): p. 4903-13.
63. Lopez-Atalaya, J.P., et al., *Genomic targets, and histone acetylation and gene expression profiling of neural HDAC inhibition.* Nucleic Acids Res, 2013. **41**(17): p. 8072-84.
64. Krishnan, V., et al., *Histone H4 lysine 16 hypoacetylation is associated with defective DNA repair and premature senescence in Zmpste24-deficient mice.* Proc Natl Acad Sci U S A, 2011. **108**(30): p. 12325-30.

65. Miller, K.M., et al., *Human HDAC1 and HDAC2 function in the DNA-damage response to promote DNA nonhomologous end-joining.* Nat Struct Mol Biol, 2010. **17**(9): p. 1144-51.
66. Sharma, G.G., et al., *MOF and histone H4 acetylation at lysine 16 are critical for DNA damage response and double-strand break repair.* Mol Cell Biol, 2010. **30**(14): p. 3582-95.
67. Tang, J., et al., *Acetylation limits 53BP1 association with damaged chromatin to promote homologous recombination.* Nat Struct Mol Biol, 2013. **20**(3): p. 317-25.
68. Taylor, G.C., et al., *H4K16 acetylation marks active genes and enhancers of embryonic stem cells, but does not alter chromatin compaction.* Genome Res, 2013. **23**(12): p. 2053-65.
69. Das, C., et al., *CBP/p300-mediated acetylation of histone H3 on lysine 56.* Nature, 2009. **459**(7243): p. 113-7.
70. Tjeertes, J.V., K.M. Miller, and S.P. Jackson, *Screen for DNA-damage-responsive histone modifications identifies H3K9Ac and H3K56Ac in human cells.* EMBO J, 2009. **28**(13): p. 1878-89.
71. Toiber, D., et al., *SIRT6 recruits SNF2H to DNA break sites, preventing genomic instability through chromatin remodeling.* Mol Cell, 2013. **51**(4): p. 454-68.
72. Vempati, R.K., et al., *p300-mediated acetylation of histone H3 lysine 56 functions in DNA damage response in mammals.* J Biol Chem, 2010. **285**(37): p. 28553-64.
73. Yuan, J., et al., *Histone H3-K56 acetylation is important for genomic stability in mammals.* Cell Cycle, 2009. **8**(11): p. 1747-53.
74. Lo, K.A., et al., *Genome-wide profiling of H3K56 acetylation and transcription factor binding sites in human adipocytes.* PLoS One, 2011. **6**(6): p. e19778.
75. Cheung, W.L., et al., *Phosphorylation of histone H4 serine 1 during DNA damage requires casein kinase II in S. cerevisiae.* Curr Biol, 2005. **15**(7): p. 656-60.
76. Utley, R.T., et al., *Regulation of NuA4 histone acetyltransferase activity in transcription and DNA repair by phosphorylation of histone H4.* Mol Cell Biol, 2005. **25**(18): p. 8179-90.
77. Fradet-Turcotte, A., et al., *53BP1 is a reader of the DNA-damage-induced H2A Lys 15 ubiquitin mark.* Nature, 2013. **499**(7456): p. 50-4.
78. Pellegrino, S., et al., *Replication-Coupled Dilution of H4K20me2 Guides 53BP1 to Pre-replicative Chromatin.* Cell Rep, 2017. **19**(9): p. 1819-1831.
79. Sun, Z.W. and C.D. Allis, *Ubiquitination of histone H2B regulates H3 methylation and gene silencing in yeast.* Nature, 2002. **418**(6893): p. 104-8.
80. Zeng, M., et al., *CRL4(Wdr70) regulates H2B monoubiquitination and facilitates Exo1-dependent resection.* Nat Commun, 2016. **7**: p. 11364.
81. Zhou, L., et al., *Evidence that ubiquitylated H2B corrals hDot1L on the nucleosomal surface to induce H3K79 methylation.* Nat Commun, 2016. **7**: p. 10589.
82. Park, S.H., et al., *Type I interferons and the cytokine TNF cooperatively reprogram the macrophage epigenome to promote inflammatory activation.* Nat Immunol, 2017. **18**(10): p. 1104-1116.
83. Wang, Z., et al., *Combinatorial patterns of histone acetylations and methylations in the human genome.* Nat Genet, 2008. **40**(7): p. 897-903.
84. Butler, L.R., et al., *The proteasomal de-ubiquitinating enzyme POH1 promotes the double-strand DNA break response.* EMBO J, 2012. **31**(19): p. 3918-34.

85. Gudjonsson, T., et al., *TRIP12 and UBR5 suppress spreading of chromatin ubiquitylation at damaged chromosomes.* Cell, 2012. **150**(4): p. 697-709.
86. Rogakou, E.P., et al., *DNA double-stranded breaks induce histone H2AX phosphorylation on serine 139.* J Biol Chem, 1998. **273**(10): p. 5858-68.
87. Iacovoni, J.S., et al., *High-resolution profiling of gammaH2AX around DNA double strand breaks in the mammalian genome.* EMBO J, 2010. **29**(8): p. 1446-57.
