# Supplementary material for: Comprehensive Mapping of Histone Modifications at DNA Double-Strand Breaks Deciphers Repair Pathway Chromatin Signatures
Source: Mol Cell. 2018 Oct 18;72(2):250–262.e6. doi: 10.1016/j.molcel.2018.08.020 (PMC6202423; doi:10.1016/j.molcel.2018.08.020)
Supplement: Document S2. Article plus Supplemental Information [file mmc2.pdf]

Molecular Cell

Article

Comprehensive Mapping of Histone Modifications at DNA Double-Strand Breaks Deciphers Repair Pathway Chromatin Signatures

Graphical Abstract

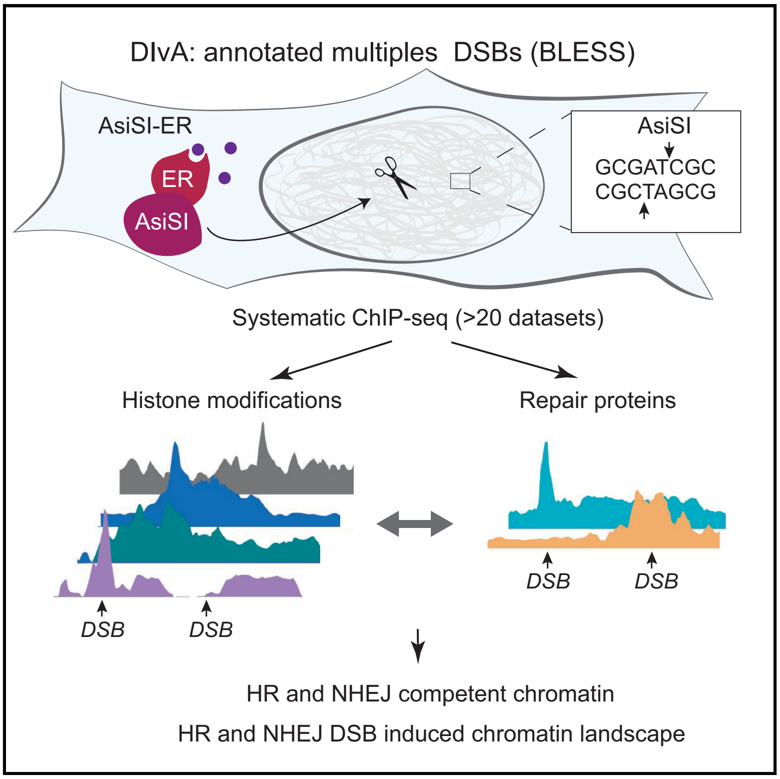

Highlights

- DSB-chromatin landscape and HR/NHEJ chromatin signatures uncovered by ChIP-seq
- H2BK120 undergoes a switch from ubiquitination to acetylation at a local scale
- H1 is removed and ubiquitin accumulates on entire γH2AX domains, mainly at HR DSB
- 53BP1 spreads over megabase-sized domains, mostly in G1 at HR-prone DSBs

Authors

Thomas Clouaire, Vincent Rocher, Anahita Lashgari, ..., Krzysztof Ginalski, Jacques Côté, Gaëlle Legube

Correspondence

thomas.clouaire@univ-tlse3.fr (T.C.), gaelle.legube@univ-tlse3.fr (G.L.)

In Brief

Using ChIP-seq in a cell line where multiple annotated DNA double-strand breaks can be induced on the human genome, Clouaire et al. report a comprehensive view of the chromatin landscape set up at DSBs and decipher the chromatin signature associated with HR and NHEJ repair.

Clouaire et al., 2018, Molecular Cell 72, 250–262
October 18, 2018 © 2018 The Authors. Published by Elsevier Inc.
https://doi.org/10.1016/j.molcel.2018.08.020

Molecular Cell
Article

Comprehensive Mapping of Histone Modifications at DNA Double-Strand Breaks Deciphers Repair Pathway Chromatin Signatures

Thomas Clouaire,[1,*] Vincent Rocher,[1] Anahita Lashgari,[2] Coline Arnould,[1] Marion Aguirrebengoa,[1] Anna Biernacka,[3] Magdalena Skrzypczak,[3] François Aymard,[1] Bernard Fongang,[4] Norbert Dojer,[4,5] Jason S. Iacovoni,[6] Maga Rowicka,[4] Krzysztof Ginalski,[3] Jacques Côté,[2] and Gaëlle Legube[1,7,8,*]
[1]LBCMCP, Centre de Biologie Integrative (CBI), CNRS, Université de Toulouse, UT3, Toulouse 31062, France
[2]St-Patrick Research Group in Basic Oncology, Laval University Cancer Research Center, Oncology Axis-CHU de Québec-Université Laval Research Center, Quebec City, QC G1R 3S3, Canada
[3]Laboratory of Bioinformatics and Systems Biology, Centre of New Technologies, University of Warsaw, Zwirki i Wigury Warsaw 93, 02-089, Poland
[4]Department of Biochemistry and Molecular Biology, University of Texas Medical Branch, Galveston, TX 77555-0615, USA
[5]Institute of Informatics, University of Warsaw, Banacha 2, 02-097 Warsaw, Poland
[6]Bioinformatic Plateau I2MC, INSERM and University of Toulouse, Toulouse 31062, France
[7]Senior author
[8]Lead Contact
*Correspondence: thomas.clouaire@univ-tlse3.fr (T.C.), gaelle.legube@univ-tlse3.fr (G.L.)
https://doi.org/10.1016/j.molcel.2018.08.020

SUMMARY

Double-strand breaks (DSBs) are extremely detrimental DNA lesions that can lead to cancer-driving mutations and translocations. Non-homologous end joining (NHEJ) and homologous recombination (HR) represent the two main repair pathways operating in the context of chromatin to ensure genome stability. Despite extensive efforts, our knowledge of DSB-induced chromatin still remains fragmented. Here, we describe the distribution of 20 chromatin features at multiple DSBs spread throughout the human genome using ChIP-seq. We provide the most comprehensive picture of the chromatin landscape set up at DSBs and identify NHEJ- and HR-specific chromatin events. This study revealed the existence of a DSB-induced monoubiquitination-to-acetylation switch on histone H2B lysine 120, likely mediated by the SAGA complex, as well as higher-order signaling at HR-repaired DSBs whereby histone H1 is evicted while ubiquitin and 53BP1 accumulate over the entire γH2AX domains.

INTRODUCTION

DNA double-strand breaks (DSBs) are extremely detrimental since they can lead to mutations and chromosomal rearrangements. DSBs arise from various environmental stresses and upon developmentally scheduled activation of endonucleases, but they can also arise physiologically during replication and transcription. Anomalies in the DSB repair apparatus are responsible for premature aging and neurodegenerative syndromes and are strongly implicated in cancer onset and progression.

DSBs are mainly repaired by two partially redundant, yet profoundly different, pathways: homologous recombination (HR) and non-homologous end joining (NHEJ) (for review, see Mladenov et al., 2016). HR uses an intact copy of the damaged locus as a template and involves many factors for DSB detection, 5′ end resection, homology search, strand invasion, and resolution. In contrast, NHEJ repair machineries require no or limited resection and can join the two broken ends with no or minimal homology. Inaccuracy, failure, or misuse of each of these pathways can trigger very different consequences on the genome. DSB repair pathway choice can be influenced by cell cycle phase (Hustedt and Durocher, 2016), DNA end complexity (Schipler and Iliakis, 2013), and the type of damaged locus (Clouaire and Legube, 2015; Engel et al., 2018).

In eukaryotes, DSB repair occurs in the context of chromatin. Chromatin is a highly dynamic structure, affected by histone post-translational modifications, DNA methylation, or incorporation of histone variants (for review, see Soshnev et al., 2016; Talbert and Henikoff, 2017). Chromatin modifications can alter the stability of the histone octamer onto DNA but also be specifically recognized by "reader" modules found in chromosomal proteins as well as subunits of DNA transaction machineries. All together, nucleosome modifications regulate DNA accessibility; the stiffness, flexibility, and mobility of chromatin within the nucleus; and the recruitment of molecular machines ensuring transcription, replication, and repair.

Key aspects in the interplay between DSB repair and chromatin environment have already emerged (Table S2). H2AX is rapidly phosphorylated by ATM (and named γH2AX) over several megabases surrounding the break (Caron et al., 2015; Iacovoni et al., 2010; Rogakou et al., 1998; Savic et al., 2009). In parallel, histone acetyltransferases and deacetylases tightly control acetylation levels of several residues of H3, H4, and H2A (Dobbin et al.,

 © 2018 The Authors. Published by Elsevier Inc.
This is an open access article under the CC BY license (http://creativecommons.org/licenses/by/4.0/).

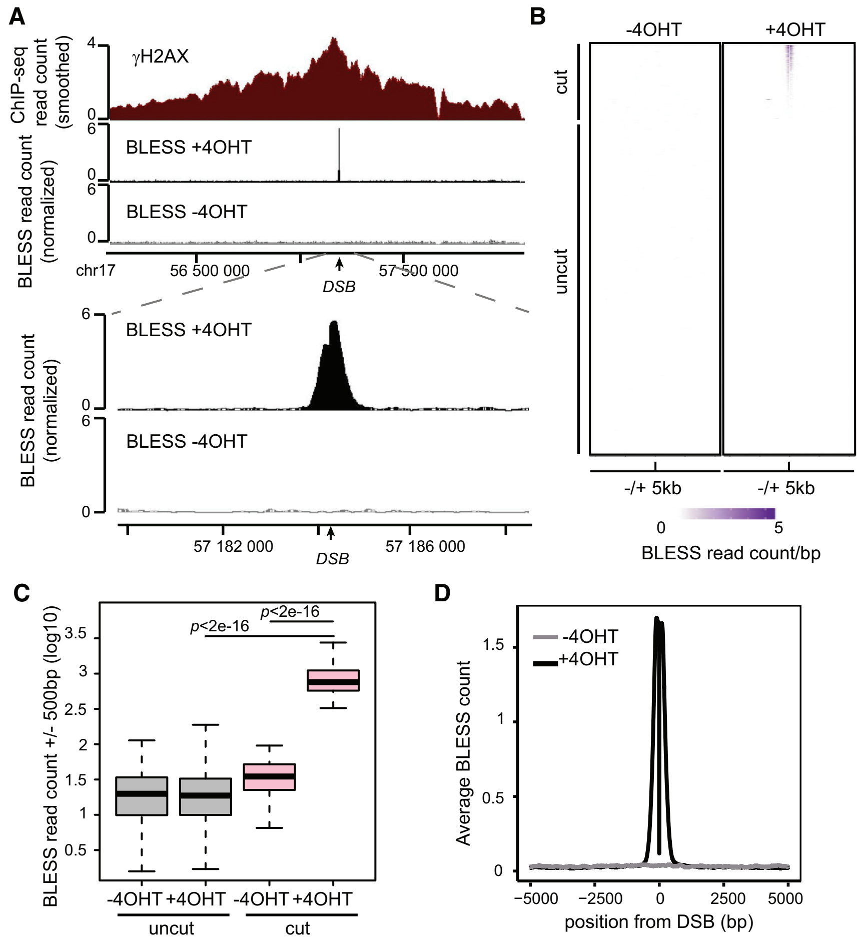

Figure 1. BLESS Mapping of AsiSI-Induced DSB

(A) Genome browser screenshot representing γH2AX ChIP-seq and BLESS signal at a single DSB located on human chromosome 17.

(B) Heatmap representation of the BLESS profile for the 1,211 predicted AsiSI sites in the human genome in DIvA cells untreated (−4OHT) or treated for 4 hr (+4OHT). DSBs are ordered based on decreasing read count in a 1 kb window.

(C) Boxplot representing BLESS signals (1 kb window) for the 80 cut (pink) and the 1,131 uncut (gray) AsiSI sites in untreated (−4OHT) or treated (+4OHT) DIvA cells. p values were calculated using two-sample Wilcoxon tests.

(D) Average BLESS count profile for the 80 induced DSBs before (−4OHT, gray line) and after induction (+4OHT, black line).

2013; Gong et al., 2015; Jacquet et al., 2016; Lee et al., 2010; Miller et al., 2010; Ogiwara et al., 2011; Toiber et al., 2013) to regulate chromatin relaxation near DSBs. Acetylated histones also participate in the recruitment of nucleosome remodeling factors, enhancing DSB accessibility and facilitating resection (Bennett and Peterson, 2015; Lee et al., 2010; Toiber et al., 2013). Similarly, histone methyltransferases and demethylases can regulate recruitment and/or stabilization of repair proteins (CtIP, 53BP1, BRCA1 …) (Table S2). Moreover, ubiquitination and sumoylation pathways contribute to DSB-induced chromatin reorganization (for review, see Schwertman et al., 2016). All together, these specific chromatin modifications generate a chromatin state permissive for repair but also directly contribute to the recruitment of DSB repair machineries, repair pathway choice, and the activation of the DNA damage checkpoint.

Yet the definite map of DSB-induced chromatin modifications and their respective involvement in DSB repair remain largely unknown. Furthermore, NHEJ and HR repair pathways conceivably require very different chromatin settings. Since chromatin structure plays a central role in DNA accessibility and flexibility, an in-depth characterization of the chromatin that assembles at DSBs represents a critical step in understanding how DSB repair machineries operate in the whole nucleus to restore the original DNA sequence and avoid deleterious genome rearrangements.

Here, using high-throughput genomic approaches in a standardized system in which multiple DSBs are induced at defined positions across the human genome, we report the first comprehensive picture of the chromatin landscape induced around DSBs and its relationship with individual repair pathways.

RESULTS

High-Resolution Mapping of AsiSI-Induced DSBs

To characterize the chromatin landscape at DSBs, we used the DIvA (DSB inducible via AsiSI) cell line, which enables the controlled induction (using 4-hydroxytamoxifen, or 4OHT) of multiple well-annotated DSBs scattered throughout the human genome (Aymard et al., 2014; Iacovoni et al., 2010). We previously showed that *in vivo*, AsiSI does not produce a DSB at every target site on the genome and that γH2AX ChIP-seq can serve as a good proxy to identify sites efficiently cut by AsiSI (Aymard et al., 2014; Iacovoni et al., 2010). However, this is an indirect assessment of the genomic lesion, and γH2AX accumulation is strongly affected by genomic and epigenomic features (Caron et al., 2012; Iacovoni et al., 2010). To unambiguously identify the position of AsiSI-induced DSBs and obtain a quantitative measurement of the DSB generation rate, we used the recently developed BLESS method (Crosetto et al., 2013; Mitra et al., 2015). We detected sharp BLESS peaks located precisely at predicted AsiSI sites following a 4 hr treatment of DIvA cells with 4OHT and minimal signal in untreated conditions (Figures 1A and S1A). Further analysis revealed that out of the 1,211 predicted AsiSI sites in the human genome, 174 showed a signal significantly higher than background (Figures S1B and 1B). This is in relatively good agreement with our previous estimates of the number of induced DSBs inferred from γH2AX accumulation and with BLISS data in DIvA cells (Aymard et al., 2014; Iannelli et al., 2017). However, we could detect clear

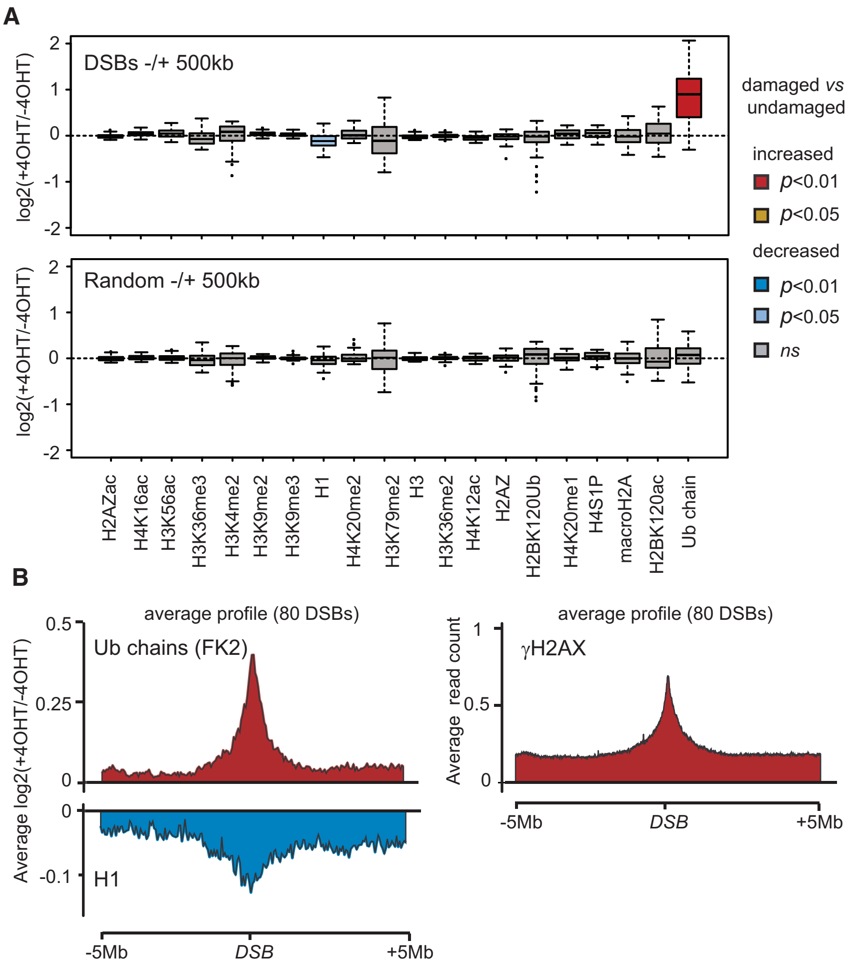

Figure 2. Large-Scale DSB-Induced Chromatin Changes

(A) Boxplot representing the ChIP-seq enrichment ratio between 4OHT-treated and untreated DIvA cells (expressed as a log_2 ratio) for 80 DSBs (upper panel) or 80 randomly picked genomic regions (lower panel) over a 1 Mb window. Boxes are colored according to p values (two-sample Wilcoxon tests) and the nature of the change.

(B) Left: Average profile of the enrichment between 4OHT-treated and untreated DIvA cells for ubiquitin and H1 over 80 DSBs in a 10 Mb window. Values are expressed as log_2 ratios. Right: Average profile of γH2AX in 4OHT-treated DIvA cells.

differences between γH2AX and BLESS signals, confirming that γH2AX does not strictly reflect DSB induction rate (Figure S1A, compare left and right panel; Figure S1C). To remove any site that may only be partially cleaved, we focused on a robust set of 80 DSBs defined by BLESS analysis that are significantly induced by 4OHT treatment (Figures 1B–1D, Table S1). These DSBs are characterized by low levels in heterochromatin-associated features such as DNA methylation and H3K9me3 (Figure S1D), and about half are located near active promoters (Figure S1E). Yet analyzing ChIP-seq enrichment for the elongating form of RNA polymerase II (S2P) revealed that not all sites necessarily reside within actively transcribed regions (Figure S1D). We retained this validated set of DSBs, representing both active and inactive euchromatic regions, for further analysis.

Mapping of Histone Modifications at AsiSI-Induced DSBs

To obtain a comprehensive picture of the chromatin landscape at DSBs, we generated 20 ChIP-seq profiles, in both damaged (4OHT-treated) and undamaged cells. These included linker and core histones, histone variants, and post-translational modifications that were proposed to play a role in DSB repair (Table S2). We also generated a genome-wide map for chromatin-associated ubiquitin using the FK2 antibody that recognizes a wide variety of ubiquitin conjugates. ChIP-qPCR revealed that each antibody efficiently immunoprecipitated chromatin (Figure S2A). More importantly, we could recapitulate previously reported profiles for each feature over transcription units, categorized by transcriptional activity (Figure S2B). Our H4S1P mapping, which was lacking in mammalian cells, is in agreement with its proposed function in transcriptional regulation and accumulation over gene bodies in yeast (Utley et al., 2005).

Given that γH2AX accumulation can spread up to two megabases (Mb) from the actual breakpoint (Iacovoni et al., 2010), we first examined DSB-induced chromatin modifications over a 1 Mb window. At this scale, ubiquitination is largely induced ($p < 0.01$, two-sample Wilcoxon test) and histone H1 occupancy significantly reduced ($p < 0.05$, two-sample Wilcoxon test) compared to randomly selected genomic regions (Figure 2A). We could not detect significant DSB-induced changes for any other histone modifications within these megabase-sized genomic windows surrounding breaks. Average profiles revealed that ubiquitin conjugates accumulated over approximately 2 Mb, similarly to γH2AX spreading (Figures 2B and S2C). DSB-induced H1 depletion is maximal over a similar region but remains detectable over a slightly more extended area (Figure 2B, bottom panel, and Figure S2C). All together, this shows that DSBs induce few but large-scale (megabase-sized) chromatin reshufflings, such as phosphorylation of H2AX, accumulation of ubiquitin, and depletion of the linker histone H1.

We next considered that DSB could cause chromatin modifications much closer to the break and examined 1 kb windows surrounding AsiSI-induced DSBs. Interestingly, we found that among the 20 chromatin modifications analyzed, 6 were significantly decreased (H3K79me2, H3, H3K36me2, H4K12ac, H2AZ, and H2BK120ub) and 5 significantly increased (H4S1P,

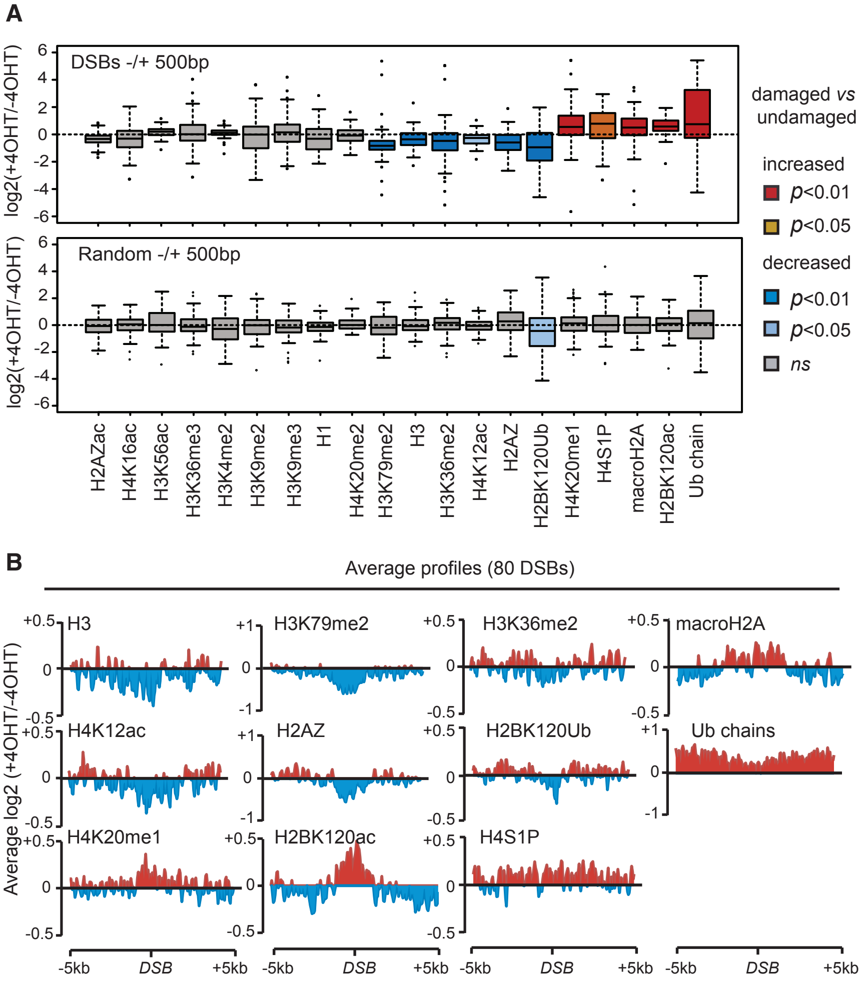

Figure 3. Narrow-Scale DSB-Induced Chromatin Changes

(A) Boxplot representing the ChIP-seq enrichment ratio between treated and untreated DIvA cells (expressed as a $\log_2$ ratio) for 80 DSBs (upper panel) or 80 randomly picked genomic regions (lower panel) over a 1 kb window. Boxes are colored according to p values (two-sample Wilcoxon tests) and the nature of the change.

(B) Average profile of the enrichment between treated and untreated DIvA cells for 12 modifications, showing significant differences between each condition over 80 DSBs in a 10 kb window. Values are expressed as $\log_2$ ratios.

H4K20me1, macroH2A, H2BK120ac, and ubiquitin) near DSBs (Figure 3A, top panel) when compared to random sites (Figure 3A, bottom panel). Average profiles showed that these proximal DSB-induced chromatin alterations spread over different scales, ranging from 1 to 10 kb (Figure 3B). Decreases in H3, H2AZ, H4K12ac, H2BK120ub, and H3K79me2 and the increases in H4K20me1 and H2BK120ac took place over 2–4 kb windows. MacroH2A and H4S1P showed a DSB induction spanning 5–6 kb and approximately 10 kb, respectively. In most cases, normalizing to histone H3 did not alter the observed changes, with the exception of H4K12ac and H3K36me2, which may thus only reflect changes in nucleosome occupancy (Figure S2D). All together, our systematic ChIP-seq mapping indicates that DSBs trigger many alterations to chromatin structure occurring over many different scales, ranging from 1 kb up to several megabases.

More specifically, our data suggest that in response to DSBs, H2BK120 undergoes a switch from ubiquitination to acetylation (Figure 3A). Time-course experiments confirmed the progressive loss of H2BK120ub upon DSB induction (Figure S3A). Interestingly, SAGA, a prominent chromatin-modifying complex, acetylates histones H3 and H2B and also displays deubiquitinase activity toward H2BK120 (Helmlinger and Tora, 2017). SAGA has also been previously implicated in class switch recombination and response to ionizing radiation (Ramachandran et al., 2016). We show that *in vitro*, the native human SAGA complex (affinity-purified through its specific SUPT7L subunit; Figure S3B) displays both H2BK120 deubiquitinase and acetyltransferase activities, making it a primary candidate for this switch (Figure S3C). Furthermore, depletion of SUPT7L as well as PCAF, one HAT paralog present in the SAGA complex (Figures S3D and S3E), triggered decreases in both HR at an endogenous locus (*LMNA*) following CRISPR/Cas9 breakage (Pauty et al., 2017) and NHEJ in a cell reporter system (Jacquet et al., 2016) (Figures S3F and S3G), indicating that SAGA indeed contributes to DSB repair in human cells.

Damage in Active Chromatin Undergoes Preferential Repair via HR

It is now well established that the local chromatin structure can influence how a given DSB is handled and subsequently repaired (for review, see Clouaire and Legube, 2015). We thus set out to understand the contribution of chromatin to repair pathway choice by defining chromatin states favorable to HR and NHEJ. We defined subsets of BLESS-validated DSBs that are preferentially repaired by HR or NHEJ (30 in each category) by sorting them according to the binding ratio for RAD51 and XRCC4 using ChIP-seq data obtained in 4OHT-treated DIvA cells (Aymard et al., 2014). Average profiles confirmed the prominent accumulation and spreading of RAD51 over 5 kb at DSBs preferentially repaired by HR (Figure 4A). On the other hand, XRCC4 recruitment appeared similar for both sets of DSBs, with a multimodal distribution over 500 bp (Figures 4A and S4A), likely related to XRCC4/XLF filaments at DSBs (Ropars et al., 2011). Importantly, BLESS intensity was comparable between HR and NHEJ DSBs (Figures 4A and 4B), suggesting

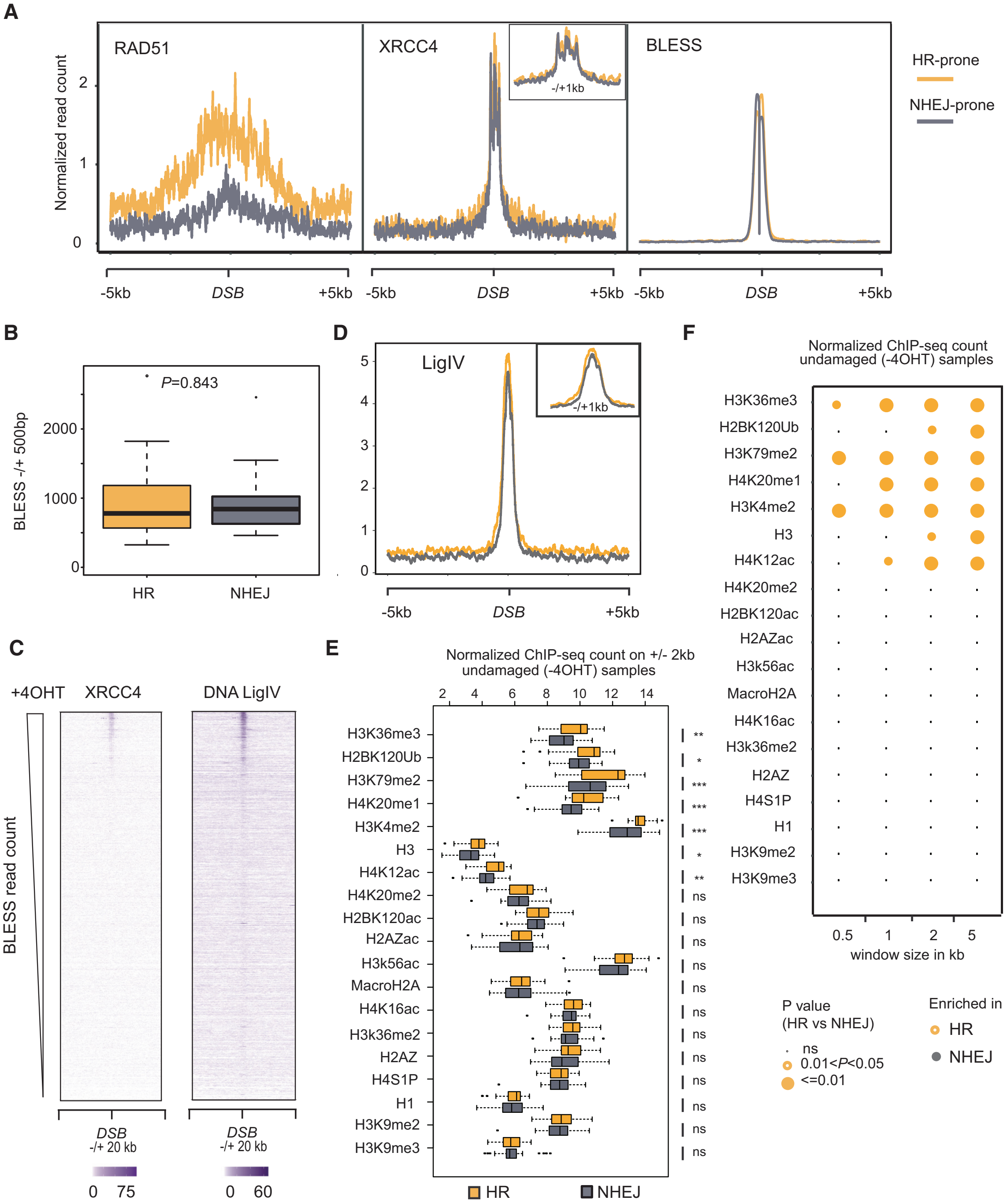

Figure 4. Chromatin Features Associated with HR-Prone DSBs

(A) Average profile for RAD51 ChIP-seq, XRCC4 ChIP-seq, and BLESS for 30 HR (yellow) or 30 NHEJ (gray) DSBs. HR and NHEJ DSBs were defined based upon the RAD51/XRCC4 binding ratio (see STAR Methods).

(B) Boxplot representing BLESS read count (1 kb window) around 30 HR or 30 NHEJ DSBs. p value was calculated using two-sample Wilcoxon test.

(C) Heatmap representing the XRCC4 and DNA Ligase IV ChIP-seq signals on a 40 kb window centered around all AsiSI sites, ordered based on the BLESS level.

(D) Same as (A) for DNA Ligase IV ChIP-seq.

(E) Boxplot representing the ChIP-seq read count (4 kb window) for each histone modification in untreated cells for 30 HR (yellow) and 30 NHEJ (gray) DSBs. p values were calculated using two-sample Wilcoxon test. *^{r}p < 0.05, **^{r}p < 0.01; p > 0.05 is not significant (ns).

(F) Circle plot representing p values (from two-sample Wilcoxon test) when comparing ChIP-seq signal for HR and NHEJ DSB using increasing window size.

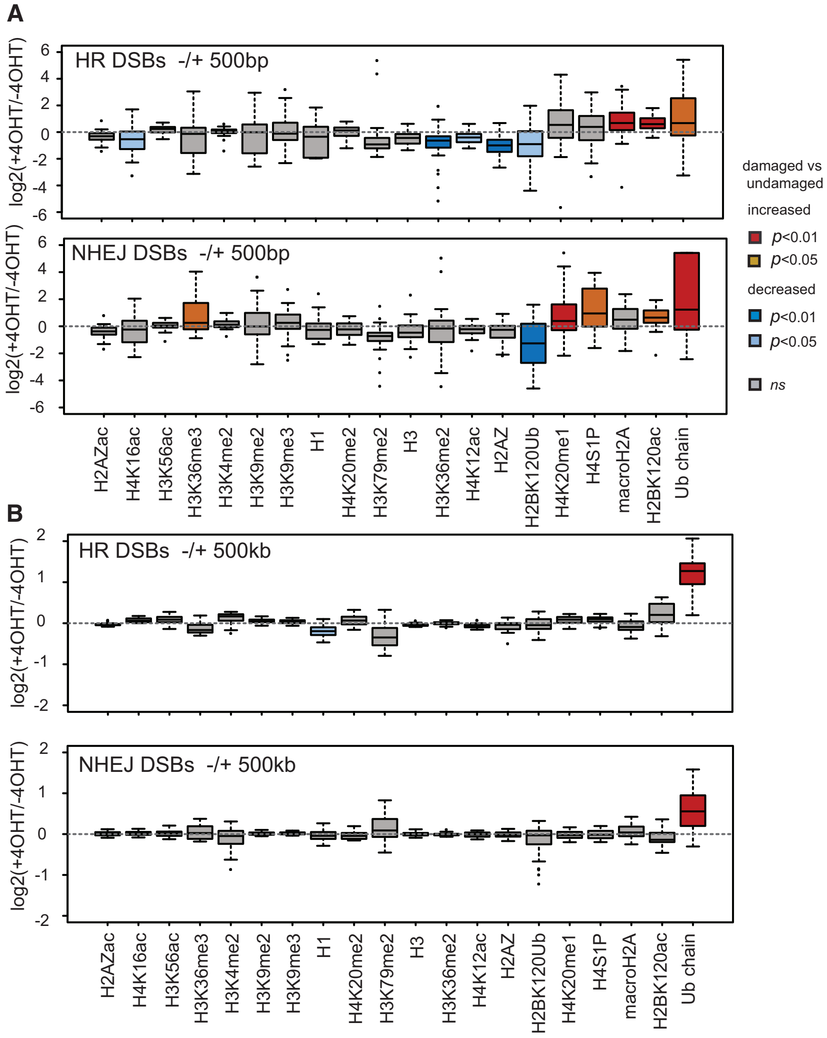

Figure 5. HR and NHEJ-Induced Chromatin Changes

(A) Boxplot representing the ChIP-seq enrichment ratio between treated and untreated DIvA cells (expressed as a log_2 ratio) for 30 HR (upper panel) or 30 NHEJ (lower panel) over a 1 kb window. Boxes are colored according to p values (two-sample Wilcoxon tests) and the nature of the change.

(B) Same as (A) for a 1 Mb window.

that differences in RAD51 binding do not solely depend on the DSB induction rate. Since NHEJ is a relatively fast process compared to HR, it remained possible that some sites could be cleaved, repaired, and mutated with a very fast kinetics, therefore not appearing in our set of NHEJ-prone DSBs. To address this, we performed XRCC4 ChIP-seq at various times of 4OHT treatment (1 hr, 4 hr, and 24 hr) and found that XRCC4 binding was indeed very similar at DSB sites at each time point (Figures S4C–S4E). More importantly, we could not detect any site showing XRCC4 enrichment at 1 hr or 24 hr and not at 4 hr, which strongly validated our HR and NHEJ categories (Figures S4C–S4E). To confirm that XRCC4 binding was indeed indicative of NHEJ, we also performed ChIP-seq using an antibody directed against DNA ligase IV, another key component in this pathway. As expected, DNA ligase IV showed a very similar enrichment when compared to XRCC4 at all AsiSI sites (Figures 4C, 4D, and S5A–S5C). All together, these analyses allowed us to define a robust set of DSBs either prone to engage HR (HR-prone) or not (NHEJ-prone).

Next, we compared basal enrichment (before DSB induction) for each modification at the vicinity of DSBs (2 kb) preferentially repaired by HR or NHEJ. In agreement with our previous finding (Aymard et al., 2014), H3K36me3 was significantly more abundant proximal to DSBs repaired by HR (Figure 4E). HR-competent chromatin also contained elevated levels of H3K79me2, H4K20me1, H2BK120ub, H3K4me2, H4K12ac, and core histone H3 (Figure 4E). This result was confirmed when comparing chromatin signatures over increasing window sizes (Figure 4F). Consistently, we found a similar trend when using DNA ligase IV instead of XRCC4 to determine HR and NHEJ categories (Figure S5D). On the other hand, we found no evidence of a specific signature that may actively favor repair by NHEJ (Figures 4E, 4F, and S5D). All together, these data identified a specific chromatin structure that is competent for HR repair. The HR-specific signature primarily consists of features linked to active transcription (Figure S2B). In agreement, HR-prone DSBs were found to be significantly enriched in the nuclear compartment A1, which was defined by its specific long-range interaction pattern by Hi-C analysis and shown to be enriched in transcriptionally active regions (Rao et al., 2014) (Figure S4B). Hence, our data strongly favor the hypothesis that DSBs induced in active genes are biased toward HR.

Chromatin Modifications upon HR and NHEJ Repair: Deciphering the Repair Pathway Chromatin Signature

DSB repair by HR and NHEJ depends on two separate mechanisms and operates in distinct chromatin contexts (see above). Hence, it is possible that each pathway involves different remodeling events compatible with its particular repair mechanism. We therefore considered the possibility that DSB-induced alterations in chromatin could be specific for HR- and NHEJ-prone DSBs. Notably, focusing on events occurring proximal to the break point (1 kb windows), we found that while some chromatin marks significantly changed after break induction only at DSBs repaired by HR or by NHEJ, others occurred irrespective of the repair mechanism (Figure 5A). We also examined DSB-induced chromatin changes at HR- and NHEJ-prone sites at various distances from the DSBs (Figure S5E). MacroH2A deposition and H4S1 phosphorylation occurred upon repair by both HR and NHEJ (Figures 5A and S5E). Similarly, the switch from

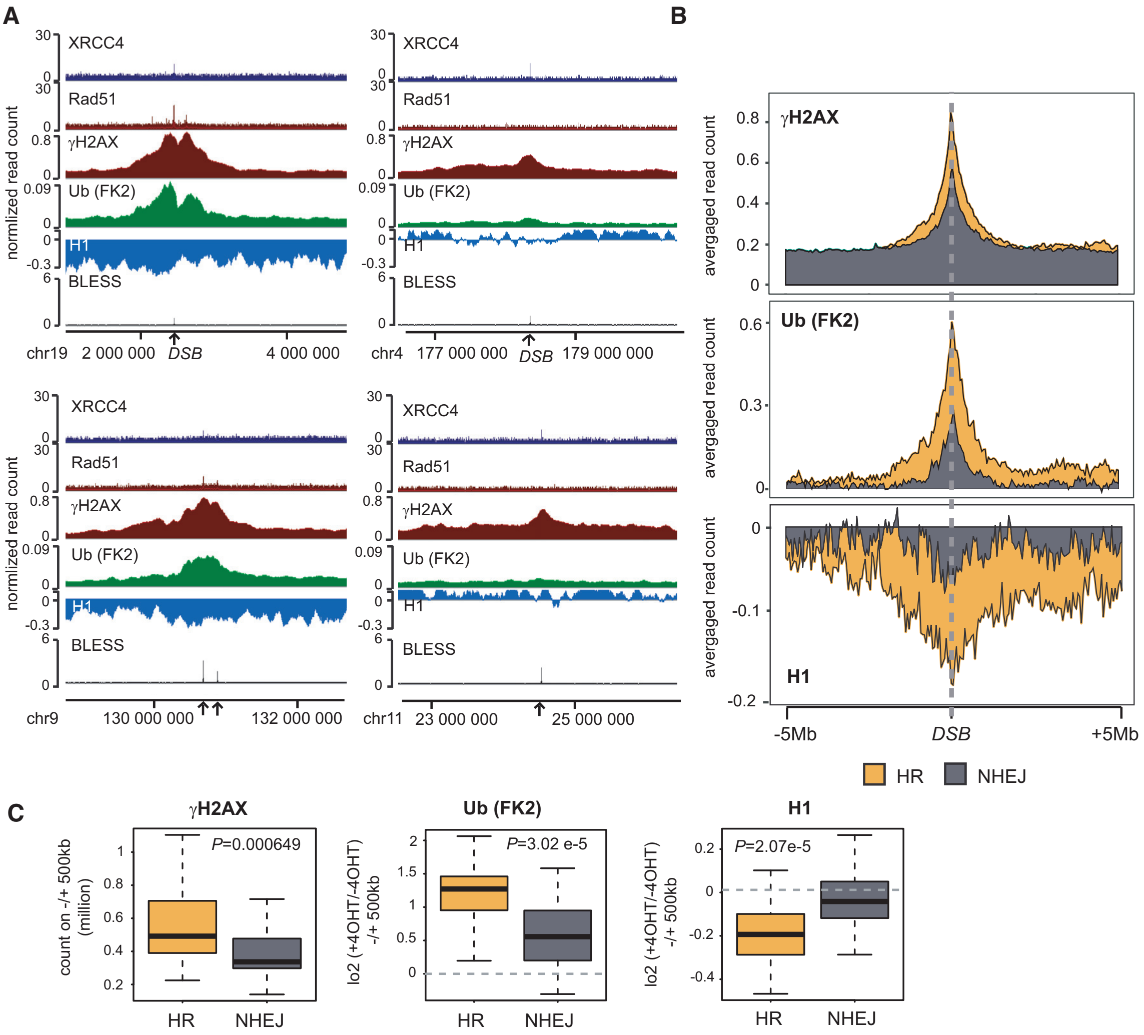

Figure 6. Acute Signaling at HR-Prone DSBs

(A) Genome Browser screenshots representing ChIP-seq signals for XRCC4, RAD51, γH2AX, ubiquitin, H1, and BLESS for two HR-DSBs (left) and two NHEJ DSBs (right). Data are expressed as read count (from 4OHT-treated samples) for XRCC4, RAD51, and γH2AX ChIP-seq and BLESS. Data for ubiquitin (FK2) and H1 are expressed as a $\log_2$ ratio between treated and untreated cells.

(B) Average profile for γH2AX ChIP-seq (read count in OHT treated samples), ubiquitin, and H1 (as a $\log_2$ ratio between treated and untreated cells) for 30 HR (yellow) and 30 NHEJ (gray) DSBs in a 10 Mb window.

(C) Boxplot representing ChIP-seq read count from treated cells (γH2AX) or $\log_2$ ratio between treated and untreated cells (ubiquitin [FK2] and H1) for 30 HR (yellow) or 30 NHEJ (gray) DSBs in a 1 Mb window. p values were calculated using two-sample Wilcoxon tests.

H2BK120 ubiquitination to acetylation also occurred at all DSBs (Figures 5A and S5E), in agreement with our above finding that depletion of the SAGA complex affects both HR and NHEJ (Figures S3F and S3G). In contrast, reduction in H2AZ and H3 occupancy and decrease in H3K36/79 dimethylation and in H4K12/K16 acetylation following DSB induction were significant only at sites prone to HR (Figures 5A and S5E). Conversely, DSB induction at sites repaired by NHEJ was accompanied by a significant increase in H3K36me3 and H4K20me1, modifications not found at HR-repaired DSBs (Figures 5A and S5E). Hence, we were able to define a local histone modification signature, which occurs proximal to DSBs, associated with the two main repair pathways.

We also interrogated our data for pathway-specific events occurring within 1 Mb from the break. Linker histone H1 depletion and ubiquitin accumulation were significantly more pronounced within 1 Mb of HR-repaired sites compared to NHEJ-prone DSBs (Figure 5B). Examination of individual (Figure 6A) or averaged (Figure 6B) profiles confirmed that DSB-induced changes in H1 and ubiquitin were accentuated at HR sites, with H1 depletion being barely detectable at NHEJ sites. We also detected increased megabase-wide γH2AX levels for HR sites compared to NHEJ sites (Figures 6A and 6B). Accumulation of ubiquitin conjugate, H2AX phosphorylation, and H1 depletion were indeed significantly reinforced surrounding DSBs repaired by HR compared to those repaired by NHEJ (Figure 6C). This finding suggests that damage

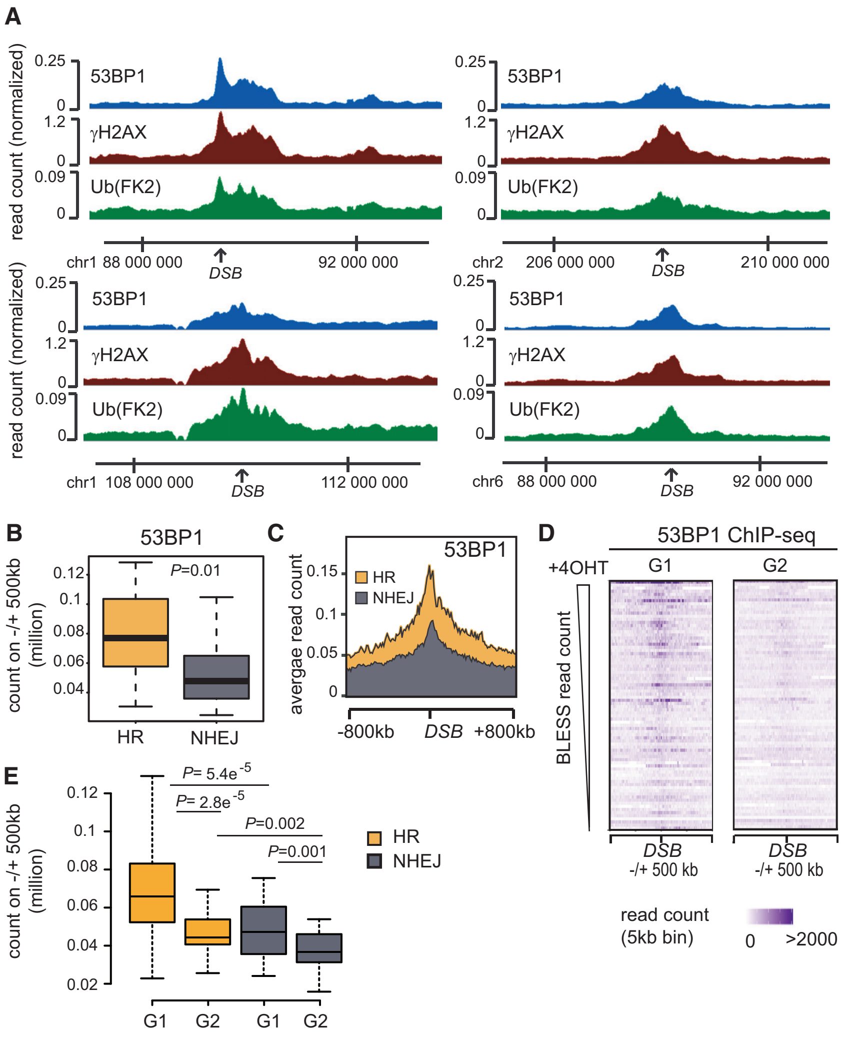

Figure 7. 53BP1 Preferentially Accumulates at HR-Prone DSBs Specifically in G1

(A) Genome Browser screenshots representing ChIP-seq signals (from 4OHT-treated samples) for 53BP1, γH2AX, and ubiquitin.

(B) Boxplot representing 53BP1 enrichment in a 1 Mb window for 30 HR (yellow) and 30 NHEJ (gray) DSBs. p value was calculated using two-sample Wilcoxon test.

(C) Average profile for 53BP1 ChIP-seq (read count in 4OHT-treated cells) for 30 HR (yellow) and 30 NHEJ (gray) DSBs in a 1.6 Mb window (upper panel).

(D) Heatmap representing 53BP1 ChIP-seq count obtained in G1- and G2-synchronized cells as indicated, on a 1 Mb window surrounding 80 DSBs induced by AsiSI. DSBs are sorted by BLESS read counts.

(E) Boxplot representing 53BP1 enrichment in a 1 Mb window for 30 HR (yellow) and 30 NHEJ (gray) DSBs in G1- and G2-synchronized cells as indicated. p values were calculated using two-sample Wilcoxon test.

occurring in active chromatin and repaired by HR can trigger acute and broad-scale remodeling within high-order chromatin structure that is likely to drastically alter its properties.

Acute High-Order Chromatin Modifications Correlate with Enhanced 53BP1 Recruitment *In Vivo*

An obvious role for chromatin-mediated DSB signaling would be to directly attract effectors of the DDR. 53BP1 plays key roles in DSB repair by inhibiting end resection, favoring distal end synapsis, and promoting damaged chromatin mobility (for review, see Panier and Boulton, 2014). 53BP1 interaction with damaged chromatin involves multivalent engagement of several histone modifications such as H2A (or H2AX) lysine 15 ubiquitination and methylation of lysine 20 on histone H4 (Fradet-Turcotte et al., 2013; Wilson et al., 2016). Furthermore, stable 53BP1 association with DSBs may involve a direct interaction with γH2AX (Kleiner et al., 2015; Ward et al., 2003) and can be regulated by acetylation on H4 and H2A (Jacquet et al., 2016; Tang et al., 2013). We generated a genome-wide ChIP-seq map of 53BP1 binding to damaged chromatin *in vivo*. Inspection of 53BP1 distribution around individual DSBs (Figure 7A) or averaged over the 80 DSBs (Figure S6A) revealed a striking ability for 53BP1 to spread within megabase-wide domains. Furthermore, 53BP1 binding profiles appear almost indistinguishable from those of γH2AX and ubiquitin (Figures 7A and S6A), and we observed a very strong correlation between the accumulation of γH2AX, ubiquitin, and 53BP1 within 1 Mb domains surrounding DSBs (Figure S6B). We also observed that 53BP1 is significantly more enriched within a megabase from sites preferentially repaired by HR compared with sites repaired by NHEJ (Figures 7B, 7C, and S6C). Thus, our data suggest that DSBs occurring in active chromatin, which preferentially undergo HR repair, can trigger an acute high-order chromatin signaling that may favor subsequent recruitment of 53BP1 over megabase-sized regions encompassing the break site.

In vivo, 53BP1 was shown to inhibit 5′ end resection in various contexts, including class switch recombination and dysfunctional telomeres fusion (for review, see Panier and Boulton, 2014). Yet our results suggest that 53BP1 binding is stronger at sites that are more prone to be repaired by HR, a process that requires end resection. To clarify this apparent discrepancy, we generated 53BP1 ChIP-seq data in damaged G1- and G2-synchronized cells. Overall, we found that 53BP1 accumulation was much stronger during G1 compared to G2, (Figures 7D and S6D–S6F). Because γH2AX appeared rather similar between both phases of the cell cycle (Figure S6F), this suggests that the striking difference in 53BP1 spreading over megabase-wide domains observed when comparing G1 and G2 cells is unlikely to be due to differences in DSB induction rates. Finally, we confirmed that 53BP1 accumulation is more prominent at DSBs repaired by HR in G1 compared to G2, while signal for NHEJ DSB remained consistently lower regardless of the cell

cycle phase (Figure 7E). Thus, our data revealed that 53BP1 binding is favored at HR-prone DSBs, but mostly in G1, when HR usage is restricted. This suggests that 53BP1 exerts its anti-resection function in G1 only at sites prone to undergo HR, while it may be less critical at DSBs usually repaired by NHEJ.

DISCUSSION

A Standardized Approach to Investigate DSB-Induced Chromatin Changes

Here, we provide the most comprehensive view to date of the chromatin landscape assembled at DSBs in human cells. By analyzing 20 chromatin features that were previously shown to be involved in DSB repair, we were able to substantiate several earlier findings such as H3, H2AZ, and H1 removal, macroH2A incorporation, H4S1 phosphorylation, H4K16 deacetylation, H4K20 monomethylation, and the accumulation of ubiquitin conjugates at sites of damage (Table S2). Interestingly, DSB-induced phosphorylation of histone H4 has only been previously reported in budding yeast and was proposed to promote NHEJ (Cheung et al., 2005; Utley et al., 2005). Break-induced H4 phosphorylation is therefore conserved in higher eukaryotes and spreads over large regions spanning up to 10 kb from the DSB, independently of the repair pathway. This ability to propagate up to 50 nucleosomes away from the break suggests that H4 phosphorylation functions in DDR signaling, potentially as a recruitment platform for DDR proteins.

Here we failed to detect any change reported by others (Table S2) in H3K9me2, H3K9me3, H4K20me2, or H3K56ac. Conversely, we observed a clear loss of H3K79me2 and H2BK120ub, two histone modifications that were found, respectively, unchanged and increased at sites of breaks (Huyen et al., 2004; Moyal et al., 2011; Nakamura et al., 2011). Such discrepancies could arise from the use of different DSB induction methods that can produce chemically different DNA ends, concomitantly trigger other forms of DNA damage, or create DSBs at a given cell cycle stage. More importantly, DSB-inducing agents do not necessarily damage the same regions across the genome, which will strongly influence the chromatin outcome. Indeed, X- and γ-rays produce DSBs randomly throughout the genome, mainly in untranscribed and potentially heterochromatic regions, while AsiSI mostly introduces DSBs within or near genes, either active or inactive, leaving heterochromatin undamaged. This could explain why neocarzinostatin and ionizing radiation, unlike AsiSI-induced DSBs, trigger an increase in H2BK120ub levels (Moyal et al., 2011; Nakamura et al., 2011). Kinetic aspects should also be considered. We performed our ChIP 4 hr after AsiSI activation, a time point representing an "equilibrium" in which events ranging from early cleavage to late repair cohabit within the cell population. However, this may preclude the identification of very transient and/or very late events. Finally, in order to identify the most prominent DSB-induced chromatin features, we purposely limited our large-scale analysis to an averaged snapshot on a large population of asynchronous cells, which may hinder the identification of events occurring at specific cell cycle stages. Yet despite a few discrepancies with the published literature, our study allowed us to highlight major chromatin changes during DSB repair and to associate these changes with a preferred repair pathway. Additional studies in repair-deficient cells should now be conducted in order to establish whether these histone modification changes do indeed depend on HR or NHEJ repair or only on the initial chromatin status. Moreover, systematic mapping of chromatin-modifying enzymes following DNA damage will be required to better understand the establishment of this multiscale chromatin landscape. Investigating if, when, and how this DSB-induced chromatin landscape is reverted following repair in order to maintain epigenome stability also represent exciting follow-up studies.

Histone Crosstalks Involved in Transcription Regulation Are Mobilized in the DSB Response

Importantly, our data fully recapitulate several crosstalks previously identified in the context of chromatin organization and transcriptional regulation. Indeed, our study reveals that upon DSB induction, macroH2A is incorporated (Khurana et al., 2014), and H2BK120 undergoes a switch from ubiquitination to acetylation. MacroH2A can regulate gene expression by stimulating CBP-dependent H2BK120 acetylation (Chen et al., 2014), which precludes H2BK120 ubiquitination. Our findings suggest that this macroH2A-H2BK120ac crosstalk goes beyond transcriptional regulation and may also function following DNA damage and repair. Our work revealed that the transition from ubiquitination to acetylation on H2BK120 may be mediated by the SAGA complex, a well-known complex involved in transcriptional regulation, and that depletion of DUB and HAT subunits of SAGA leads to defective HR and NHEJ, in agreement with a previous study (Ramachandran et al., 2016). Notably, these macroH2A-H2BK120 changes occur independently of the DSB considered (i.e., prone to be repaired by HR or NHEJ), suggesting that they take place before repair reaction is engaged. Given the function of H2BK120ub in nucleosome stabilization (Batta et al., 2011; Fleming et al., 2008), deubiquitinating H2B may loosen nucleosome stability onto DNA, thereby favoring subsequent remodeling and downstream repair events.

We also identified seven additional chromatin changes after DSB induction, including decreases in H3K79me2, H2AZ, and H4K12/K16ac, which specifically occurred at DSBs prone to HR (Figure S7). We also recently demonstrated that H3K4me3 decreases at AsiSI-induced DSBs (Gong et al., 2017). Notably, all these modifications were already found to be coordinated and linked to H2B ubiquitination during transcriptional regulation. For example, H2BK120ub is required for H3K79 methylation (McGinty et al., 2008; Sun and Allis, 2002) and counteracts INO80-dependent H2AZ removal (Segala et al., 2016). Finally, H2B ubiquitination stimulates H3K4 trimethylation (Sun and Allis, 2002; Zhu et al., 2005). Our data indicate that this well-established crosstalk likely also functions at DSBs and that the switch from H2BK120 ubiquitination to acetylation at DSBs may subsequently induce H3K79 and H3K4me3 demethylation, H4 deacetylation, and H2AZ removal by INO80, which itself associates with DSB (Downs et al., 2004; Morrison et al., 2004; van Attikum et al., 2004). Since H2AZ occupancy was found to regulate end resection (Alatwi and Downs, 2015; Gursoy-Yuzugullu et al., 2015; Xu et al., 2012), it is possible that these coordinated

modifications of H2BK120ub-H3K79me2-H2AZ-H4 at HR-prone DSBs contribute to the establishment of a chromatin state competent for resection and/or Rad51 nucleofilament assembly.

Megabase-Scale Chromatin Signaling: A Central Unit in the DSB Response

Chromatin flanking DSBs also undergoes extensive large-scale remodeling, such as γH2AX spreading over megabase-wide domains (Iacovoni et al., 2010; Rogakou et al., 1998; Savic et al., 2009). Here we show that this large-scale remodeling is also accompanied by the accumulation of ubiquitin conjugates, 53BP1 accrual, and the removal of histone H1 from the entire γH2AX domain. 53BP1 is known to be recruited to damaged chromatin by engaging multiple interactions with H2AK15ub and H4K20me2 but also with γH2AX (for review, see Panier and Boulton, 2014). Our data support this proposed mode of recruitment given that: (1) 53BP1 strongly parallels the pattern observed with γH2AX and the FK2 antibody (although the broad specificity of this antibody toward many ubiquitinated substrates precludes a definitive conclusion regarding the nature of the modified protein) and (2) 53BP1 association with DSB is minimal in G2, in agreement with the proposed dilution of H4K20me2 on post-replicative chromatin (Pellegrino et al., 2017). H1, on the other hand, was recently shown to be displaced from sites of DNA damage (Sellou et al., 2016; Strickfaden et al., 2016). Interestingly, RNF8-mediated H1 ubiquitination loosens its interaction with chromatin (Thorslund et al., 2015), providing potential mechanisms for H1 eviction from megabase-wide γH2AX domains. These megabase-wide chromatin modules display well-defined boundaries that may correlate with topologically associated domains (TADs) (Caron et al., 2012; Marnef and Legube, 2017), a basic unit of chromosome folding in 3D. The establishment of these large chromatin changes might therefore be determined by the initial chromosome structure (for review, see Aymard and Legube, 2016; Marnef and Legube, 2017). Such profound changes in entire TADs could regulate the chromatin fiber physical properties such as the mobility of the damaged DNA within the nucleus. In agreement, linker histones strongly affect the global chromatin structure (Fan et al., 2005), and 53BP1 favors long-range motions of DNA ends (Dimitrova et al., 2008).

The Complex Fate of DSBs in Active Transcription Units

DSB repair varies across the genome. Damage in heterochromatin is largely repaired by HR in G2 by a specific pathway requiring 53BP1-dependent chromatin relaxation, followed by its relocation at the periphery of IR-induced foci (Kakarougkas et al., 2013; Noon et al., 2010). In euchromatin, only transcriptionally active genes, when damaged, are prone to HR repair in G2 (Aymard et al., 2014; this study). Importantly, damaged active genes are refractory to repair in G1, where they instead persist and cluster together within foci (Aymard et al., 2017). Here we found that megabase-size chromatin modifications are more prominent at sites prone to be repaired by HR, e.g., DSBs occurring within active chromatin domains. Such an acute signaling could be indicative of a specific DDR pathway mobilized upon damage in active regions of the genome. Additionally, we found that these DSBs also display enhanced binding of 53BP1, specifically during G1. We did not observe specific 53BP1 accumulation or redistribution at HR-prone DSBs during G2 in our study, suggesting that 53BP1 is likely dispensable to promote HR in the euchromatic, relaxed fraction of the genome. 53BP1 may interact with DSBs to inhibit extensive end processing in G1 and avoid the use of deleterious pathways such as alt-NHEJ during times when canonical HR is not available (Biehs et al., 2017).

In summary, we provide here the first comprehensive depiction of the set of histone modifications induced upon DSB associating with HR and NHEJ repair and report a thorough description of HR-competent chromatin, which is strongly linked with transcriptional activity. Active genes emerge as particularly fragile loci that frequently experience DNA double-strand breakage (for review, see Marnef et al., 2017) and represent translocation hotspots. Characterizing the local and large-scale chromatin structures that assemble at these specific damaged loci represents the first step to understanding how chromatin mobility may change following damage to promote DSB clustering and homology search and, in some instance, lead to translocations.

STAR★METHODS

Detailed methods are provided in the online version of this paper and include the following:

- KEY RESOURCES TABLE
- CONTACT FOR REAGENT AND RESOURCE SHARING
- EXPERIMENTAL MODEL AND SUBJECT DETAILS
  - Chemicals
  - Cell Culture and Cell Lines
- METHOD DETAILS
  - Cell treatments
  - Establishment of cell line expressing SUPT7L from the AAVS1 safe harbor and TAP Purification of native human SAGA HAT/DUB complex
  - *In vitro* histone acetylation assay
  - *In vitro* histone de-ubiquitination assay
  - RT-qPCR
  - Cas9/mClover-LMNA1 homologous recombination assay
  - NHEJ assay
  - Cell cycle analysis
  - ChIP
  - BLESS
- QUANTIFICATION AND STATISTICAL ANALYSIS
  - ChIP-seq data processing
  - BLESS data processing
  - Descriptive statistics using box-plot representation
  - Statistical analysis and representations
  - Averaged ChIP-seq profiles
  - Generation of random positions
  - Determination of HR-prone and NHEJ-prone DSBs
- DATA AND SOFTWARE AVAILABILITY

SUPPLEMENTAL INFORMATION

Supplemental Information includes seven figures and two tables and can be found with this article online at https://doi.org/10.1016/j.molcel.2018.08.020.

ACKNOWLEDGMENTS

We thank the GeneCore facility of EMBL for high-throughput sequencing. We thank K.M. Miller (University of Texas) for critical reading of the manuscript and J.-Y. Masson/G. Dellaire for reagents and advice for the Clover/HR assay. Funding was provided by Polish National Science Centre (2011/02/A/NZ2/00014 to K.G., 2016/21/B/ST6/01471 to N.D., and 2015/17/D/NZ2/03711 to M.S.) and Foundation for Polish Science (TEAM to K.G.). Funding to M.R., B.F., and N.D. was provided by NIH (5 R01 GM 112131). J.C. acknowledges funding support from the Canadian Institutes of Health Research (FND-143314) and holds the Canada Research Chair in Chromatin Biology and Molecular Epigenetics. M.A. was supported by the Fondation pour la Recherche Médicale (FRM). Funding in G.L.'s laboratory was provided by grants from the European Research Council (ERC-2014-CoG 647344), Agence Nationale pour la Recherche (ANR-14-CE10-0002-01 and ANR-13-BSV8-0013), the Institut National contre le Cancer (INCA), and the Ligue Nationale contre le Cancer (LNCC). T.C. is an INSERM researcher.

AUTHOR CONTRIBUTIONS

T.C., C.A., and F.A. performed ChIP experiments. A.L. performed the SAGA complex purification, enzymatic assays, and DSB repair assays under the supervision of J.C. V.R., M.A., and J.S.I. performed bioinformatic analyses of ChIP and BLESS datasets. A.B., M.S., and K.G. performed BLESS experiments. M.R., B.F. and N.D. performed the comparison with Hi-C data. T.C. and G.L. conceived, supervised, and analyzed experiments. T.C. and G.L. wrote the manuscript. All authors commented on and edited the manuscript.

DECLARATION OF INTERESTS

The authors declare no competing interests.

Received: June 26, 2017
Revised: July 13, 2018
Accepted: August 13, 2018
Published: September 27, 2018

REFERENCES

Alatwi, H.E., and Downs, J.A. (2015). Removal of H2A.Z by INO80 promotes homologous recombination. EMBO Rep. *16*, 986–994.

Aymard, F., and Legube, G. (2016). A TAD closer to ATM. Mol. Cell. Oncol. *3*, e1134411.

Aymard, F., Bugler, B., Schmidt, C.K., Guillou, E., Caron, P., Briois, S., Iacovoni, J.S., Daburon, V., Miller, K.M., Jackson, S.P., and Legube, G. (2014). Transcriptionally active chromatin recruits homologous recombination at DNA double-strand breaks. Nat. Struct. Mol. Biol. *21*, 366–374.

Aymard, F., Aguirrebengoa, M., Guillou, E., Javierre, B.M., Bugler, B., Arnould, C., Rocher, V., Iacovoni, J.S., Biernacka, A., Skrzypczak, M., et al. (2017). Genome-wide mapping of long-range contacts unveils clustering of DNA double-strand breaks at damaged active genes. Nat. Struct. Mol. Biol. *24*, 353–361.

Batta, K., Zhang, Z., Yen, K., Goffman, D.B., and Pugh, B.F. (2011). Genome-wide function of H2B ubiquitylation in promoter and genic regions. Genes Dev. *25*, 2254–2265.

Bennett, G., and Peterson, C.L. (2015). SWI/SNF recruitment to a DNA double-strand break by the NuA4 and Gcn5 histone acetyltransferases. DNA Repair (Amst.) *30*, 38–45.

Biehs, R., Steinlage, M., Barton, O., Juhasz, S., Kunzel, J., Spies, J., Shibata, A., Jeggo, P.A., and Lobrich, M. (2017). DNA Double-Strand Break Resection Occurs during Non-homologous End Joining in G1 but Is Distinct from Resection during Homologous Recombination. Mol. Cell *65*, 671–684 e675.

Caron, P., Aymard, F., Iacovoni, J.S., Briois, S., Canitrot, Y., Bugler, B., Massip, L., Losada, A., and Legube, G. (2012). Cohesin protects genes against γH2AX Induced by DNA double-strand breaks. PLoS Genet. *8*, e1002460.

Caron, P., Choudjaye, J., Clouaire, T., Bugler, B., Daburon, V., Aguirrebengoa, M., Mangeat, T., Iacovoni, J.S., Álvarez-Quilón, A., Cortés-Ledesma, F., and Legube, G. (2015). Non-redundant Functions of ATM and DNA-PKcs in Response to DNA Double-Strand Breaks. Cell Rep. *13*, 1598–1609.

Chen, H., Ruiz, P.D., Novikov, L., Casill, A.D., Park, J.W., and Gamble, M.J. (2014). MacroH2A1.1 and PARP-1 cooperate to regulate transcription by promoting CBP-mediated H2B acetylation. Nat. Struct. Mol. Biol. *21*, 981–989.

Cheung, W.L., Turner, F.B., Krishnamoorthy, T., Wolner, B., Ahn, S.H., Foley, M., Dorsey, J.A., Peterson, C.L., Berger, S.L., and Allis, C.D. (2005). Phosphorylation of histone H4 serine 1 during DNA damage requires casein kinase II in S. cerevisiae. Curr. Biol. *15*, 656–660.

Clouaire, T., and Legube, G. (2015). DNA double strand break repair pathway choice: a chromatin based decision? Nucleus *6*, 107–113.

Cohen, S., Puget, N., Lin, Y.L., Clouaire, T., Aguirrebengoa, M., Rocher, V., Pasero, P., Canitrot, Y., and Legube, G. (2018). Senataxin resolves RNA:DNA hybrids forming at DNA double-strand breaks to prevent translocations. Nat. Commun. *9*, 533.

Crosetto, N., Mitra, A., Silva, M.J., Bienko, M., Dojer, N., Wang, Q., Karaca, E., Chiarle, R., Skrzypczak, M., Ginalski, K., et al. (2013). Nucleotide-resolution DNA double-strand break mapping by next-generation sequencing. Nat. Methods *10*, 361–365.

Dalvai, M., Loehr, J., Jacquet, K., Huard, C.C., Roques, C., Herst, P., Côté, J., and Doyon, Y. (2015). A Scalable Genome-Editing-Based Approach for Mapping Multiprotein Complexes in Human Cells. Cell Rep. *13*, 621–633.

Deplus, R., Blanchon, L., Rajavelu, A., Boukaba, A., Defrance, M., Luciani, J., Rothé, F., Dedeurwaerder, S., Denis, H., Brinkman, A.B., et al. (2014). Regulation of DNA methylation patterns by CK2-mediated phosphorylation of Dnmt3a. Cell Rep. *8*, 743–753.

Dimitrova, N., Chen, Y.C., Spector, D.L., and de Lange, T. (2008). 53BP1 promotes non-homologous end joining of telomeres by increasing chromatin mobility. Nature *456*, 524–528.

Dobbin, M.M., Madabhushi, R., Pan, L., Chen, Y., Kim, D., Gao, J., Ahanonu, B., Pao, P.C., Qiu, Y., Zhao, Y., and Tsai, L.H. (2013). SIRT1 collaborates with ATM and HDAC1 to maintain genomic stability in neurons. Nat. Neurosci. *16*, 1008–1015.

Downs, J.A., Allard, S., Jobin-Robitaille, O., Javaheri, A., Auger, A., Bouchard, N., Kron, S.J., Jackson, S.P., and Côté, J. (2004). Binding of chromatin-modifying activities to phosphorylated histone H2A at DNA damage sites. Mol. Cell *16*, 979–990.

Doyon, Y., and Côté, J. (2016). Preparation and Analysis of Native Chromatin-Modifying Complexes. Methods Enzymol. *573*, 303–318.

Engel, M., Eggert, C., Kaplick, P.M., Eder, M., Röh, S., Tietze, L., Namendorf, C., Arloth, J., Weber, P., Rex-Haffner, M., et al. (2018). The Role of m6A/m-RNA Methylation in Stress Response Regulation. Neuron *99*, 389–403.e9.

Fan, Y., Nikitina, T., Zhao, J., Fleury, T.J., Bhattacharyya, R., Bouhassira, E.E., Stein, A., Woodcock, C.L., and Skoultchi, A.I. (2005). Histone H1 depletion in mammals alters global chromatin structure but causes specific changes in gene regulation. Cell *123*, 1199–1212.

Fleming, A.B., Kao, C.F., Hillyer, C., Pikaart, M., and Osley, M.A. (2008). H2B ubiquitylation plays a role in nucleosome dynamics during transcription elongation. Mol. Cell *31*, 57–66.

Fradet-Turcotte, A., Canny, M.D., Escribano-Díaz, C., Orthwein, A., Leung, C.C., Huang, H., Landry, M.C., Kitevski-LeBlanc, J., Noordermeer, S.M., Sicheri, F., and Durocher, D. (2013). 53BP1 is a reader of the DNA-damage-induced H2A Lys 15 ubiquitin mark. Nature *499*, 50–54.

Gong, F., Chiu, L.Y., Cox, B., Aymard, F., Clouaire, T., Leung, J.W., Cammarata, M., Perez, M., Agarwal, P., Brodbelt, J.S., et al. (2015). Screen identifies bromodomain protein ZMYND8 in chromatin recognition of transcription-associated DNA damage that promotes homologous recombination. Genes Dev. *29*, 197–211.

Gong, F., Clouaire, T., Aguirrebengoa, M., Legube, G., and Miller, K.M. (2017). Histone demethylase KDM5A regulates the ZMYND8-NuRD chromatin remodeler to promote DNA repair. J. Cell Biol. *216*, 1959–1974.

Gursoy-Yuzugullu, O., Ayrapetov, M.K., and Price, B.D. (2015). Histone chaperone Anp32e removes H2A.Z from DNA double-strand breaks and promotes nucleosome reorganization and DNA repair. Proc. Natl. Acad. Sci. USA *112*, 7507–7512.

Helmlinger, D., and Tora, L. (2017). Sharing the SAGA. Trends Biochem. Sci. *42*, 850–861.

Hustedt, N., and Durocher, D. (2016). The control of DNA repair by the cell cycle. Nat. Cell Biol. *19*, 1–9.

Huyen, Y., Zgheib, O., Ditullio, R.A., Jr., Gorgoulis, V.G., Zacharatos, P., Petty, T.J., Sheston, E.A., Mellert, H.S., Stavridi, E.S., and Halazonetis, T.D. (2004). Methylated lysine 79 of histone H3 targets 53BP1 to DNA double-strand breaks. Nature *432*, 406–411.

Iacovoni, J.S., Caron, P., Lassadi, I., Nicolas, E., Massip, L., Trouche, D., and Legube, G. (2010). High-resolution profiling of gammaH2AX around DNA double strand breaks in the mammalian genome. EMBO J. *29*, 1446–1457.

Iannelli, F., Galbiati, A., Capozzo, I., Nguyen, Q., Magnuson, B., Michelini, F., D'Alessandro, G., Cabrini, M., Roncador, M., Francia, S., et al. (2017). A damaged genome's transcriptional landscape through multilayered expression profiling around in situ-mapped DNA double-strand breaks. Nat. Commun. *8*, 15656.

Jacquet, K., Fradet-Turcotte, A., Avvakumov, N., Lambert, J.P., Roques, C., Pandita, R.K., Paquet, E., Herst, P., Gingras, A.C., Pandita, T.K., et al. (2016). The TIP60 Complex Regulates Bivalent Chromatin Recognition by 53BP1 through Direct H4K20me Binding and H2AK15 Acetylation. Mol. Cell *62*, 409–421.

Kakarougkas, A., Ismail, A., Klement, K., Goodarzi, A.A., Conrad, S., Freire, R., Shibata, A., Lobrich, M., and Jeggo, P.A. (2013). Opposing roles for 53BP1 during homologous recombination. Nucleic Acids Res. *41*, 9719–9731.

Khurana, S., Kruhlak, M.J., Kim, J., Tran, A.D., Liu, J., Nyswaner, K., Shi, L., Jailwala, P., Sung, M.H., Hakim, O., and Oberdoerffer, P. (2014). A macrohistone variant links dynamic chromatin compaction to BRCA1-dependent genome maintenance. Cell Rep. *8*, 1049–1062.

Kleiner, R.E., Verma, P., Molloy, K.R., Chait, B.T., and Kapoor, T.M. (2015). Chemical proteomics reveals a γH2AX-53BP1 interaction in the DNA damage response. Nat. Chem. Biol. *11*, 807–814.

Lee, H.S., Park, J.H., Kim, S.J., Kwon, S.J., and Kwon, J. (2010). A cooperative activation loop among SWI/SNF, gamma-H2AX and H3 acetylation for DNA double-strand break repair. EMBO J. *29*, 1434–1445.

Marnef, A., and Legube, G. (2017). Organizing DNA repair in the nucleus: DSBs hit the road. Curr. Opin. Cell Biol. *46*, 1–8.

Marnef, A., Cohen, S., and Legube, G. (2017). Transcription-Coupled DNA Double-Strand Break Repair: Active Genes Need Special Care. J. Mol. Biol. *429*, 1277–1288.

McGinty, R.K., Kim, J., Chatterjee, C., Roeder, R.G., and Muir, T.W. (2008). Chemically ubiquitylated histone H2B stimulates hDot1L-mediated intranucleosomal methylation. Nature *453*, 812–816.

Miller, K.M., Tjeertes, J.V., Coates, J., Legube, G., Polo, S.E., Britton, S., and Jackson, S.P. (2010). Human HDAC1 and HDAC2 function in the DNA-damage response to promote DNA nonhomologous end-joining. Nat. Struct. Mol. Biol. *17*, 1144–1151.

Mitra, A., Skrzypczak, M., Ginalski, K., and Rowicka, M. (2015). Strategies for achieving high sequencing accuracy for low diversity samples and avoiding sample bleeding using illumina platform. PLoS ONE *10*, e0120520.

Mladenov, E., Magin, S., Soni, A., and Iliakis, G. (2016). DNA double-strand-break repair in higher eukaryotes and its role in genomic instability and cancer: Cell cycle and proliferation-dependent regulation. Semin. Cancer Biol. *37-38*, 51–64.

Morrison, A.J., Highland, J., Krogan, N.J., Arbel-Eden, A., Greenblatt, J.F., Haber, J.E., and Shen, X. (2004). INO80 and gamma-H2AX interaction links ATP-dependent chromatin remodeling to DNA damage repair. Cell *119*, 767–775.

Moyal, L., Lerenthal, Y., Gana-Weisz, M., Mass, G., So, S., Wang, S.Y., Eppink, B., Chung, Y.M., Shalev, G., Shema, E., et al. (2011). Requirement of ATM-dependent monoubiquitylation of histone H2B for timely repair of DNA double-strand breaks. Mol. Cell *41*, 529–542.

Nakamura, K., Kato, A., Kobayashi, J., Yanagihara, H., Sakamoto, S., Oliveira, D.V., Shimada, M., Tauchi, H., Suzuki, H., Tashiro, S., et al. (2011). Regulation of homologous recombination by RNF20-dependent H2B ubiquitination. Mol. Cell *41*, 515–528.

Noon, A.T., Shibata, A., Rief, N., Löbrich, M., Stewart, G.S., Jeggo, P.A., and Goodarzi, A.A. (2010). 53BP1-dependent robust localized KAP-1 phosphorylation is essential for heterochromatic DNA double-strand break repair. Nat. Cell Biol. *12*, 177–184.

Ogiwara, H., Ui, A., Otsuka, A., Satoh, H., Yokomi, I., Nakajima, S., Yasui, A., Yokota, J., and Kohno, T. (2011). Histone acetylation by CBP and p300 at double-strand break sites facilitates SWI/SNF chromatin remodeling and the recruitment of non-homologous end joining factors. Oncogene *30*, 2135–2146.

Panier, S., and Boulton, S.J. (2014). Double-strand break repair: 53BP1 comes into focus. Nat. Rev. Mol. Cell Biol. *15*, 7–18.

Pauty, J., Couturier, A.M., Rodrigue, A., Caron, M.C., Coulombe, Y., Dellaire, G., and Masson, J.Y. (2017). Cancer-causing mutations in the tumor suppressor PALB2 reveal a novel cancer mechanism using a hidden nuclear export signal in the WD40 repeat motif. Nucleic Acids Res. *45*, 2644–2657.

Pellegrino, S., Michelena, J., Teloni, F., Imhof, R., and Altmeyer, M. (2017). Replication-Coupled Dilution of H4K20me2 Guides 53BP1 to Pre-replicative Chromatin. Cell Rep. *19*, 1819–1831.

Pinder, J., Salsman, J., and Dellaire, G. (2015). Nuclear domain 'knock-in' screen for the evaluation and identification of small molecule enhancers of CRISPR-based genome editing. Nucleic Acids Res. *43*, 9379–9392.

Ramachandran, S., Haddad, D., Li, C., Le, M.X., Ling, A.K., So, C.C., Nepal, R.M., Gommerman, J.L., Yu, K., Ketela, T., et al. (2016). The SAGA Deubiquitination Module Promotes DNA Repair and Class Switch Recombination through ATM and DNAPK-Mediated γH2AX Formation. Cell Rep. *15*, 1554–1565.

Rao, S.S., Huntley, M.H., Durand, N.C., Stamenova, E.K., Bochkov, I.D., Robinson, J.T., Sanborn, A.L., Machol, I., Omer, A.D., Lander, E.S., and Aiden, E.L. (2014). A 3D map of the human genome at kilobase resolution reveals principles of chromatin looping. Cell *159*, 1665–1680.

Rogakou, E.P., Pilch, D.R., Orr, A.H., Ivanova, V.S., and Bonner, W.M. (1998). DNA double-stranded breaks induce histone H2AX phosphorylation on serine 139. J. Biol. Chem. *273*, 5858–5868.

Ropars, V., Drevet, P., Legrand, P., Baconnais, S., Amram, J., Faure, G., Márquez, J.A., Piétrement, O., Guerois, R., Callebaut, I., et al. (2011). Structural characterization of filaments formed by human Xrcc4-Cernunnos/XLF complex involved in nonhomologous DNA end-joining. Proc. Natl. Acad. Sci. USA *108*, 12663–12668.

Savic, V., Yin, B., Maas, N.L., Bredemeyer, A.L., Carpenter, A.C., Helmink, B.A., Yang-Iott, K.S., Sleckman, B.P., and Bassing, C.H. (2009). Formation of dynamic gamma-H2AX domains along broken DNA strands is distinctly regulated by ATM and MDC1 and dependent upon H2AX densities in chromatin. Mol. Cell *34*, 298–310.

Schipler, A., and Iliakis, G. (2013). DNA double-strand-break complexity levels and their possible contributions to the probability for error-prone processing and repair pathway choice. Nucleic Acids Res. *41*, 7589–7605.

Schwertman, P., Bekker-Jensen, S., and Mailand, N. (2016). Regulation of DNA double-strand break repair by ubiquitin and ubiquitin-like modifiers. Nat. Rev. Mol. Cell Biol. *17*, 379–394.

Segala, G., Bennesch, M.A., Pandey, D.P., Hulo, N., and Picard, D. (2016). Monoubiquitination of Histone H2B Blocks Eviction of Histone Variant H2A.Z from Inducible Enhancers. Mol. Cell *64*, 334–346.

Sellou, H., Lebeaupin, T., Chapuis, C., Smith, R., Hegele, A., Singh, H.R., Kozlowski, M., Bultmann, S., Ladurner, A.G., Timinszky, G., and Huet, S. (2016). The poly(ADP-ribose)-dependent chromatin remodeler Alc1 induces local chromatin relaxation upon DNA damage. Mol. Biol. Cell *27*, 3791–3799.

Soshnev, A.A., Josefowicz, S.Z., and Allis, C.D. (2016). Greater Than the Sum of Parts: Complexity of the Dynamic Epigenome. Mol. Cell *62*, 681–694.

Strickfaden, H., McDonald, D., Kruhlak, M.J., Haince, J.F., Th'ng, J.P., Rouleau, M., Ishibashi, T., Corry, G.N., Ausio, J., Underhill, D.A., et al. (2016). Poly(ADP-ribosyl)ation-dependent Transient Chromatin Decondensation and Histone Displacement following Laser Microirradiation. J. Biol. Chem. *291*, 1789–1802.

Sun, Z.W., and Allis, C.D. (2002). Ubiquitination of histone H2B regulates H3 methylation and gene silencing in yeast. Nature *418*, 104–108.

Talbert, P.B., and Henikoff, S. (2017). Histone variants on the move: substrates for chromatin dynamics. Nat. Rev. Mol. Cell Biol. *18*, 115–126.

Tang, J., Cho, N.W., Cui, G., Manion, E.M., Shanbhag, N.M., Botuyan, M.V., Mer, G., and Greenberg, R.A. (2013). Acetylation limits 53BP1 association with damaged chromatin to promote homologous recombination. Nat. Struct. Mol. Biol. *20*, 317–325.

Thorslund, T., Ripplinger, A., Hoffmann, S., Wild, T., Uckelmann, M., Villumsen, B., Narita, T., Sixma, T.K., Choudhary, C., Bekker-Jensen, S., and Mailand, N. (2015). Histone H1 couples initiation and amplification of ubiquitin signalling after DNA damage. Nature *527*, 389–393.

Toiber, D., Erdel, F., Bouazoune, K., Silberman, D.M., Zhong, L., Mulligan, P., Sebastian, C., Cosentino, C., Martinez-Pastor, B., Giacosa, S., et al. (2013). SIRT6 recruits SNF2H to DNA break sites, preventing genomic instability through chromatin remodeling. Mol. Cell *51*, 454–468.

Utley, R.T., Lacoste, N., Jobin-Robitaille, O., Allard, S., and Côté, J. (2005). Regulation of NuA4 histone acetyltransferase activity in transcription and DNA repair by phosphorylation of histone H4. Mol. Cell. Biol. *25*, 8179–8190.

van Attikum, H., Fritsch, O., Hohn, B., and Gasser, S.M. (2004). Recruitment of the INO80 complex by H2A phosphorylation links ATP-dependent chromatin remodeling with DNA double-strand break repair. Cell *119*, 777–788.

Ward, I.M., Minn, K., Jorda, K.G., and Chen, J. (2003). Accumulation of checkpoint protein 53BP1 at DNA breaks involves its binding to phosphorylated histone H2AX. J. Biol. Chem. *278*, 19579–19582.

Wilson, M.D., Benlekbir, S., Fradet-Turcotte, A., Sherker, A., Julien, J.P., McEwan, A., Noordermeer, S.M., Sicheri, F., Rubinstein, J.L., and Durocher, D. (2016). The structural basis of modified nucleosome recognition by 53BP1. Nature *536*, 100–103.

Xu, Y., Ayrapetov, M.K., Xu, C., Gursoy-Yuzugullu, O., Hu, Y., and Price, B.D. (2012). Histone H2A.Z controls a critical chromatin remodeling step required for DNA double-strand break repair. Mol. Cell *48*, 723–733.

Zhu, B., Zheng, Y., Pham, A.D., Mandal, S.S., Erdjument-Bromage, H., Tempst, P., and Reinberg, D. (2005). Monoubiquitination of human histone H2B: the factors involved and their roles in HOX gene regulation. Mol. Cell *20*, 601–611.

STAR★METHODS

KEY RESOURCES TABLE

REAGENT or RESOURCE	SOURCE	IDENTIFIER
Antibodies		
Anti-H4	Abcam	Cat# ab7311, RRID:AB_305837
Anti-H2B	Abcam	Cat# ab1790, RRID:AB_302612
Anti-H3	Abcam	Cat# ab1791, RRID:AB_302613
Anti-H2AZ	Abcam	Cat# ab4174, RRID:AB_304345
Anti-H1	Abcam	Cat# ab17677, RRID:AB_2117984
Anti-macroH2A1	Millipore	Cat# 07-219, RRID:AB_310439
Anti-H2AZac	Abcam	Cat# ab18262, RRID:AB_873820
Anti-H3K79me2	Active Motif	Cat# 39143, RRID:AB_2561018
Anti-H4K20me1	Active Motif	Cat# 39727, RRID:AB_2615074
Anti-H3K9me2	Abcam	Cat# ab1220, RRID:AB_449854
Anti-H3K9me3	Abcam	Cat# ab8898, RRID:AB_306848
Anti-H3K4me2	Millipore	Cat# 07-030, RRID:AB_10099880
Anti-H3K36me2	Abcam	Cat# ab9049, RRID:AB_1280939
Anti-H3K36me3	Abcam	Cat# ab9050, RRID:AB_306966
Anti-H4K12ac	Abcam	Cat# ab46983, RRID:AB_873859
Anti-H4K16ac	Millipore	Cat# 07-329, RRID:AB_310525
Anti-H3K56ac	Abcam	Cat# ab76307, RRID:AB_1523762
Anti-H4S1P	Novus	Cat# NB21-2000, RRID:AB_11019163
Anti-H4K20me2	Abcam	Cat# ab9052, RRID:AB_1951942
Anti-H2BK120Ub	Cell Signaling	Cat# 5546, RRID:AB_106934
Anti-H2BK120ac	Millipore	Cat# 07-564, RRID:AB_11213734
Anti-Ubiquitinylated proteins	Millipore	Cat# 04-263, RRID:AB_612093
Anti-γH2AX	Abcam	Cat# ab81299, RRID:AB_1640564
Anti-XRCC4	Abcam	Cat# ab145, RRID:AB_301278
Anti-RAD51	Santa Cruz	Cat# H-92, RRID:AB_2253533
Anti-53BP1	Novus	Cat# NB100-305, RRID:AB_10001695
Anti-DNA Ligase IV	Genetex	Cat# GTX55592
Chemicals, Peptides, and Recombinant Proteins		
(Z)-4-Hydroxytamoxifen	Sigma	Cat# H7904
Thymidine	Sigma	Cat# T1895
Critical Commercial Assays		
Quick-RNA MicroPrep kit	Zymo Research	Cat# R1050
qScript cDNA synthesis kit	Quanta Bio	Cat# 95047-100
Lipofectamine RNAiMAX	Invitrogen	Cat# 13778075
SE. Cell Line 4D-Nucleofector X Kit	Lonza	Cat# V4XC1012
Deposited Data		
Raw data (ChIP-seq and BLESS)	This paper	ArrayExpress: E-MTAB-5817
RAD51 and XRCC4 ChIP-seq	Aymard et al., 2014	ArrayExpress E-MTAB-1241
RNA polymerase II S2P ChIP-seq	Cohen et al., 2018	ArrayExpress E-MTAB-6318
MethylCap-seq	Deplus et al., 2014	GEO GSE26810

(*Continued on next page*)

Continued

REAGENT or RESOURCE	SOURCE	IDENTIFIER
**Experimental Models: Cell Lines**		
DIvA cell	Iacovoni et al., 2010	N/A
U20S-ISceI GFP-RFP (NHEJ)	Jacquet et al., 2016	N/A
3xFlag-Twin-strep-tagged SUPT7L K562	Dalvai et al., 2015	N/A
U2OS	N/A	ATCC HTB-96, RRID:CVCL_0042
**Oligonucleotides**		
HR-DSB1 for ChIP-qPCR<br>FW GATTGGCTATGGGTGTGGAC<br>REV CATCCTTGCAAACCAGTCCT	Aymard et al., 2014	N/A
HR-DSB2 for ChIP-qPCR<br>FW CCGCCAGAAAGTTTCCTAGA<br>REV CTCACCCTTGCAGCACTTG	Aymard et al., 2014	N/A
NHEJ-DSB for ChIP-qPCR<br>FW TGCCGGTCTCCTAGAAGTTG<br>REV GCGCTTGATTTCCCTGAGT	Aymard et al., 2014	N/A
ACTB for ChIP-qPCR<br>FW AGCCGGGCTCTTGCCAAT<br>REV AGTTAGCGCCCAAAGGACCA	This paper	N/A
TAF12 for ChIP-qPCR<br>FW GCTGAGACGAACGCTTCACT<br>REV CCTTCGAACACTGACCCACT	This paper	N/A
siRNA siSUPT7L-46 CUACUAGACCCAACAGAAA<br>[dT] [dT] UUUCUGUUGGGUCUAGUAG[dT] [dT]	This paper	N/A
siRNA siSUPT7L-47 CUAUCACAGUUACAUGC<br>UA[dT] [dT]<br>UAGCAUGUAACUGUGAUAG[dT] [dT]	This paper	N/A
siRNA siKAT2B (PCAF) CUCUAAUCCUCACUCA<br>UUU[dT] [dT]<br>AAAUGAGUGAGGAUUAGAG[dT] [dT]	This paper	N/A
siRNA siKAT2A (GCN5) GCUACUACGUGACCC<br>GGAA[dT] [dT]<br>UUCCGGGUCACGUAGUAGC[dT] [dT]	This paper	N/A
**Recombinant DNA**		
pX330-LMNAgRNA1	Pauty et al., 2017	N/A
pCR2.1-CloverLMNAdonor	Pinder et al., 2015	N/A
piRFP670-N1	Pinder et al., 2015	N/A
AVS1_Puro_PGK1_3xFLAG_Twin_Strep	Dalvai et al., 2015	Addgene #68375
**Software and Algorithms**		
FastQC	N/A	https://www.bioinformatics.babraham.ac.uk/projects/fastqc
Bwa	N/A	http://bio-bwa.sourceforge.net
Samtools	N/A	http://www.htslib.org/
deepTools	N/A	http://deeptools.readthedocs.io
R	N/A	https://www.r-project.org

CONTACT FOR REAGENT AND RESOURCE SHARING

Further information and requests for resources and reagents should be directed to and will be fulfilled by the Lead Contact, Gaëlle Legube (gaelle.legube@univ-tlse3.fr). DIvA cells are subjected to an MTA, to be signed with the CNRS.

EXPERIMENTAL MODEL AND SUBJECT DETAILS

Chemicals

4-hydroxytamoxifen (4OHT) was purchased form Sigma (Sigma; H7904). 4OHT is reconstituted in DMSO at a final concentration of 10 mM, and stored at −20°C. 4OHT was further used at a final concentration of 300 nM to induce DSB in DIvA cells. Thymidine is purchased from Sigma (T1895), reconstituted in PBS at 100 mM and filter sterilized immediately before use.

Cell Culture and Cell Lines

DIvA (AsiSI-ER-U20S) cells were cultured in Dulbecco's modified Eagle's medium (DMEM) supplemented with antibiotics, 10% FCS (Invitrogen) with 1 μg/mL puromycin at 37°C under a humidified atmosphere with 5% CO2. K562 cells stably expressing near physiological levels of 3xFLAG-Twin-Strep-tagged SUPT7L (Dalvai et al., 2015; Doyon and Côté, 2016) were cultured in RPMI medium supplemented with 0.5 μg/mL puromycin.

METHOD DETAILS

Cell treatments

For AsiSI-dependent DSB induction, cells were treated with 300 nM 4OHT for 4 hr. For synchronization in G1 and G2, cells were incubated with 2 mM thymidine for 18 hr, released for 12 hr and subjected to the second thymidine treatment for 18 hr. G1 and G2 cells were treated with 4OHT, respectively, at 11 and 6 hr following thymidine release and harvested 4 hr later.

Establishment of cell line expressing SUPT7L from the AAVS1 safe harbor and TAP Purification of native human SAGA HAT/DUB complex

K562 cells stably expressing near physiological levels of 3xFLAG-Twin-Strep-tagged SUPT7L was established as described previously (Dalvai et al., 2015; Doyon and Côté, 2016). Briefly, SUPT7L cDNA was cloned into AAVS1_Puro_PGK1_3xFLAG_Twin_Strep (Addgene #68375). The cassette is integrated at the AAVS1 locus after DSB induction and recombination targeted by co-transfection with ZFN expression plasmid. Two hundred thousand cells were transfected with 400 ng of ZFN expression vector and 4 μg of donor constructs. Selection and cloning were performed in RPMI medium supplemented with 0.5 μg/mL puromycin starting at 2 to 3 days post transfection. Clones were obtained by limiting dilution and expanded before harvest for western blot analysis.

Native SAGA complex was purified from 3L of the 3xFlag-Twin-strep-tagged SUPT7L K562 cells as described in (Doyon and Côté, 2016). Nuclear extracts were prepared following standard procedures and pre-cleared with CL6B Sepharose beads. FLAG immunoprecipitations with anti-FLAG agarose affinity gel (Sigma M2) were performed followed by elution with 3xFLAG peptide (200 μg/mL from Sigma in the following buffer: 20 mM HEPES pH 7.5, 150 mM KCl, 0.1 mM EDTA, 10% glycerol, 0.1% Tween20, 1 mM DTT and supplemented with proteases, deacetylases, and phosphatase inhibitors), followed by Strep immunoprecipitation with Strep-Tactin Sepharose beads (IBA) and elution with 5 mM D-biotin in the same buffer used for Flag elution. Mass spec analysis confirmed the co-purification of all bona fide subunits of the human SAGA complex.

In vitro histone acetylation assay

Histone acetyltransferase assays were performed as described previously with minor modifications using the purified complex (Jacquet et al., 2016). Briefly, 100 ng of the recombinant H2B was incubated in a 15 μL reaction containing 50 mM Tris-HCl pH 8.0, 10% glycerol, 1 mM EDTA, 1 mM DTT, 1 mM PMSF, 10 mM sodium butyrate 0.15 mM unlabeled Acetyl-CoA (Sigma) with or without the purified complex for 30 min at 30°C. Samples were analyzed by western blot with indicated antibodies.

In vitro histone de-ubiquitination assay

DUB enzymatic activity was assayed by pre-incubating native nucleosomes purified from HeLa cells in buffer containing 50 mM Tris-HCl pH 7.5, 125 mM NaCl, 1 mM DTT, 1 mM $MgCl_2$, 1 mM EDTA, and protease inhibitor cocktail at 30°C for 20 min before adding purified SAGA complex. Reaction were incubated at 30°C throughout the time course and terminated by adding SDS-PAGE sample loading buffer following by western blot analysis using indicated antibodies.

RT-qPCR

For measuring siRNA-mediated depletion total RNA was extracted using the Quick-RNA MicroPrep kit (Zymo Research). One microgram of RNA was reverse transcribed using qScript cDNA synthesis kit (Quanta biosciences) according to the manufacturers' protocols. Samples were then subjected to quantitative PCR (qPCR) using Lightcycler (Roche). The relative abundance of target mRNA was calculated according to the ΔΔ cycle threshold method (ΔΔCt). mRNA expression levels of the housekeeping gene 36B4 gene (also called ribosomal phosphoprotein P0, RPLP0) were used as an internal control to normalize each qPCR reaction. The relative expression levels were calculated as fold enrichment of treated cells over the control cells. Experiments were performed as independent biological triplicates and data are presented as mean ± SD.

Cas9/mClover-LMNA1 homologous recombination assay

The pCR2.1-CloverLMNAdonor, pX330-LMNAgRNA1 and piRFP670-N1 plasmids were previously described (Pinder et al., 2015). The pX330-LMNAgRNA1 plasmid used in this study was modified from the original plasmid to remove the 3XFlag tag from the Cas9 endonuclease previously described (Pauty et al., 2017). U2OS cells were seeded and transfected with indicated siRNA using Lipofectamine RNAiMAX (Invitrogen). Twenty-four hours post-transfection, 1.5 million cells were transfected on the 4D-Nucleofector X-unit (program DG-130), using complete nucleofector solution (SE. Cell Line 4D-Nucleofector X Kit, Lonza) containing 1 μg of pCR2.1-CloverLMNAdonor, 1 μg pX330-LMNAgRNA1, 0.1 μg of piRFP670-N1 (used as transfection control) and 200pmol of siRNA and, immediately resuspended in culture media and transferred to a 10 cm dish. After 48 hr, cells were trypsinized and 0.25 million cells plated into glass coverslips and the rest analyzed on BD Accuri C6 Plus Flow Cytometer. Cloverexpression was assayed by fluorescence microscopy the next day that is 72 hr post-nucleofection. Data represent the mean percentages (±SD) of Clover-positive cells (structured nuclear GFP signal) over the iRFP670-positive population from independent experiments performed in triplicates (n > 800 cells per condition).

NHEJ assay

The U2OS cell line with I-Sce1 reporter GFP-RFP cassette to measure NHEJ has been described (Jacquet et al., 2016). The cells were transfected with 200 nmol of the indicated siRNA using Lipofectamine RNAimax (Invitrogen) for 36 hr and infected with I-Sce1 adenovirus for 1 hr. Cells are harvested 48 hr after DSB induction and analyzed by FACS for GFP and RFP expression on a BD Accuri C6 Plus Flow Cytometer. Control cells produce 10% of RFP positive/GFP negative cells in the NHEJ reporter. The data presented are from biological triplicate experiments.

Cell cycle analysis

For cell cycle studies, DIvA cells were harvested by trypsinization, fixed with cold 70% ethanol, treated with ribonuclease A and propidium iodide. FACS analysis was performed using a BD Accuri C6 Plus Flow Cytometer.

ChIP

ChIP assays were carried out according to the protocol described in Aymard et al., 2014 and Iacovoni et al., 2010. The amount of chromatin and antibodies used are detailed in Table S2. For quantitative PCR analysis (Figure S2), both input and IP samples were analyzed using the primers FW GATTGGCTATGGGTGTGGAC and REV CATCCTTGCAAACCAGTCCT (HR DSB1). For Figure S4, the following primers were also used: FW CCGCCAGAAAGTTTCCTAGA and REV CTCACCCTTGCAGCACTTG (HR-DSB2), FW TGCCGGTCTCCTAGAAGTTG and REV GCGCTTGATTTCCCTGAGT (NHEJ-DSB), FW AGCCGGGCTCTTGCCAAT and REV AGTTAGCGCCCAAAGGACCA (*ACTB*), FW GCTGAGACGAACGCTTCACT and REV CCTTCGAACACTGACCCACT (*TAF12*). ChIP efficiencies were calculated as the percent of input DNA immunoprecipitated. Prior to next-generation sequencing library preparation, samples from multiple ChIP experiments were pooled and sonicated for 5 cycles (30 s on, 30 s off, high setting) with a Bioruptor (Diagenode) then concentrated with a vacuum concentrator (Eppendorf). Sequencing libraries were prepared by using 10 ng of purified DNA (averaged size 250-300 bp), and subjected to high throughput sequencing.

BLESS

BLESS was performed as described in Crosetto et al., 2013. Briefly, cells were fixed with 2% formaldehyde to stabilize chromatin and prevent artificial DSBs, plasma membranes were lysed, and intact nuclei were recovered by centrifugation. Nuclei were deproteinated using Proteinase K. DSBs were then blunted and 5′phosphorylated using the Quick Blunting Kit (NEB) and ligated to a biotinylated linker (proximal) using T4 ligase (NEB). Total DNA was extracted by precipitation with isopropanol and fragmented by sonication using a Covaris S220 to create ~400 bp fragments. Labeled fragments were captured by streptavidin beads (Invitrogen) and once again blunted and phosphorylated using the Quick Blunting Kit (NEB), and ligated to a second linker (distal). The resulting circular DNA was linearized by I-SceI (NEB) digestion and amplified by PCR. Sequencing libraries were prepared using TruSeq Nano DNA LT Library Preparation Kit (Illumina). Quality and quantity of libraries were assessed on 2100 Bioanalyzer (Agilent) using High Sensitivity DNA Kit (Agilent), and on Qubit 2.0 using Qubit dsDNA HS Assay Kit (ThermoFisher).

QUANTIFICATION AND STATISTICAL ANALYSIS

ChIP-seq data processing

H3K4me2, H4K20me1, H3K36me3, H3K79me2, H2Bub, H3K56ac, H4K16ac, H3K9me2, H4S1P, H3K36me2, and γH2AX samples were sequenced using Illumina HiSeq 2500 (single-end, 50 bp reads) at BGI (Beijing Genomics Institute, Hong Kong). FK2 samples were sequenced using Illumina HiSeq 2500 (single-end, 50 bp reads) at GATC biotech (Konstanz, Germany). XRCC4 at 1, 4, and 24 hr post 4OHT treatment, DNA ligase IV, H2BK120ac, H2AZ, H1, MacroH2A, H3K9me3, H4K20me2, 53BP1, and γH2AX G1 and G2 samples were sequenced using Illumina NextSeq 500 (single-end, 75 bp reads) at EMBL Genomics core facilities (Heidelberg, Germany). H4K12ac, H2AZac, and H3 were sequenced using Illumina HiSeq 2500 (single-end, 50 bp reads) at EMBL Genomics

core facilities (Heidelberg, Germany). Previously published data include RAD51 and XRCC4 ChIP-seq in OHT-treated DIvA cells (Aymard et al., 2014), RNA Polymerase II (S2P) in untreated DIvA cells (Cohen et al., 2018) and MethylCap-Seq data in U2OS cells (Deplus et al., 2014)

The quality of each raw sequencing file (fastq) was verified with FastQC (https://www.bioinformatics.babraham.ac.uk/projects/fastqc/). All files were aligned to the reference human genome (hg19) and processed using a classical ChIP-seq pipeline: bwa (http://bio-bwa.sourceforge.net/) for mapping and samtools (http://www.htslib.org/) for duplicate removal (rmdup), sorting (sort) and indexing (index). Coverage for each aligned ChIP-seq dataset (.bam) were computed with the rtracklayer R package and normalized using total read count for each sample. Coverage data were exported as bigwig (file format) for further processing

BLESS data processing

Samples were sequenced with HiSeq 2500 (Illumina) according to our custom protocol for low-diversity samples (Mitra et al., 2015), generating 61-bp paired-end reads containing BLESS barcodes (distal and proximal). Both barcodes were removed using cutadapt, and BLESS data were aligned on hg19 using bwa in paired-end mode (bwa aln and bwa sampe). In order to prevent reads that represent bona fide DSB signal, but start exactly at cleaved AsiSI sites from being marked as duplicates and improperly discarded, fragments were reconstituted from paired reads using Rsamtools and GenomicAlignments (R packages). Fragments with lengths > 500 bp were dropped as aberrant and the remaining fragments were de-duplicated (fragments with exact same start and end position on genome were considered as duplicates and only kept once). To determine which sites among the 1211 AsiSI sites were indeed cleaved, we used the total count for BLESS signal in a 1 kb windows around all AsiSI sites positions on the genome. Outliers (values > third quartile + 1.5 x interquartile range) were considered to be cleaved. Of the 174 sites defined as outliers, we only focused on 80 DSBs (showing the highest BLESS signal) that were significantly induced (Figures 1B–1D, Table S1).

Descriptive statistics using box-plot representation

Each box-plot representation was generated with R-base. The center line represents the median, box ends represent respectively the first and third quartiles, and whiskers represent the minimum and maximum values without outliers. Outliers were defined as first quartile − (1.5 × interquartile range) and above third quartile + (1.5 × interquartile range).

Statistical analysis and representations

Statistical hypothesis testing was performed using nonparametric unpaired Mann-Whitney-Wilcoxon (wilcoxon.test() function in R) to tests distribution differences between two populations. For damaged versus undamaged chromatin features comparisons, boxplots represent the ChIP-seq enrichment ratio between 4OHT treated and untreated DIvA cells (expressed as a log2 ratio) for DSBs or control regions. Significant differences were determined using two-sample Wilcoxon tests. Significant increases are colored in red or orange (for p value < 0.01 or p value < 0.05, respectively), significant decreases in dark or light blue (for p value < 0.01 or p value < 0.05, respectively) and non-significant differences (ns, p value > 0.05) in gray.

In the circle plot representation (Figures 4F and S5E), each circle radius represents the p value of a nonparametric two-sided Mann-Whitney-Wilcoxon test comparing ChIP-seq counts in a given window size. In Figures 4E and S5D, total ChIP-seq count for each dataset (untreated condition) was compared for HR and NHEJ DSBs. Individual modifications were determined as enriched in HR or NHEJ using one-sided Mann-Whitney-Wilcoxon tests (if p value < 0.05). In Figure S5E, total ChIP-seq count for each dataset was compared in treated and untreated conditions, separately for HR or NHEJ DSBs. Individual modifications were determined as increased or decreased following DSB induction using one-sided Mann-Whitney-Wilcoxon tests (p value < 0.05). In Figure S6B, Spearman correlation coefficients were determined using the cor() function in R and the correlation matrix was generated using the corrplot package. For Figure S4B, to check if DSBs repaired by HR or NHEJ exhibit a non-random distribution among the main 3D genomic compartments (A1, A2, B1, B2, B3, B4 and NA (non-assigned)) (Rao et al., 2014)), we compared the distribution of all genomic loops among these compartments with the distributions for loops containing either HR-repaired or NHEJ-repaired DSBs (no loop contained both HR and NHEJ sites). The p values were calculated using hypergeometric probability distribution, with the null hypothesis that HR and NHEJ loops are distributed among the 3D compartments in the same manner as all genomic loops. Conservative Bonferroni correction for multiple hypothesis testing was applied to correct p values to reflect testing all 3D compartments for both possible enrichments and depletions.

Averaged ChIP-seq profiles

Averaged ChIP-seq profiles were generated using the R package ggplot2. The x axis represents genomic position relative to DSB and the y axis represents the mean coverage at each bp, except for larger windows (1 Mb scale) where data were smoothed using a 50 kb span. Log2 ratio was computed using the bamCompare tool from deepTools (http://deeptools.readthedocs.io) with two bam files (before and after damage) as inputs. Positive and negative values for log2 ratio are respectively represented in red and blue. For γH2AX, 53BP1, RAD51, and XRCC4, ChIP-seq data are only available for damaged cells. In this case, the y axis only represents the mean ChIP-seq coverage in the damaged condition.

Generation of random positions

In order to generate a control set of non-DSB regions, we first computed a thousand random positions on the entire genome (excluding chromosome Y) using R. These random sites were filtered for being at least 1 Mb away from the top 150 cleaved positions with IRanges::findOverlaps() function. We also excluded regions with a null ChIP-seq count (in any of our datasets) in a 1 kb window in order to avoid regions that are systematically underrepresented in ChIP-seq experiments. Finally, 80 sites were randomly picked from the remaining list.

Determination of HR-prone and NHEJ-prone DSBs

We computed a ChIP-seq coverage ratio between RAD51 (4 kb window) and XRCC4 (1 kb window) for each of the 80 induced DSBs. Sites with the highest ratio were designated as HR-prone and sites with the lowest ratio as NHEJ-prone (30 DSBs in each category). For the analysis performed Figure S5D, HR-prone and NHEJ–prone DSBs were obtained by computing the ratio between RAD51 (4 kb window) and Ligase IV (1 kb window) for each of the 80 induced DSBs and selecting sites with highest and lowest ratios (30 DSBs in each category).

DATA AND SOFTWARE AVAILABILITY

The accession number for the high-throughput sequencing data reported in this paper is ArrayExpress: E-MTAB-5817.

Source code for generating boxplots, heatmaps, and average profiles is available on the GitHub repository page: https://github.com/LegubeDNAREPAIR/HistoneMapping.

Molecular Cell, Volume *72*

Supplemental Information

Comprehensive Mapping of Histone Modifications at DNA Double-Strand Breaks Deciphers Repair Pathway Chromatin Signatures

Thomas Clouaire, Vincent Rocher, Anahita Lashgari, Coline Arnould, Marion Aguirrebengoa, Anna Biernacka, Magdalena Skrzypczak, François Aymard, Bernard Fongang, Norbert Dojer, Jason S. Iacovoni, Maga Rowicka, Krzysztof Ginalski, Jacques Côté, and Gaëlle Legube

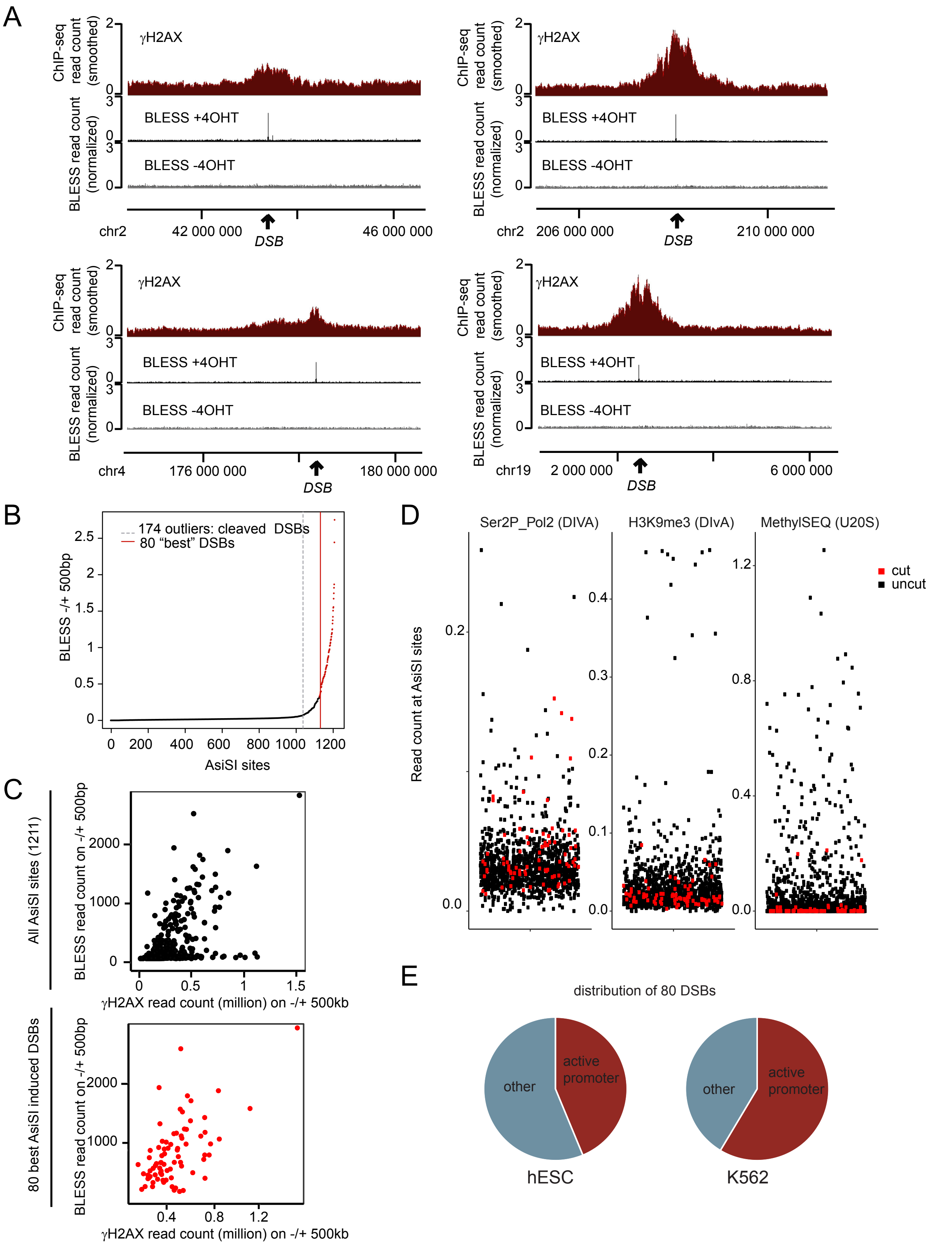
A
ChIP-seq read count (smoothed)
γH2AX
BLESS read count (normalized)
BLESS +4OHT
BLESS -4OHT
chr2
42 000 000
46 000 000
DSB
206 000 000
210 000 000
chr4
176 000 000
180 000 000
chr19
2 000 000
6 000 000
B
174 outliers: cleaved DSBs
80 "best" DSBs
BLESS -/+ 500bp
AsiSI sites
C
All AsiSI sites (1211)
BLESS read count on -/+ 500bp
γH2AX read count (million) on -/+ 500kb
80 best AsiSI induced DSBs
D
Ser2P_Pol2 (DIVA)
H3K9me3 (DIvA)
MethylSEQ (U20S)
Read count at AsiSI sites
cut
uncut
E
distribution of 80 DSBs
other
active promoter
hESC
K562

Figure S1. related to Figure 1: AsiSI-induced DSB mapping by BLESS

(**A**) Genome browser screenshots representing γH2AX ChIP-seq (4OHT treated cells) and BLESS (4OHT untreated or treated cells) signal at DSBs located on chromosome 2, 4 and 19 as indicated.
(**B**) Dotplot representing BLESS read count in a 1 kb window for the 1211 predicted AsiSI sites in the human genome. Sites are sorted by increasing signal.
(**C**) Scatterplot representing read count (from 4OHT treated cells) for γH2AX ChIP-seq (in a 1 Mb window) and BLESS (in a 1 kb window) for the 1211 predicted AsiSI sites in the human genome (upper panel) and the 80 most cleaved AsiSI sites (lower panel).
(**D**) Dotplot representing MethylCap-seq read count obtained in U20S (Deplus et al., 2014), on a 200bp window (right panel), H3K9me3 ChIP-seq read count on a 10kb window (middle panel) for and RNA PolII-S2P ChIP-seq (Cohen et al., 2018) read count in a 10 kb window (left panel) for each of the 1211 predicted AsiSI sites in the human genome. Cut sites are indicated in red.
(**E**) The 80 DSBs were compared with the chromatin state segmentation track from hESC and K562 cells (Broad ChromHMM, http://rohsdb.cmb.usc.edu/GBshape/cgi-bin/hgTrackUi?db=hg19&g=wgEncodeBroadHmm). The proportion of DSBs lying within active promoters (dark red) or other loci (grey) are shown.

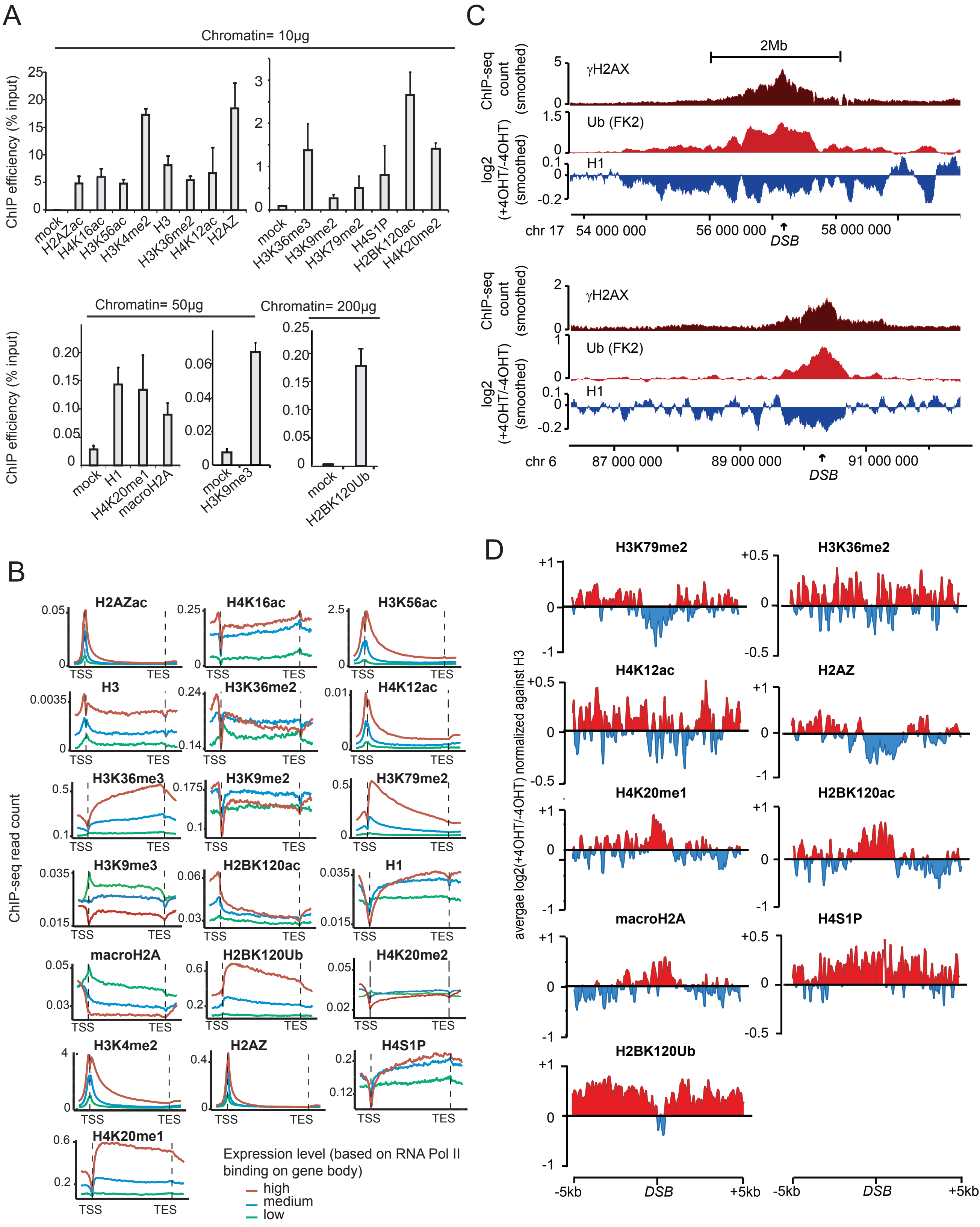
A
Chromatin= 10µg
ChIP efficiency (% input)
mock
H2AZac
H4K16ac
H3K56ac
H3K4me2
H3
H3K36me2
H4K12ac
H2AZ
H3K36me3
H3K9me2
H3K79me2
H4S1P
H2BK120ac
H4K20me2
Chromatin= 50µg
Chromatin= 200µg
H1
H4K20me1
macroH2A
H3K9me3
H2BK120Ub
B
ChIP-seq read count
TSS
TES
H2AZac
H4K16ac
H3K56ac
H3
H3K36me2
H4K12ac
H3K36me3
H3K9me2
H3K79me2
H3K9me3
H2BK120ac
H1
macroH2A
H2BK120Ub
H4K20me2
H3K4me2
H2AZ
H4S1P
H4K20me1
Expression level (based on RNA Pol II binding on gene body)
high
medium
low
C
2Mb
ChIP-seq count (smoothed)
log2 (+4OHT/-4OHT) (smoothed)
γH2AX
Ub (FK2)
H1
chr 17
54 000 000
56 000 000
58 000 000
DSB
chr 6
87 000 000
89 000 000
91 000 000
DSB
D
averge log2(+4OHT/-4OHT) normalized against H3
H3K79me2
H3K36me2
H4K12ac
H2AZ
H4K20me1
H2BK120ac
macroH2A
H4S1P
H2BK120Ub
-5kb
DSB
+5kb

Figure S2, related to Figure 2 and 3: ChIP-seq validation and histone modification changes following AsiSI induction in DIvA cells

(**A**) ChIP were performed in untreated DIvA cells with all indicated antibodies and qPCR was performed to assess enrichment at a specific genomic location (chr1: 89458701, hg19), an AsiSI cut site. Average and SEM of at least 3 independent experiments are shown.
(**B**) Average profile for each histone modification obtained in untreated samples over human genes sorted by expression level (high in red, medium in blue, low in red). This recapitulate previous findings (Barski et al., 2007; Chen et al., 2012; Gamble et al., 2010; Gatta et al., 2011; Jung et al., 2012; Krishnakumar et al., 2008; Kuo et al., 2011; Lo et al., 2011; Millan-Arino et al., 2014; Nelson et al., 2016; Tolstorukov et al., 2012; Vakoc et al., 2006; Valdes-Mora et al., 2012; Wang et al., 2013; Wang et al., 2008). See also Table S2 for additional references.
(**C**) Genome Browser screenshots representing ChIP-seq signals for γH2AX, ubiquitin, and H1 at 2 DSBs located on chromosome 17 and 6 respectively. Data are expressed as read count (from 4OHT treated samples) for γH2AXand as a log2 ratio between 4OHT treated and untreated DIvA cells for ubiquitin (FK2) and H1, smoothed using a 100kb span.
(**D**) Average profile on a 10 kb window of the H3 normalized enrichment between 4OHT treated and untreated DIvA cells for the nine histone modifications that exhibited significant changes over 80 DSBs (Figure 3). Values are expressed as log2 ratios. Positive and negative values for log2 ratio are respectively represented in red and blue.

Clouaire, Figure S3

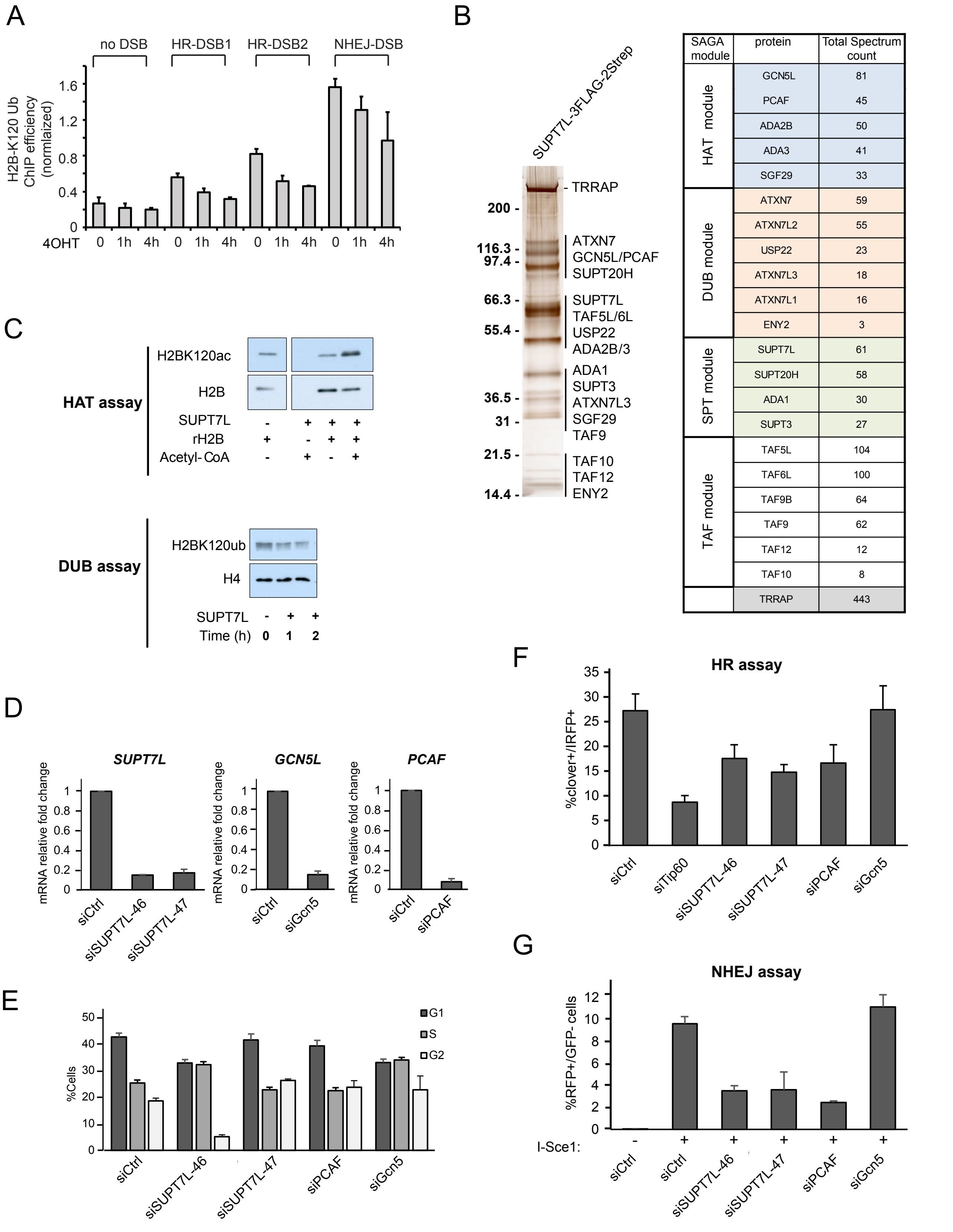

SAGA module	protein	Total Spectrum count
HAT module	GCN5L	81
	PCAF	45
	ADA2B	50
	ADA3	41
	SGF29	33
DUB module	ATXN7	59
	ATXN7L2	55
	USP22	23
	ATXN7L3	18
	ATXN7L1	16
	ENY2	3
SPT module	SUPT7L	61
	SUPT20H	58
	ADA1	30
	SUPT3	27
TAF module	TAF5L	104
	TAF6L	100
	TAF9B	64
	TAF9	62
	TAF12	12
	TAF10	8
	TRRAP	443

Figure S3, related to Figure 3: hSAGA can catalyze *in vitro* H2BK120 acetylation and deubiquitination, and contributes to DSB repair

(**A**) ChIP against H2BK120 Ubiquitination was performed in DIvA cells, either left untreated or treated with 4OHT for 1h or 4h as indicated. Enrichment was measured at a control locus for normalization (*TAF12*) and at an uncut genomic location (*ACTB*) as well as 3 AsiSI-induced DSBs (average ± SEM, n=2).

(**B**) Purification of native human SAGA complex from K562 cells. Cells expressing SUPT7L-3Flag-2Strep from the AAVS1 safe harbor were engineered and used for tandem affinity purification from nuclear extracts. The purified fraction was analyzed on gel (silver stained) and by tandem mass spectrometry to confirm purity and the copurification of all known SAGA subunits (and paralogs). Total spectral counts obtained for each subunit are presented in the table and are grouped by functional modules within the complex.

(**C**) (Top panel) In vitro histone acetyltransferase assay with purified SAGA complex with histone H2B. H2BK120 acetylation is measured by western blot analysis using total H2B signal as control. (Bottom panel) In vitro deubiquitinase assay with purified SAGA complex and human native nucleosomes. H2BK120 deubiquitination is measured by western blot using total H4 signal as a control.

(**D**) RT-qPCR showing the mRNA levels of SUPT7L, GCN5L and PCAF in cells before and after knockdown using indicated siRNAs. The mean of 3 independent experiments ± SD is shown.

(**E**) Cell cycle distributions of U2OS cells following transfection with the indicated siRNA for 72h. The mean of 2 independent experiments ± SEM is shown.

(**F**) Effect of SUPT7L, PCAF and GCN5L knockdown on DSB repair by HR using a Cas9/mClover-LMNA1 homologous recombination assay. GFP+(mClover)/iRFP+ cells were measured by FACS and the structured nuclear GFP signal linked to LMNA1 was confirmed by microscopy. Results represent the percentage of GFP+/iRFP+ cells from 3 independent experiments (average ± SD).

(**G**) Effect of SUPT7L, PCAF and GCN5L knockdown on DSB repair by NHEJ. Measurement of I-Sce1 DSB repair by non-homologous end joining in U2OS cells using an integrated PC222/GFP-RFP reporter. Cells were transfected with the indicated siRNAs for 36h, infected with I-Sce1 adenovirus to induce DSB and then assessed 48h later by FACS analysis for RFP and GFP expression. Results represent the percentage of cells that are RFP positive but GFP negative, from 3 independent experiments (average ± SD).

Clouaire, Figure S4

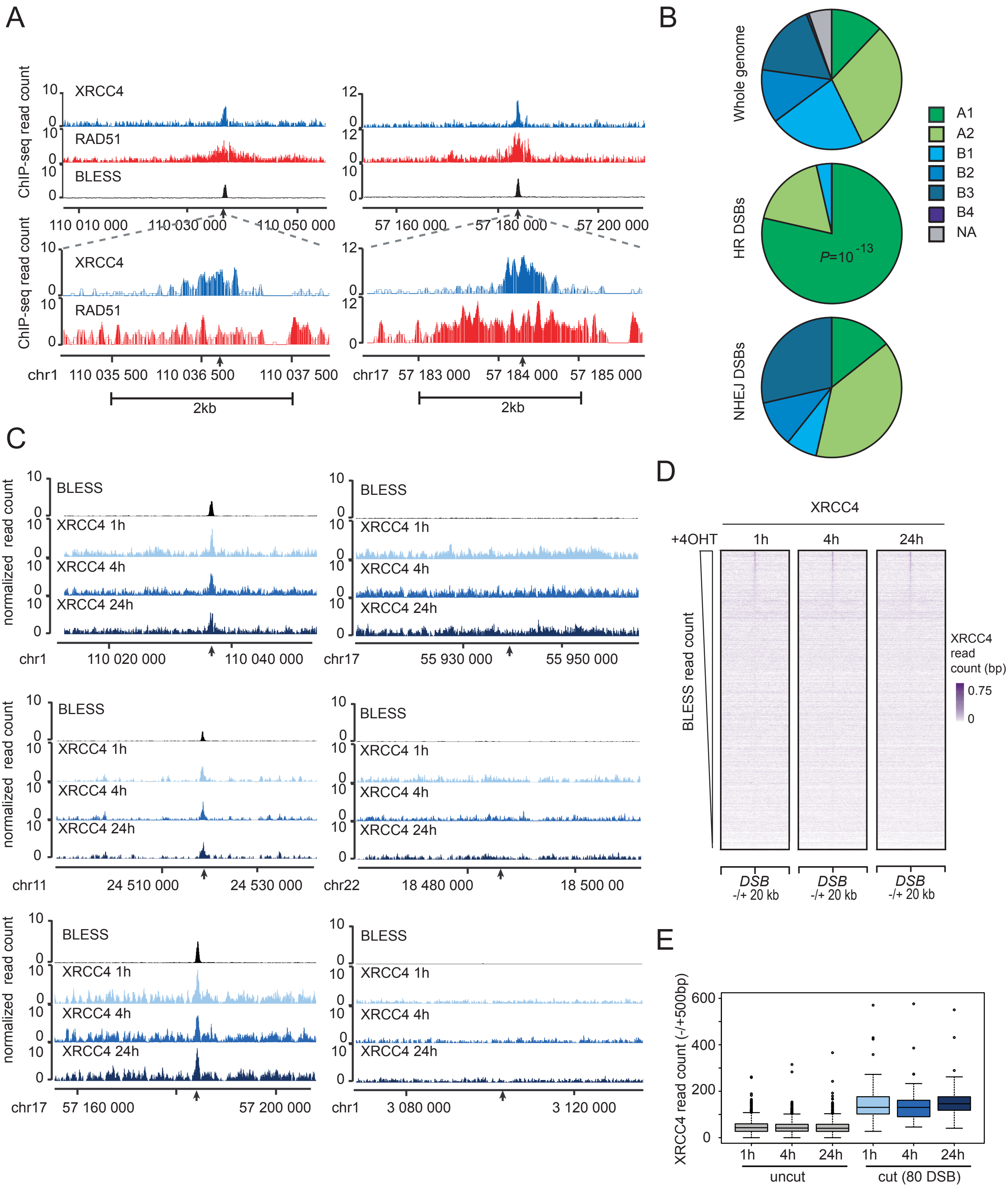

Figure S4, related to Figure 4: Identification HR and NHEJ-prone DSBs

(**A**) Genome Browser screenshots representing read counts in 4OHT treated cells for XRCC4, RAD51 and BLESS signal at two DSBs located on chromosome 1 (left) and 17 (right). Close ups are presented on bottom panels.
(**B**) Pie chart representing the distribution of loops within the different nuclear compartments (A1, A2, B1, B2, B3, B4 and NA (Rao et al., 2014)). Left panel shows distribution for all loops identified across the genome (Rao et al., 2014) , middle panel for loops containing HR-prone DSBs, and right panel for loops containing NHEJ-prone DSBs. HR-prone DSBs are very significantly enriched ($P=10^{-13}$, hypergeometric test) in the nuclear A1 compartment.
(**C**) Genome Browser screenshots representing XRCC4 ChIP-seq read counts after 1h, 4h or 24h following 4OHT addition at three DSBs exhibiting BLESS signal (cut, left panels) or no BLESS signal (uncut, right panel)
(**D**) Heatmaps representing the XRCC4 signals on a 40kb window centered around all AsiSI sites, ordered based on the BLESS level, at 1h, 4h and 24h following 4OHT treatment.
(**E**) Box plot showing XRCC4 signal on a 1kb window surrounding uncut or cut DSBs, following 1h, 4h and 24h 4OHT treatment as indicated.

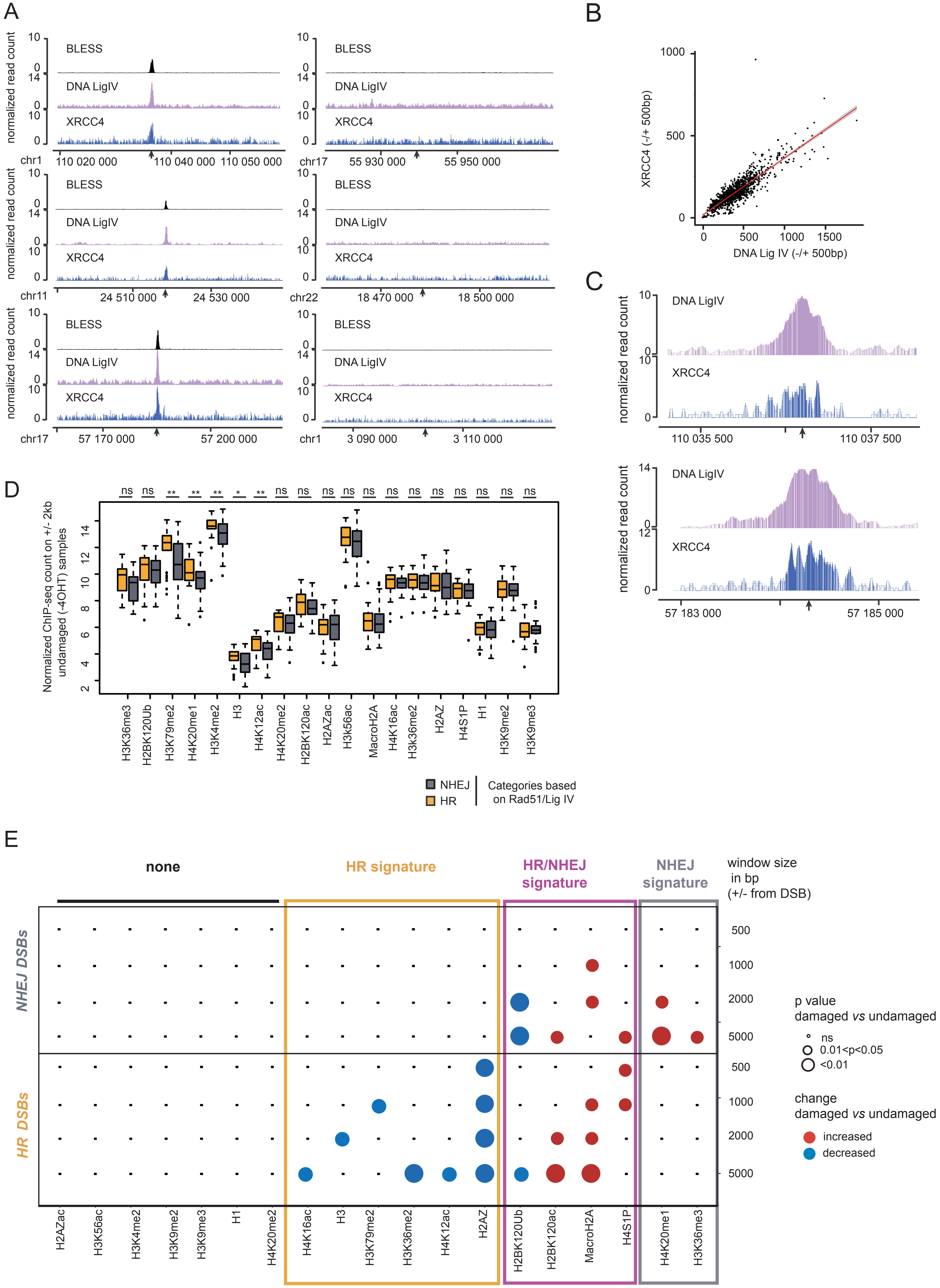
A
BLESS
DNA LigIV
XRCC4
normalized read count
chr1
110 020 000
110 040 000
110 050 000
chr17
55 930 000
55 950 000
chr11
24 510 000
24 530 000
chr22
18 470 000
18 500 000
57 170 000
57 200 000
3 090 000
3 110 000
B
XRCC4 (-/+ 500bp)
DNA Lig IV (-/+ 500bp)
C
110 035 500
110 037 500
57 183 000
57 185 000
D
Normalized ChIP-seq count on +/- 2kb undamaged (-4OHT) samples
H3K36me3
H2BK120Ub
H3K79me2
H4K20me1
H3K4me2
H3
H4K12ac
H4K20me2
H2BK120ac
H2AZac
H3K56ac
MacroH2A
H4K16ac
H3K36me2
H2AZ
H4S1P
H1
H3K9me2
H3K9me3
NHEJ
HR
Categories based on Rad51/Lig IV
E
none
HR signature
HR/NHEJ signature
NHEJ signature
window size in bp (+/- from DSB)
NHEJ DSBs
HR DSBs
p value damaged vs undamaged
ns
0.01<p<0.05
<0.01
change damaged vs undamaged
increased
decreased

Figure S5, related to Figure 4 and 5: Use of DNA Lig IV to confirm HR and NHEJ-prone DSBs and characterization of HR and NHEJ histone signature

(**A**) Genome Browser screenshots representing XRCC4 and DNA ligase IV ChIP-seq read counts after 4h following 4OHT addition at three DSBs exhibiting BLESS signal (cut, left panels) or no BLESS signal (uncut, right panel). Regions are the same as in Figure S4C.
(**B**) Scatterplot showing the level of DNA ligase IV (x axis) and XRCC4 (y axis) on a 1kb window around each annotated AsiSI sites
(**C**) Genome Browser screenshots (close up) representing read counts in 4OHT treated cells for XRCC4 and DNA Ligase IV at two DSBs induced by AsiSI
(**D**) Boxplot representing the ChIP-seq read count in a 4 kb window for each histone modification in untreated cells for 30 HR (yellow) and 30 NHEJ (grey) DSBs determined using Rad51/DNA ligase IV ratio instead of Rad51/XRCC4. P-values were calculated using two-sample Wilcoxon test. *$P < 0.05$, **$P < 0.01$; $P > 0.05$ is not significant (ns).
(**E**) Circle plot analysis showing significant changes observed between 4OHT treated and untreated DIvA cells at HR-prone (bottom) and NHEJ prone (top) DSBs, using increasing window size. Radius size represents P-values (from two-sample Wilcoxon test) when comparing ChIP-seq signal for treated and untreated samples. Significant increases (+4OHT>-4OHT) are colored in red, while significant decreases (+4OHT<-4OHT) are colored in blue. Histone modifications that undergo significant changes only at HR-DSBs are squared in yellow, those that change at both HR and NHEJ in purple and those specific for NHEJ in grey.

Clouaire, Figure S6

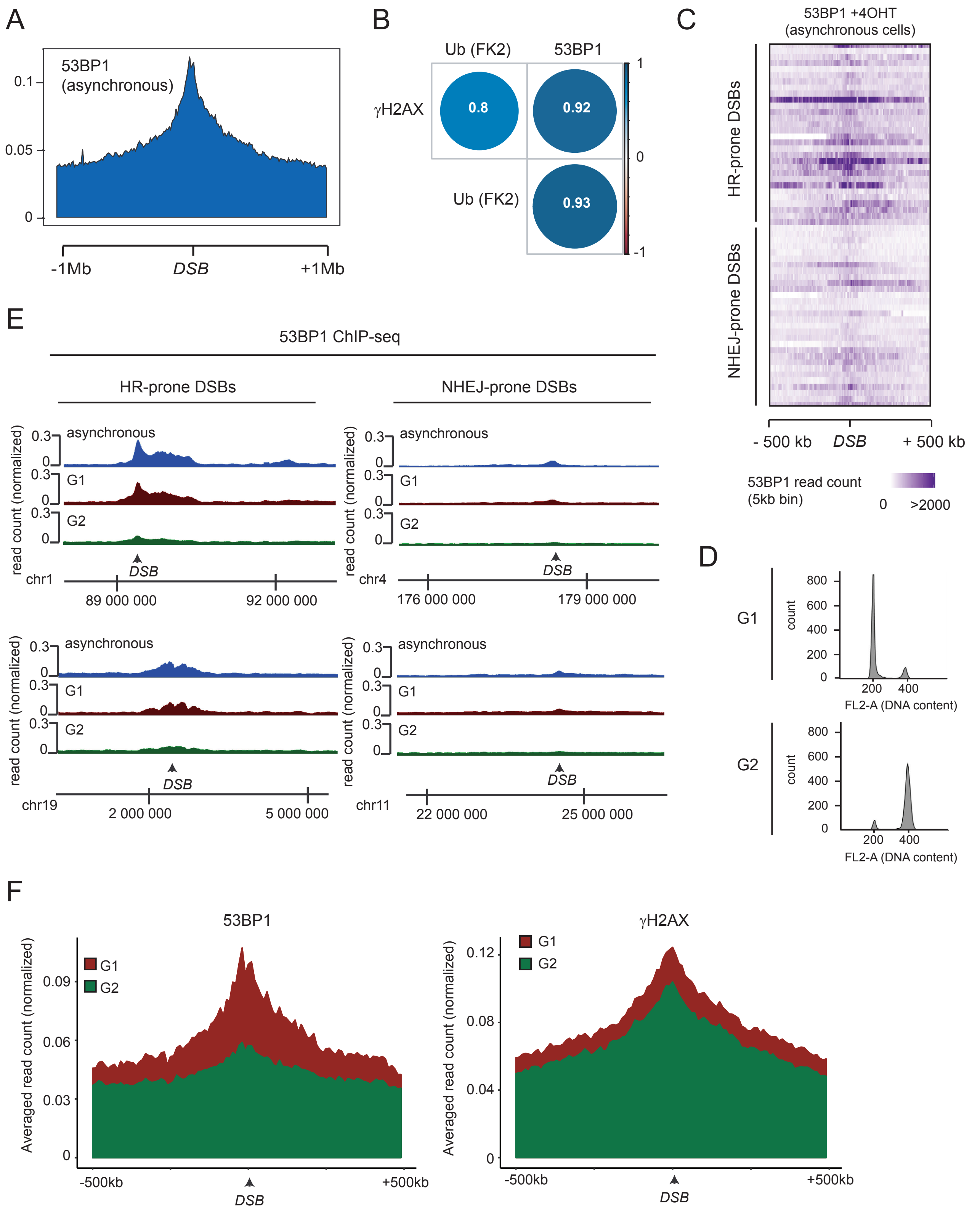

Figure S6, related to Figure 7: 53BP1 distribution analyzed by ChIP-seq

(**A**) Average profile for 53BP1 ChIP-seq (read count from 4OHT treated cells) for 80 DSBs in a 2 Mb window.
(**B**) Spearman correlation matrix of ChIP-seq read count (from 4OHT treated cells) for γH2AX, ubiquitin (FK2) and 53BP1 for 80 DSBs in a 1 Mb window.
(**C**) Heatmap representing the 53BP1 signal on a 1Mb window centered around 30 HR (top part) and 30 NHEJ sites (bottom part).
(**D**) FACS profiles indicating the cell cycle distribution for G1- and G2- 53BP1 and γH2AX ChIP-seq.
(**E**) Genome Browser screenshots representing 53BP1 ChIP-seq signals (from 4OHT treated samples) in asynchronous, G1 or G2 synchronized DIvA cells for 2HR and 2 NHEJ-prone DSBs.
(**F**) Average profiles for 53BP1 (left) and γH2AX (right) ChIP-seq in G1 (red) and G2 (green) synchronized cells (read count from 4OHT treated cells) for 80 DSBs in a 1 Mb window.

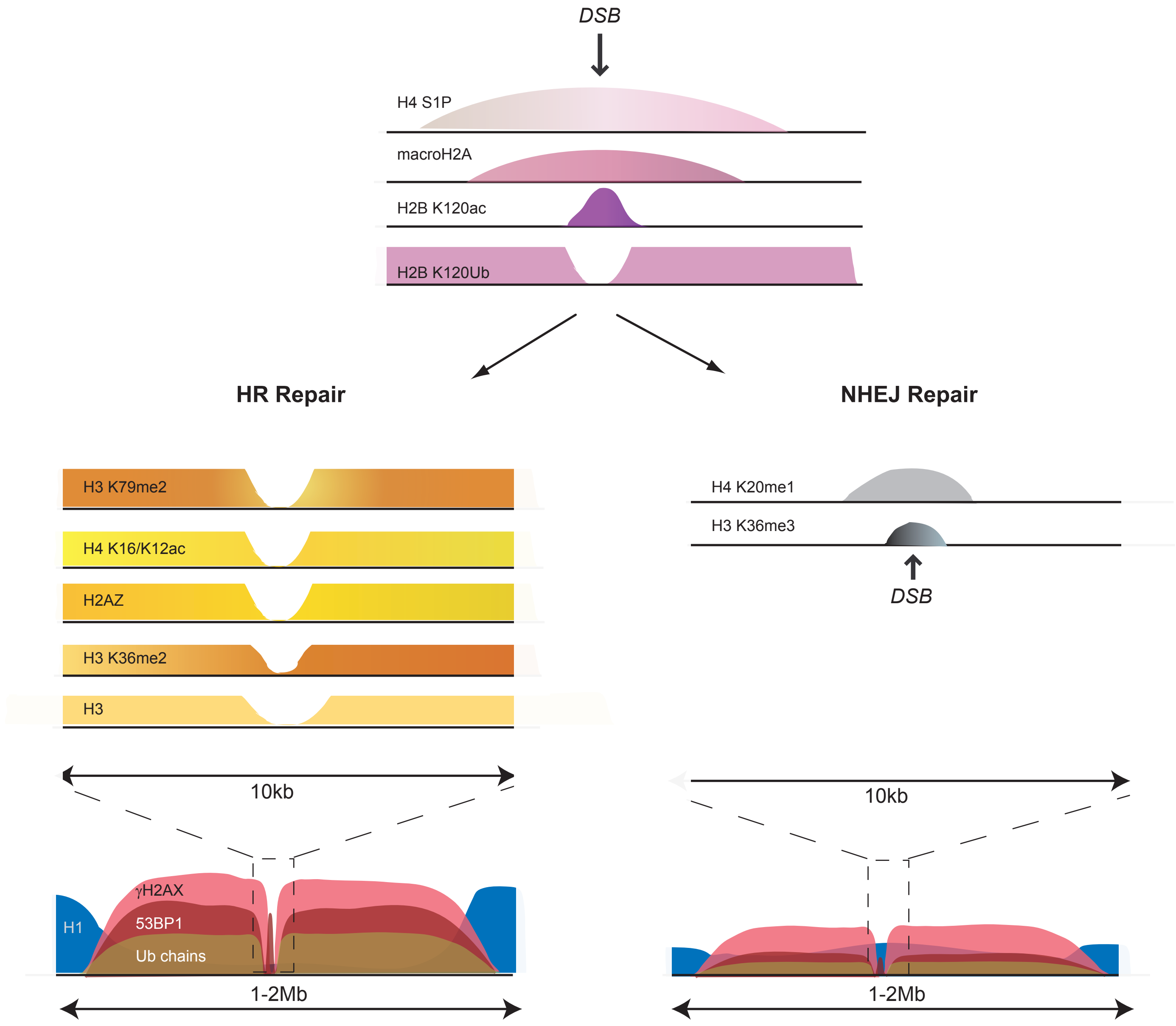
DSB
H4 S1P
macroH2A
H2B K120ac
H2B K120Ub
HR Repair
NHEJ Repair
H3 K79me2
H4 K16/K12ac
H2AZ
H3 K36me2
H3
10kb
γH2AX
H1
53BP1
Ub chains
1-2Mb
H4 K20me1
H3 K36me3
DSB
10kb
1-2Mb

Figure S7, related to Figure 2-7: **Summary for DSB-induced chromatin changes**

Following DSB induction, macroH2A is deposited, H4 is phosphorylated on Serine 1 and H2B undergoes a switch from ubiquitination to acetylation on lysine 120. At DSBs repaired by NHEJ, this is accompanied by an increase of H4 monomethylation on lysine 20 and H3 trimethylation on lysine 36. At DSB repaired by HR, which mainly reside in transcriptionally active chromatin, these chromatin changes are also associated with the demethylation of H3K79me2, deacetylation of H4 and H2AZ removal, all previously known to crosstalk with H2BK120 monoubiquitination. HR-prone DSBs also experience an acute, large-scale chromatin signaling with accumulation of γH2AX and ubiquitin conjugates, depletion of histone H1, and 53BP1 binding. While γH2AX signaling occurs at all cell cycle phase, 53BP1 mainly accumulates at HR-prone DSBs during G1. Such modifications on the megabase scale likely alters chromatin fiber properties to be translated into changes in chromatin mobility within the nucleus. This could potentially favor homology search and/or clustering, features of HR-prone DSBs.

chromosome	start	end
chr1	9649446	9649452
chr1	40974644	40974650
chr1	89458597	89458603
chr1	110036700	110036706
chr1	204380453	204380459
chr1	224032648	224032654
chr2	43358339	43358345
chr2	55509101	55509107
chr2	68384749	68384755
chr2	74734762	74734768
chr2	85822594	85822600
chr2	120124566	120124572
chr2	208030728	208030734
chr3	52232163	52232169
chr3	98618165	98618171
chr3	99536965	99536971
chr4	83934287	83934293
chr4	178363576	178363582
chr5	68462851	68462857
chr5	79784140	79784146
chr5	142785050	142785056
chr6	27145367	27145373
chr6	31105428	31105434
chr6	37321812	37321818
chr6	49917583	49917589
chr6	67704021	67704027
chr6	90348187	90348193
chr6	135819348	135819354
chr6	144607569	144607575
chr6	149888106	149888112
chr7	75807507	75807513
chr7	92861491	92861497
chr7	99679508	99679514
chr8	66546348	66546354
chr8	116680632	116680638
chr8	124781210	124781216
chr9	29212800	29212806
chr9	36258514	36258520
chr9	127532106	127532112
chr9	130693171	130693177
chr9	130889408	130889414

chromosome	start	end
chr10	3110978	3110984
chr10	94051015	94051021
chr11	24518476	24518482
chr11	75525761	75525767
chr11	85375655	85375661
chr12	13154718	13154724
chr12	22093989	22093995
chr12	121975058	121975064
chr12	130091881	130091887
chr13	105238552	105238558
chr13	114894659	114894665
chr14	54955826	54955832
chr17	5390221	5390227
chr17	38137473	38137479
chr17	57184297	57184303
chr17	61850856	61850862
chr17	80250841	80250847
chr18	7566713	7566719
chr18	19320805	19320811
chr19	2456094	2456100
chr19	30019488	30019494
chr19	41903743	41903749
chr19	42497856	42497862
chr19	45932080	45932086
chr19	46768784	46768790
chr20	1207616	1207622
chr20	20032925	20032931
chr20	30946313	30946319
chr20	32032087	32032093
chr20	37360269	37360275
chr20	42087118	42087124
chr21	33245519	33245525
chr21	46221790	46221796
chr22	20850308	20850314
chr22	38864102	38864108
chrX	1510672	1510678
chrX	45366394	45366400
chrX	53111427	53111433
chrX	72783103	72783109

Table S1, related to Figure 1: Genomic coordinates (hg19) of the top 80 AsiSI induced DSBs identified by BLESS

	Chromatin features	Antibody used in this study	Amount of chromatin for ChIP	Status at DSBs	Proposed function in DSB repair	References	Previous genome-wide profiling
**Histone**	H3	Abcam ab1791	10µg	Decreased	Removal proposed to Increase DNA accessibility or be a consequence of resection. Unclear given the ability of nucleosome to also form on ssDNA and given that it also occur at DSB repaired by NHEJ	[1-7]	ChIP-seq in mouse cells [8] Same antibody
	H2AZ	Abcam ab4174	10µg	Transiently deposited and quickly removed	Proposed to be incorporated to promote NHEJ. Prevents unlimited resection and the use of Alt-NHEJ and SSA. Needs to be removed by INO80 and ANP32E to promote Rad51 foci formation and HR. In yeast, promotes the anchoring of persistent DSBs to nuclear periphery	[9-15]	ChIP-seq in human cells [16] Same antibody
	H1	Abcam ab17677	50µg	Decreased	Polyubiquitinated at DSB by RNF8, phosphorylated by DNA-PK and Parylated. All modifications may loosen its interaction with DNA. Removal proposed to stimulates repair by C-NHEJ and HR and retention increases Alt-NHEJ	[17-22]	ChIP-chip in human cells [23, 24] Different antibodies
	macroH2A	Millipore 07-219	50µg	Increased	Promote homologous recombination and BRCA1 recruitment/Establishment of a "heterochromatin like" (nuclease resistant, condensed) state in DSB flanking chromatin	[25-28]	ChIP-chip and ChIP-seq in human cells [29-31] Same antibody
**Modified Histones**	H2AZac	Abcam ab18262	10µg	Not assessed			ChIP-seq in human cells [31] Same antibody
	H3K79me2	Active Motif 39143	10µg	Unchanged	In vitro binds to Tudor domain of Crb2 (53BP1 orthologue) in yeast. Low affinity for 53BP1 in mammals. Promote 53BP1 foci assembly. During transcription crosstalk with H2BUb, proposed to promote relaxation	[32-37]	ChIP seq in mouse cells [8, 38] Same antibody
	H4K20me1	Active Motif 39727	10µg	Conflicting. Found as increased or unchanged	In vitro binds to Tudor domain of 53BP1 or Crb2. 53BP1 Promote 53BP1 foci assembly and NHEJ. Inhibits HR	[32, 39-45]	ChIP-seq in human cells [16] Different antibody
	H3K9me2	Abcam ab1220	10µg	Conflicting. Independently found as increased, decreased and unchanged	Promote homologous recombination/ Establishment of a "heterochromatin like" (nuclease resistant, condensed) state in DSB flanking chromatin.	[25, 46-48]	ChIP-seq in human cells [16] Same antibody
	H3K9me3	Abcam ab8898	50µg	Conflicting. Independently found as increased and unchanged	Proposed to be required for ATM activation at DSB	[46, 49].	ChIP-seq in human cells [16] Same antibody
	H3K4me2	Millipore 07-030	10µg	Conflicting. Independently found as increased and decreased	Proposed to promote relaxation	[50, 51]	ChIP-chip in human cells [52] Same antibody
	H3K36me2	Abcam ab9049	10µg	Increased	Promotes Ku70, NBS1 and MRE11 recruitment at DSB. Proposed to promote NHEJ	[46, 53, 54]	ChIP-seq in mouse cells [55, 56] Same and different antibody
	H3K36me3	Abcam ab9050	10µg	Unchanged	Interacts with LEDGF. Recruits CtIP and promotes HR	[57-59]	ChIP-seq in human cells [16] Same antibody
	H4K12ac	Abcam ab46983	10µg	transient increase	Acetylated by NuA4/Tip60. Proposed to contribute to relaxation	[60-62]	ChIP-seq in mouse cells [63] Same antibody
	H4K16ac	Millipore 07-329	200µg	Found as increased, unchanged and decreased	Required for BRCA1 recruitment and antagonizes 53BP1 binding to methylated H4K20. Proposed to facilitate resection. First decreased and then increased	[62, 64-67] 1144-51.	ChIP seq in mouse cells [68] Same antibody

	H3K56ac	Abcam ab7-307	10µg	Conflicting. Independently found as increased and decreased	Deacteylation contribute to RPA, BRCA1 and 53BP1 recruitment/ Deacetylation proposed to promote NHEJ/ Reacetylation proposed to be required for checkpoint recovery after repair	[2, 65, 69-73]	ChIP-seq in human cells [31, 74] Same antibody
	H4S1P	Novus NB21-2000	10µg	Increase at DSB (in yeast)	promotes NHEJ	[75, 76]	Not reported previously
	H4K20me2	Abcam ab9052	10µg	Described increased at DSB	Favors 53BP1 recruitment at DSB sites	[32, 39, 43, 45, 77, 78]	Not reported previously
	H2BK120Ub	Cell Signalling D11 5546P	200µg	Increased at DSB	Counteracts 53BP1 loading, stimulates range resection, promotes BRCA1, Rad51 loading and HR. Stimulates H3K4 methylation and K79me2. Proposed to promote relaxation	[50, 51, 79-81]	ChIP-seq in human cells [82] Same antibody
	H2BK120ac	Millipore 07-564	10µg	Not assessed	Antagonize H2BK120Ub. Promoted by macroH2A and PARP activity during transcription	[29]	ChIP-seq in human cells [83] Same antibody
	Ubiquitin conjugates	Millipore 04-263	200µg	Increased at DSB	Form foci upon DSB.	[84, 85]	Not reported previously
	γH2AX	Abcam ab81299	200µg	Increased at DSB	Form foci upon DSB.	[86]	ChIP-chip in human cells [87] Same antibody
**Repair proteins**	XRCC4	Abcam ab145	200µg		involved in NHEJ		
	RAD51	Santa Cruz H-92	200µg		involved in HR		
	53BP1	Novus NB100-305	200µg		Counteracts resection and BRCA1. Binds to H4K20 methylated (mainly mono and di). Binds to H2A ubiquitinated on K15. May directly interact with H2AX		
	DNA Lig IV	Genetex GTX55592	200µg		involved in NHEJ		

Table S2, Related to Figure 2, Figure 3 and STAR Methods: Histone modification summary table

Proposed functions and status at DSB (from previous reports) for each histone modifications analyzed in this study. The antibodies, the amount of chromatin used for ChIP-seq and previous reports of genome-wide mapping are also provided

References

1. Adkins, N.L., et al., *Nucleosome-like, Single-stranded DNA (ssDNA)-Histone Octamer Complexes and the Implication for DNA Double Strand Break Repair*. J Biol Chem, 2017. **292**(13): p. 5271-5281.
2. Chen, C.C., et al., *Acetylated lysine 56 on histone H3 drives chromatin assembly after repair and signals for the completion of repair*. Cell, 2008. **134**(2): p. 231-43.
3. Goldstein, M., et al., *Nucleolin mediates nucleosome disruption critical for DNA double-strand break repair*. Proc Natl Acad Sci U S A, 2013. **110**(42): p. 16874-9.
4. Li, X. and J.K. Tyler, *Nucleosome disassembly during human non-homologous end joining followed by concerted HIRA- and CAF-1-dependent reassembly*. Elife, 2016. **5**.
5. Shim, E.Y., et al., *RSC mobilizes nucleosomes to improve accessibility of repair machinery to the damaged chromatin*. Mol Cell Biol, 2007. **27**(5): p. 1602-13.

6. Tsabar, M., et al., *Re-establishment of nucleosome occupancy during double-strand break repair in budding yeast.* DNA Repair (Amst), 2016. **47**: p. 21-29.
7. Tsukuda, T., et al., *Chromatin remodelling at a DNA double-strand break site in Saccharomyces cerevisiae.* Nature, 2005. **438**(7066): p. 379-83.
8. Chronis, C., et al., *Cooperative Binding of Transcription Factors Orchestrates Reprogramming.* Cell, 2017. **168**(3): p. 442-459 e20.
9. Alatwi, H.E. and J.A. Downs, *Removal of H2A.Z by INO80 promotes homologous recombination.* EMBO Rep, 2015. **16**(8): p. 986-94.
10. Gursoy-Yuzugullu, O., M.K. Ayrapetov, and B.D. Price, *Histone chaperone Anp32e removes H2A.Z from DNA double-strand breaks and promotes nucleosome reorganization and DNA repair.* Proc Natl Acad Sci U S A, 2015. **112**(24): p. 7507-12.
11. Horigome, C., et al., *SWR1 and INO80 chromatin remodelers contribute to DNA double-strand break perinuclear anchorage site choice.* Mol Cell, 2014. **55**(4): p. 626-39.
12. Kalocsay, M., N.J. Hiller, and S. Jentsch, *Chromosome-wide Rad51 spreading and SUMO-H2A.Z-dependent chromosome fixation in response to a persistent DNA double-strand break.* Mol Cell, 2009. **33**(3): p. 335-43.
13. Taty-Taty, G.C., et al., *H2A.Z depletion impairs proliferation and viability but not DNA double-strand breaks repair in human immortalized and tumoral cell lines.* Cell Cycle, 2014. **13**(3): p. 399-407.
14. van Attikum, H., O. Fritsch, and S.M. Gasser, *Distinct roles for SWR1 and INO80 chromatin remodeling complexes at chromosomal double-strand breaks.* EMBO J, 2007. **26**(18): p. 4113-25.
15. Xu, Y., et al., *Histone H2A.Z controls a critical chromatin remodeling step required for DNA double-strand break repair.* Mol Cell, 2012. **48**(5): p. 723-33.
16. Barski, A., et al., *High-resolution profiling of histone methylations in the human genome.* Cell, 2007. **129**(4): p. 823-37.
17. Kysela, B., M. Chovanec, and P.A. Jeggo, *Phosphorylation of linker histones by DNA-dependent protein kinase is required for DNA ligase IV-dependent ligation in the presence of histone H1.* Proc Natl Acad Sci U S A, 2005. **102**(6): p. 1877-82.
18. Machida, S., et al., *Nap1 stimulates homologous recombination by RAD51 and RAD54 in higher-ordered chromatin containing histone H1.* Sci Rep, 2014. **4**: p. 4863.
19. Rosidi, B., et al., *Histone H1 functions as a stimulatory factor in backup pathways of NHEJ.* Nucleic Acids Res, 2008. **36**(5): p. 1610-23.
20. Sellou, H., et al., *The poly(ADP-ribose)-dependent chromatin remodeler Alc1 induces local chromatin relaxation upon DNA damage.* Mol Biol Cell, 2016. **27**(24): p. 3791-3799.
21. Strickfaden, H., et al., *Poly(ADP-ribosyl)ation-dependent Transient Chromatin Decondensation and Histone Displacement following Laser Microirradiation.* J Biol Chem, 2016. **291**(4): p. 1789-802.
22. Thorslund, T., et al., *Histone H1 couples initiation and amplification of ubiquitin signalling after DNA damage.* Nature, 2015. **527**(7578): p. 389-93.
23. Krishnakumar, R., et al., *Reciprocal binding of PARP-1 and histone H1 at promoters specifies transcriptional outcomes.* Science, 2008. **319**(5864): p. 819-21.
24. Millan-Arino, L., et al., *Mapping of six somatic linker histone H1 variants in human breast cancer cells uncovers specific features of H1.2.* Nucleic Acids Res, 2014. **42**(7): p. 4474-93.

25. Khurana, S., et al., *A macrohistone variant links dynamic chromatin compaction to BRCA1-dependent genome maintenance.* Cell Rep, 2014. **8**(4): p. 1049-62.
26. Leung, J.W., et al., *ZMYM3 regulates BRCA1 localization at damaged chromatin to promote DNA repair.* Genes Dev, 2017. **31**(3): p. 260-274.
27. Timinszky, G., et al., *A macrodomain-containing histone rearranges chromatin upon sensing PARP1 activation.* Nat Struct Mol Biol, 2009. **16**(9): p. 923-9.
28. Xu, C., et al., *The histone variant macroH2A1.1 is recruited to DSBs through a mechanism involving PARP1.* FEBS Lett, 2012. **586**(21): p. 3920-5.
29. Chen, H., et al., *MacroH2A1.1 and PARP-1 cooperate to regulate transcription by promoting CBP-mediated H2B acetylation.* Nat Struct Mol Biol, 2014. **21**(11): p. 981-9.
30. Gamble, M.J., et al., *The histone variant macroH2A1 marks repressed autosomal chromatin, but protects a subset of its target genes from silencing.* Genes Dev, 2010. **24**(1): p. 21-32.
31. Tropberger, P., et al., *Regulation of transcription through acetylation of H3K122 on the lateral surface of the histone octamer.* Cell, 2013. **152**(4): p. 859-72.
32. Botuyan, M.V., et al., *Structural basis for the methylation state-specific recognition of histone H4-K20 by 53BP1 and Crb2 in DNA repair.* Cell, 2006. **127**(7): p. 1361-73.
33. Giannattasio, M., et al., *The DNA damage checkpoint response requires histone H2B ubiquitination by Rad6-Bre1 and H3 methylation by Dot1.* J Biol Chem, 2005. **280**(11): p. 9879-86.
34. Huyen, Y., et al., *Methylated lysine 79 of histone H3 targets 53BP1 to DNA double-strand breaks.* Nature, 2004. **432**(7015): p. 406-11.
35. Vlaming, H., et al., *Direct screening for chromatin status on DNA barcodes in yeast delineates the regulome of H3K79 methylation by Dot1.* Elife, 2016. **5**.
36. Wakeman, T.P., et al., *Bat3 facilitates H3K79 dimethylation by DOT1L and promotes DNA damage-induced 53BP1 foci at G1/G2 cell-cycle phases.* EMBO J, 2012. **31**(9): p. 2169-81.
37. Wysocki, R., et al., *Role of Dot1-dependent histone H3 methylation in G1 and S phase DNA damage checkpoint functions of Rad9.* Mol Cell Biol, 2005. **25**(19): p. 8430-43.
38. Strikoudis, A., et al., *Regulation of transcriptional elongation in pluripotency and cell differentiation by the PHD-finger protein Phf5a.* Nat Cell Biol, 2016. **18**(11): p. 1127-1138.
39. Dulev, S., et al., *SET8 methyltransferase activity during the DNA double-strand break response is required for recruitment of 53BP1.* EMBO Rep, 2014. **15**(11): p. 1163-74.
40. Hartlerode, A.J., et al., *Impact of histone H4 lysine 20 methylation on 53BP1 responses to chromosomal double strand breaks.* PLoS One, 2012. **7**(11): p. e49211.
41. Hsiao, K.Y. and C.A. Mizzen, *Histone H4 deacetylation facilitates 53BP1 DNA damage signaling and double-strand break repair.* J Mol Cell Biol, 2013. **5**(3): p. 157-65.
42. Oda, H., et al., *Regulation of the histone H4 monomethylase PR-Set7 by CRL4(Cdt2)-mediated PCNA-dependent degradation during DNA damage.* Mol Cell, 2010. **40**(3): p. 364-76.
43. Pei, H., et al., *MMSET regulates histone H4K20 methylation and 53BP1 accumulation at DNA damage sites.* Nature, 2011. **470**(7332): p. 124-8.
44. Sanders, S.L., et al., *Methylation of histone H4 lysine 20 controls recruitment of Crb2 to sites of DNA damage.* Cell, 2004. **119**(5): p. 603-14.

45. Tuzon, C.T., et al., *Concerted activities of distinct H4K20 methyltransferases at DNA double-strand breaks regulate 53BP1 nucleation and NHEJ-directed repair.* Cell Rep, 2014. **8**(2): p. 430-8.
46. Jiang, Y., et al., *Local generation of fumarate promotes DNA repair through inhibition of histone H3 demethylation.* Nat Cell Biol, 2015. **17**(9): p. 1158-68.
47. Wu, W., et al., *Interaction of BARD1 and HP1 Is Required for BRCA1 Retention at Sites of DNA Damage.* Cancer Res, 2015. **75**(7): p. 1311-21.
48. Young, L.C., D.W. McDonald, and M.J. Hendzel, *Kdm4b histone demethylase is a DNA damage response protein and confers a survival advantage following gamma-irradiation.* J Biol Chem, 2013. **288**(29): p. 21376-88.
49. Ayrapetov, M.K., et al., *DNA double-strand breaks promote methylation of histone H3 on lysine 9 and transient formation of repressive chromatin.* Proc Natl Acad Sci U S A, 2014. **111**(25): p. 9169-74.
50. Moyal, L., et al., *Requirement of ATM-dependent monoubiquitylation of histone H2B for timely repair of DNA double-strand breaks.* Mol Cell, 2011. **41**(5): p. 529-42.
51. Nakamura, K., et al., *Regulation of homologous recombination by RNF20-dependent H2B ubiquitination.* Mol Cell, 2011. **41**(5): p. 515-28.
52. Heintzman, N.D., et al., *Distinct and predictive chromatin signatures of transcriptional promoters and enhancers in the human genome.* Nat Genet, 2007. **39**(3): p. 311-8.
53. Cao, L.L., et al., *ATM-mediated KDM2A phosphorylation is required for the DNA damage repair.* Oncogene, 2016. **35**(3): p. 301-13.
54. Fnu, S., et al., *Methylation of histone H3 lysine 36 enhances DNA repair by nonhomologous end-joining.* Proc Natl Acad Sci U S A, 2011. **108**(2): p. 540-5.
55. Kuo, A.J., et al., *NSD2 links dimethylation of histone H3 at lysine 36 to oncogenic programming.* Mol Cell, 2011. **44**(4): p. 609-20.
56. Streubel, G., et al., *The H3K36me2 Methyltransferase Nsd1 Demarcates PRC2-Mediated H3K27me2 and H3K27me3 Domains in Embryonic Stem Cells.* Mol Cell, 2018. **70**(2): p. 371-379 e5.
57. Aymard, F., et al., *Transcriptionally active chromatin recruits homologous recombination at DNA double-strand breaks.* Nat Struct Mol Biol, 2014. **21**(4): p. 366-74.
58. Carvalho, S., et al., *SETD2 is required for DNA double-strand break repair and activation of the p53-mediated checkpoint.* Elife, 2014. **3**: p. e02482.
59. Pfister, S.X., et al., *SETD2-dependent histone H3K36 trimethylation is required for homologous recombination repair and genome stability.* Cell Rep, 2014. **7**(6): p. 2006-18.
60. Bird, A.W., et al., *Acetylation of histone H4 by Esa1 is required for DNA double-strand break repair.* Nature, 2002. **419**(6905): p. 411-5.
61. Murr, R., et al., *Histone acetylation by Trrap-Tip60 modulates loading of repair proteins and repair of DNA double-strand breaks.* Nat Cell Biol, 2006. **8**(1): p. 91-9.
62. Tamburini, B.A. and J.K. Tyler, *Localized histone acetylation and deacetylation triggered by the homologous recombination pathway of double-strand DNA repair.* Mol Cell Biol, 2005. **25**(12): p. 4903-13.
63. Lopez-Atalaya, J.P., et al., *Genomic targets, and histone acetylation and gene expression profiling of neural HDAC inhibition.* Nucleic Acids Res, 2013. **41**(17): p. 8072-84.
64. Krishnan, V., et al., *Histone H4 lysine 16 hypoacetylation is associated with defective DNA repair and premature senescence in Zmpste24-deficient mice.* Proc Natl Acad Sci U S A, 2011. **108**(30): p. 12325-30.

65. Miller, K.M., et al., *Human HDAC1 and HDAC2 function in the DNA-damage response to promote DNA nonhomologous end-joining.* Nat Struct Mol Biol, 2010. **17**(9): p. 1144-51.
66. Sharma, G.G., et al., *MOF and histone H4 acetylation at lysine 16 are critical for DNA damage response and double-strand break repair.* Mol Cell Biol, 2010. **30**(14): p. 3582-95.
67. Tang, J., et al., *Acetylation limits 53BP1 association with damaged chromatin to promote homologous recombination.* Nat Struct Mol Biol, 2013. **20**(3): p. 317-25.
68. Taylor, G.C., et al., *H4K16 acetylation marks active genes and enhancers of embryonic stem cells, but does not alter chromatin compaction.* Genome Res, 2013. **23**(12): p. 2053-65.
69. Das, C., et al., *CBP/p300-mediated acetylation of histone H3 on lysine 56.* Nature, 2009. **459**(7243): p. 113-7.
70. Tjeertes, J.V., K.M. Miller, and S.P. Jackson, *Screen for DNA-damage-responsive histone modifications identifies H3K9Ac and H3K56Ac in human cells.* EMBO J, 2009. **28**(13): p. 1878-89.
71. Toiber, D., et al., *SIRT6 recruits SNF2H to DNA break sites, preventing genomic instability through chromatin remodeling.* Mol Cell, 2013. **51**(4): p. 454-68.
72. Vempati, R.K., et al., *p300-mediated acetylation of histone H3 lysine 56 functions in DNA damage response in mammals.* J Biol Chem, 2010. **285**(37): p. 28553-64.
73. Yuan, J., et al., *Histone H3-K56 acetylation is important for genomic stability in mammals.* Cell Cycle, 2009. **8**(11): p. 1747-53.
74. Lo, K.A., et al., *Genome-wide profiling of H3K56 acetylation and transcription factor binding sites in human adipocytes.* PLoS One, 2011. **6**(6): p. e19778.
75. Cheung, W.L., et al., *Phosphorylation of histone H4 serine 1 during DNA damage requires casein kinase II in S. cerevisiae.* Curr Biol, 2005. **15**(7): p. 656-60.
76. Utley, R.T., et al., *Regulation of NuA4 histone acetyltransferase activity in transcription and DNA repair by phosphorylation of histone H4.* Mol Cell Biol, 2005. **25**(18): p. 8179-90.
77. Fradet-Turcotte, A., et al., *53BP1 is a reader of the DNA-damage-induced H2A Lys 15 ubiquitin mark.* Nature, 2013. **499**(7456): p. 50-4.
78. Pellegrino, S., et al., *Replication-Coupled Dilution of H4K20me2 Guides 53BP1 to Pre-replicative Chromatin.* Cell Rep, 2017. **19**(9): p. 1819-1831.
79. Sun, Z.W. and C.D. Allis, *Ubiquitination of histone H2B regulates H3 methylation and gene silencing in yeast.* Nature, 2002. **418**(6893): p. 104-8.
80. Zeng, M., et al., *CRL4(Wdr70) regulates H2B monoubiquitination and facilitates Exo1-dependent resection.* Nat Commun, 2016. **7**: p. 11364.
81. Zhou, L., et al., *Evidence that ubiquitylated H2B corrals hDot1L on the nucleosomal surface to induce H3K79 methylation.* Nat Commun, 2016. **7**: p. 10589.
82. Park, S.H., et al., *Type I interferons and the cytokine TNF cooperatively reprogram the macrophage epigenome to promote inflammatory activation.* Nat Immunol, 2017. **18**(10): p. 1104-1116.
83. Wang, Z., et al., *Combinatorial patterns of histone acetylations and methylations in the human genome.* Nat Genet, 2008. **40**(7): p. 897-903.
84. Butler, L.R., et al., *The proteasomal de-ubiquitinating enzyme POH1 promotes the double-strand DNA break response.* EMBO J, 2012. **31**(19): p. 3918-34.

85. Gudjonsson, T., et al., *TRIP12 and UBR5 suppress spreading of chromatin ubiquitylation at damaged chromosomes.* Cell, 2012. **150**(4): p. 697-709.
86. Rogakou, E.P., et al., *DNA double-stranded breaks induce histone H2AX phosphorylation on serine 139.* J Biol Chem, 1998. **273**(10): p. 5858-68.
87. Iacovoni, J.S., et al., *High-resolution profiling of gammaH2AX around DNA double strand breaks in the mammalian genome.* EMBO J, 2010. **29**(8): p. 1446-57.
